# Characterization of the Key Determinants of Phd Antitoxin Mediated Doc Toxin Inactivation in *Salmonella*

**DOI:** 10.1021/acschembio.2c00276

**Published:** 2022-06-01

**Authors:** Guilherme V. de Castro, Dennis J. Worm, Grzegorz J. Grabe, Fiona C. Rowan, Lucy Haggerty, Ana L. de la Lastra, Oana Popescu, Sophie Helaine, Anna Barnard

**Affiliations:** † Department of Chemistry, Molecular Sciences Research Hub, Imperial College London, 82 Wood Lane, London W12 0BZ, United Kingdom; ‡ Department of Microbiology, Harvard Medical School, 4 Blackfan Circle, Boston, Massachusetts 02115, United States

## Abstract

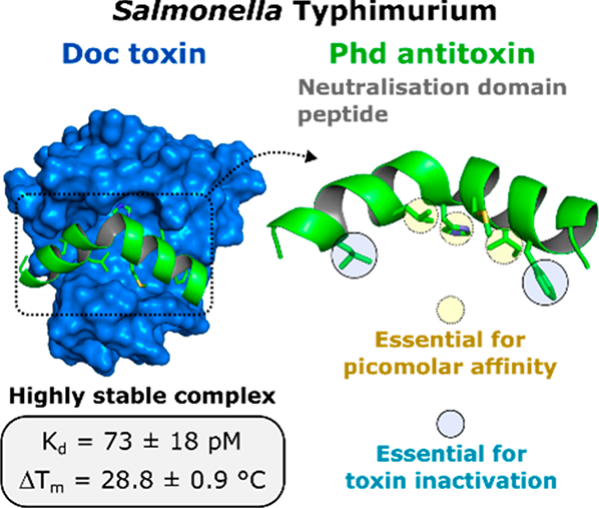

In the search for novel antimicrobial therapeutics, toxin-antitoxin (TA) modules are promising yet underexplored targets for overcoming antibiotic failure. The bacterial toxin Doc has been associated with the persistence of *Salmonella* in macrophages, enabling its survival upon antibiotic exposure. After developing a novel method to produce the recombinant toxin, we have used antitoxin-mimicking peptides to thoroughly investigate the mechanism by which its cognate antitoxin Phd neutralizes the activity of Doc. We reveal insights into the molecular detail of the Phd–Doc relationship and discriminate antitoxin residues that stabilize the TA complex from those essential for inhibiting the activity of the toxin. Coexpression of Doc and antitoxin peptides in *Salmonella* was able to counteract the activity of the toxin, confirming our *in vitro* results with equivalent sequences. Our findings provide key principles for the development of chemical tools to study and therapeutically interrogate this important class of protein–protein interactions.

## Introduction

Bacterial protein–protein interactions (PPIs) are involved in a multitude of vital cellular processes and are hence increasingly being investigated as antibiotic targets to tackle the exacerbating problem of antimicrobial resistance.^1,2^ One family of PPIs that are abundant in prokaryotes but remain significantly underexplored as therapeutic targets are type II toxin-antitoxin (TA) modules.^3^ These systems consist of toxin and antitoxin proteins that form a tight PPI.^4^ They were originally described as plasmid addiction modules;^5,6^ however, in recent years their prevalence within bacterial chromosomes has become evident. Type II TA systems are involved in modulation of growth in response to nutritional stress,^7^ abortion of phage infection,^8,9^ and survival to host immune defense.^10,11^ Activation of type II TA modules is suggested to be initiated by degradation of the antitoxin upon stress, which releases the toxin.^12^ The active toxin then stalls bacterial growth in a reversible manner by interfering with, for example, DNA replication or translation.^13^

Despite their important role as stress-responsive elements, the potential of type II toxin-antitoxin systems as targets for antimicrobial therapies is yet to be fully validated. Internalization of *Salmonella enterica* serovar Typhimurium (*S.* Typhimurium) by macrophages triggers the formation of antibiotic-tolerant persisters. Knockout of the *phd*-*doc* TA module was shown to have a substantial negative effect on numbers of persisters recovered from macrophages,^10^ with lower persister numbers suggested to lead to reduced *Salmonella* survival and reinfection. This TA system consists of the antitoxin Phd, which binds via its C-terminal helical domain to the toxin Doc.^14^ In *Escherichia coli*, the toxicity of Doc was linked to its ability to phosphorylate the translation elongation factor EF-Tu on a single threonine residue, rendering it incapable of binding aminoacylated tRNAs, thus halting protein synthesis.^15^

Before validation of type II TA systems as therapeutic targets based on their growth-modulating activity, detailed characterization of the interaction between toxins and antitoxins is required in order to develop toxin inhibitors. Toxin inhibitors would potentially be able to significantly reduce persister formation, which in a cotreatment with antibiotic would allow for a more complete clearance of the bacterial infection and prevention of infection recurrence. In this study, we report the first biochemical and biophysical characterization of the *S.* Typhimurium Phd-Doc PPI. Considering the challenges for the recombinant production of active bacterial toxins, we developed a novel approach to obtain Doc, which can be readily adapted to other TA systems. We then synthesized multiple peptides mimicking the Phd primary toxin binding-domain and used them as chemical tools to assess the role of specific residues and regions of the antitoxin on Doc toxin neutralization. Substitution of some residues led to poor or no inhibition of Doc, despite the formation of high-affinity complexes with picomolar dissociation constants, suggesting that toxin neutralization is achieved by mechanisms beyond high affinity interactions. Peptide sequences showing high *in vitro* inhibitory profiles counteracted Doc toxicity when expressed in *S.* Typhimurium, demonstrating excellent correlation with *in vitro* results. This work provides key insights for the future development of effective Doc inhibitors as both chemical tools to study the role of the toxin in *Salmonella* and as potential antimicrobial agents.

## Results

### Coexpression of a Mutant EF-Tu Enables the Large-Scale Expression of Wild-Type Doc

Characterization of the *S.* Typhimurium Phd-Doc PPI required isolation of the toxin (hereon referred as Doc_STm_) for biophysical and biochemical evaluation. However, one of the major challenges of studying TA modules is the purification of active toxins.^16^ In bacterial expression systems toxin expression severely inhibits normal growth, resulting in production of only trace amounts of the toxin of interest. To overcome these issues, common strategies include inactivation of the toxin by site-directed mutagenesis or, in the case of type II TA systems, coexpression with the antitoxin.^16,17^ The latter approach requires subsequent denaturation of the stable toxin-antitoxin complex to isolate the toxin, followed by refolding to restore its active conformation. As both strategies were previously used to produce recombinant Doc from the *E. coli* bacteriophage P1 (Doc_P1_),^16,17^ we attempted similar approaches to obtain the *S.* Typhimurium homologue. However, expression of an inactive Doc_STm_^H68Y^ mutant resulted in insoluble protein and refolding of Doc_STm_ from denatured toxin-antitoxin complex proved poorly reproducible and low yielding (ESI Figure S1).

**Figure 1:**
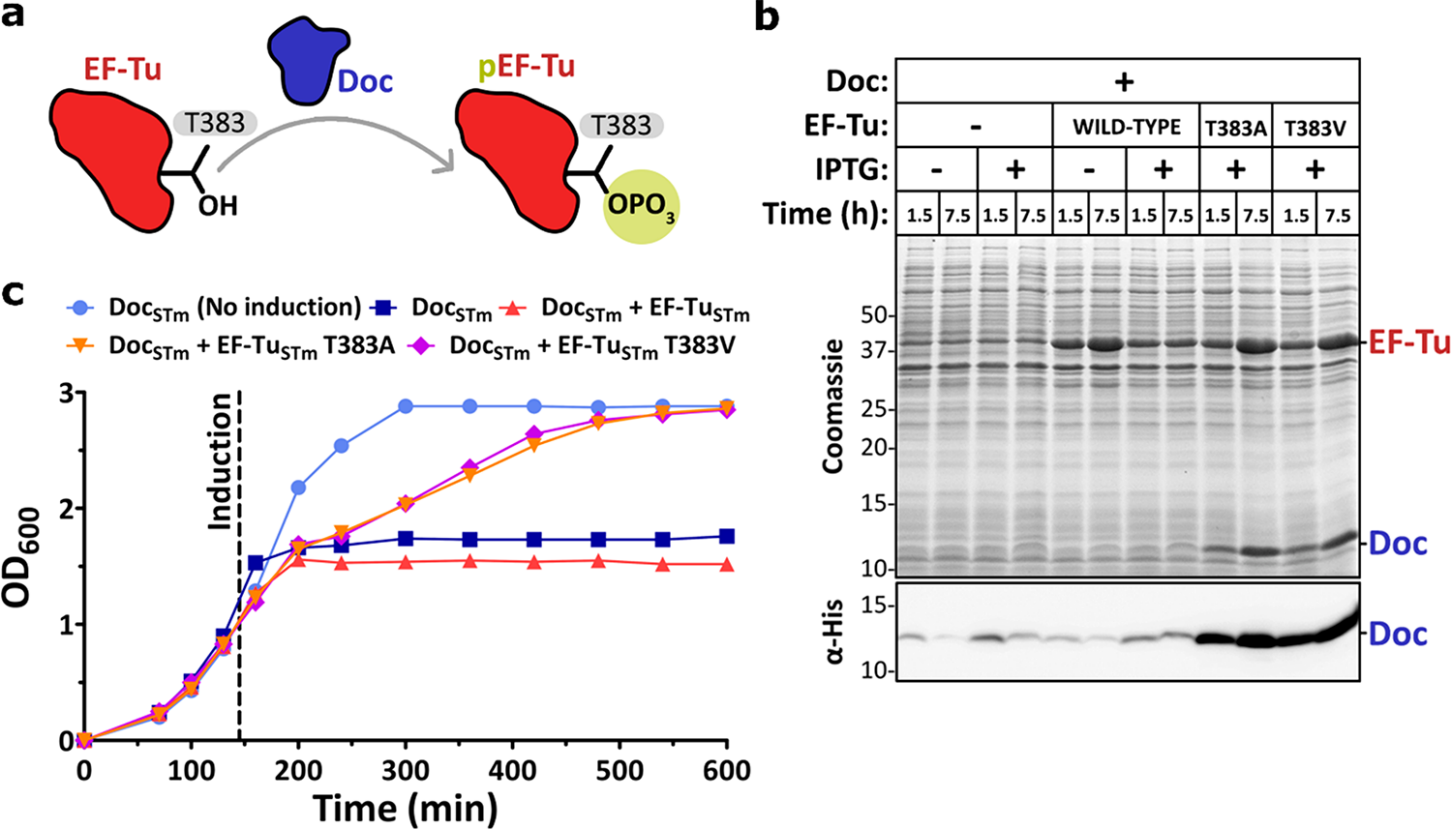
Recombinant expression of the Doc_STm_toxin in bacteria. (a) Schematic representation of the phosphorylation of EF-Tu_STm_ residue T383 by the Doc_STm_ toxin. (b) SDS-PAGE (upper) and Western blot (lower) analysis of Doc_STm_ expression trials varying the addition of IPTG and the presence of EF-Tu_STm_ encoding plasmids: wild-type, T383A, and T383V. Time-points correspond to aliquots collected 1.5 and 7.5 h after induction. l-Arabinose (inducer of EF-Tu variants) was added to all samples. (c) Growth curves of BL21-AI *E. coli* expressing Doc_STm_ (blue) or coexpressing Doc_STm_ with EF-Tu_STm_ wild-type (red), T383A (orange), and T383V (violet). A control sample of cells carrying the Doc_STm_ vector in the absence of IPTG (light blue) was measured for comparison. l-Arabinose was added in all samples.

To overcome the issues observed with both approaches, we developed a novel strategy to obtain the wild-type Doc_STm_ without the need of refolding. Doc toxicity is associated with its ability to block protein translation via phosphorylation of a highly conserved threonine (T383) residue on EF-Tu (Figure 1a).^15,18^ We therefore hypothesized that coexpression of an EF-Tu variant that is still active as an elongation factor, but cannot be phosphorylated by the toxin, could be an effective way to express Doc without affecting cell growth. To test this, vectors containing wild-type, T383A, and T383V variants of EF-Tu_STm_ were generated. As expected, when Doc_STm_ expression was initiated, coexpression of the wild-type EF-Tu_STm_ did not rescue bacterial growth. However, when EF-Tu_STm_ T383A or T383V variants were used, cell growth was maintained despite a significant increase in Doc expression levels (Figure 1b,c). Using our novel method, we successfully purified significant amounts of Doc_STm_ (3.0 mg of Doc_STm_ per liter of culture) in a highly reproducible manner.

### C-Terminal Phd Peptide Forms a Highly Stable Complex with Doc

**Figure 2:**
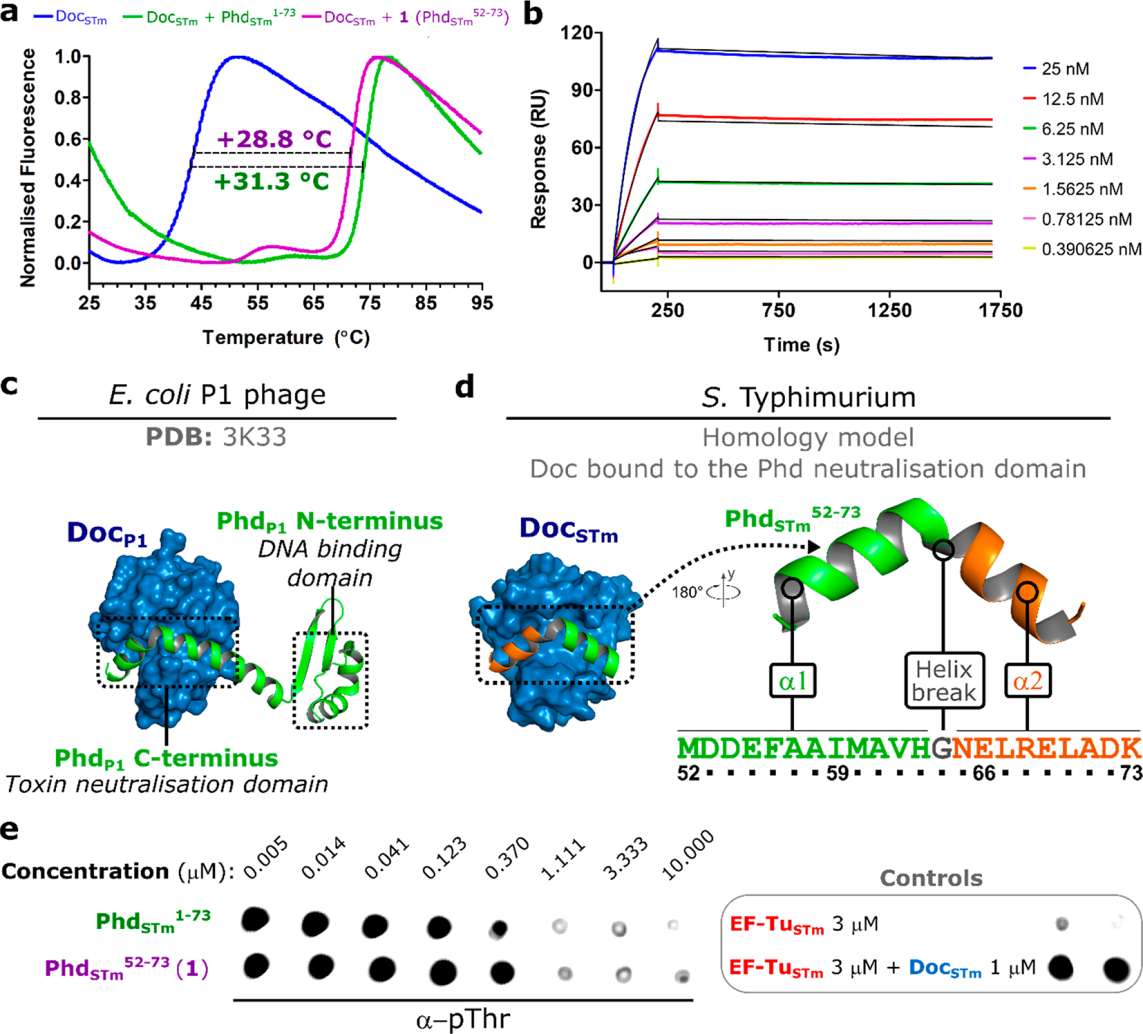
Characterization of the Phd full-length protein and C-terminal peptide as Doc inhibitors. (a) DSF measurements of Doc_STm_ at 5 μM free in solution (blue) and in the presence of 50 μM of Phd_STm_ (green) or peptide **1** (purple). (b) Sensorgrams of the interaction between Doc_STm_ and increasing concentrations of Phd_STm_measured by multicycle kinetics (MCK) SPR experiments. (c) Primary interaction between Doc_P1_ and Phd_P1_ from the previously reported crystallized complex (PDB: 3K33). (d) Homology model of Doc_STm_ bound to the C-terminal domain of Phd_STm_ (left). The sequence and bound conformation of the Phd_P1_ C-terminal domain are shown on the right, two helices (α1 and α2, respectively, in green and orange) are separated by Gly64 (gray). (e) Dot blot detection of phosphorylated EF-Tu_STm_ in the presence of Phd_STm_ (green) or peptide **1** (purple). Controls in the absence of inhibitors are shown on the right chart. In all cases the final concentrations of Doc_STm_, EF-Tu_STm_ and ATP were, respectively, 1 μM, 3 μM, and 1 mM. Phd_STm_ protein and peptide **1** were tested at eight concentrations, ranging from 10 μM down to approximately 5 nM (3-fold dilutions).

To assess the activity of recombinant Doc, phosphorylation assays were carried out with EF-Tu_STm_ and Doc_STm_ and analyzed by dot blot with an antiphosphothreonine antibody (α-pThr). Doc_STm_ was active with specific phosphorylation of EF-Tu_STm_ observed in the presence of ATP (ESI Figure S2). As expected, no phosphorylation was detected in the presence of Phd_STm_ or with the EF-Tu_STm_ T383V variant (ESI Figure S2), in agreement with previously reported observations from the *E. coli* P1 phage homologues.^15,18^ The presence of Phd_STm_ resulted in a large thermal stabilization of Doc_STm_, verified by a positive melting temperature (*T*_m_) shift of 31.3 ± 0.9 °C in differential scanning fluorimetry (DSF) experiments (Figure 2a). This highly stable complex was also observed in surface plasmon resonance (SPR) experiments, where the complex showed a dissociation constant (*K*_d_) of 61 ± 19 pM and a half-life of nearly 7 h (ESI Table S1 and Figure 2b).

In order to characterize the key features responsible for the high affinity and inhibitory activity of Phd_STm_ antitoxin toward Doc_STm_ toxin, we aimed to use antitoxin-mimetic peptides as chemical tools, as previous studies revealed that the C-terminal domain of the antitoxin is responsible for the neutralization of the toxin (Figure 2c).^19^ These peptides could then also act as templates for the generation of future Doc_STm_ inhibitors. We therefore developed a homology model of Doc_STm_ bound to C-terminal residues 52–73 of Phd_STm_. The model was based on the published crystal structure (PDB: 3K33)^20^ of Doc_P1_ in complex with Phd from *E. coli* P1 phage (Phd_P1_) and suggested a helical conformation of Phd_STm_ upon binding (Figure 2d), where two α-helices (termed α1 and α2 here) are separated by a structural “kink” at Gly64. To validate the model, region 52–73 of Phd_STm_ was synthesized by automated microwave-assisted solid-phase peptide synthesis (SPPS). To allow spectrophotometric quantification of the concentration, a tryptophan residue was introduced at the N-terminus of the peptide. A standard Fmoc/*t*Bu strategy was used and the N-terminus was acetylated, resulting in the isolation of peptide **1** (Phd^52–73^, Ac-WMDDEFAAIMAVHGNELRELADK-OH, ESI Tables S2 and S3) in high purity.

Like the full-length Phd antitoxin, peptide **1** was found to form a tight complex with Doc_STm_, leading to a thermal stabilization of 28.8 ± 0.9 °C, a *K*_d_ of 73 ± 18 pM and comparable inhibition of the kinase activity (Figure 2 and ESI Table S1). While circular dichroism (CD) spectroscopy revealed that peptide **1** is predominantly disordered in phosphate buffer, we observed a pronounced helical fold in buffer containing 30% (v/v) of the secondary structure inducer trifluoroethanol (TFE), suggesting that peptide **1** can likely assume the binding conformation proposed in the homology model (ESI Figure S3).

### Hot Spot Residues and Minimal Binding Sequence of the Phd Neutralization Domain

**Figure 3:**
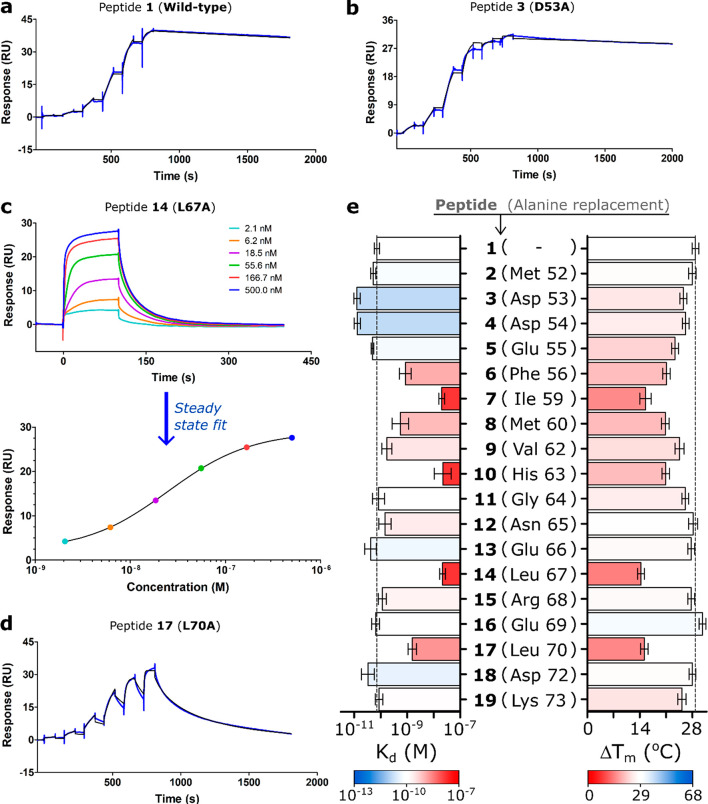
Alanine scanning of the Phd antitoxin neutralization domain. SPR sensorgrams of the interaction between Doc_STm_ and peptides **1** (a), **3** (b), **14** (c, upper graph), and **17** (d). MCK experiments were used for **14**, where the *K*_d_ was determined from a steady-state fit (c, bottom graph), while the remaining sensorgrams shown were obtained via single-cycle kinetics (SCK) experiments (kinetic fit is shown as black lines over the sensorgrams). (e) Summary of the *K*_d_ (SPR) and Δ*T*_m_ (DSF) values of the interaction of Doc_STm_ to peptide **1** and alanine scanning analogues **2**–**19** in the left and right bar charts, respectively.

To dissect the features responsible for the high affinity between peptide **1** and Doc_STm_, we first sought to identify the residues that provide a higher contribution to the stability of the complex (known as “hot spots”).^21^ Thus, we synthesized peptide variants of **1** with single alanine substitution for each residue (**2**–**19**) and assessed their interaction with Doc_STm_ by DSF and SPR. In total, six hot spot residues were identified (Figure 3 and ESI Table S1). When compared to wild-type peptide **1**, the substitution of Phe56 (**6**), Met60 (**8**), and Leu70 (**17**) caused an approximate 8- to 30-fold decrease in *K*_d_, while the replacement of residues Ile59 (**7**), His63 (**10**), and Leu67 (**14**) resulted in an even greater 300-fold decrease in affinity. In the DSF experiments, these six peptides had the smallest stabilization effect on Doc_STm_ (Δ*T*_m_ ≈ 14–21 °C) when compared with other alanine variants (Δ*T*_m_ ≈ 23–30 °C) (Figure 3e), further supporting their important role in the stability of the TA complex. These results strongly agree with the binding conformation observed in the homology model, as all hot spot residues were found to directly interact with Doc_STm_ (ESI Figure S4).

Although most nonhot spot peptides possessed similar *K*_d_ values to peptide **1**, surprisingly, a significant increase in affinity was observed for two peptides. Alanine replacements of Asp53 (**3**) and Asp54 (**4**) residues resulted in a gain of affinity of greater than 5-fold when compared to peptide **1** (Figure 3e and ESI Table S1). In both cases, this could be a result of multiple factors, as improvements in both *k*_on_ (∼4-fold) and *k*_off_ (∼1.5-fold) were observed when compared with **1** (Table S1). We hypothesized that the gain in affinity was mainly due to an increase in peptide helicity, favoring the bound conformation (faster *k*_on_). Ala possesses the highest helix propensity among all natural amino acids while Asp (deprotonated) is known as one of the poorest helix inducers.^22^ We could not detect a greater helicity for peptides **3** and **4** (ESI Figure S3) compared with peptide **1**, which may be due to the challenges of detecting such subtle differences (replacement of one residue in a 23-mer peptide) in CD experiments. Nevertheless, beyond the hot spot modifications, particular focus will also be given to these sequences in the subsequent experiments to verify if these improvements could be relevant in the inhibitory activity of the peptides.

**Figure 4:**
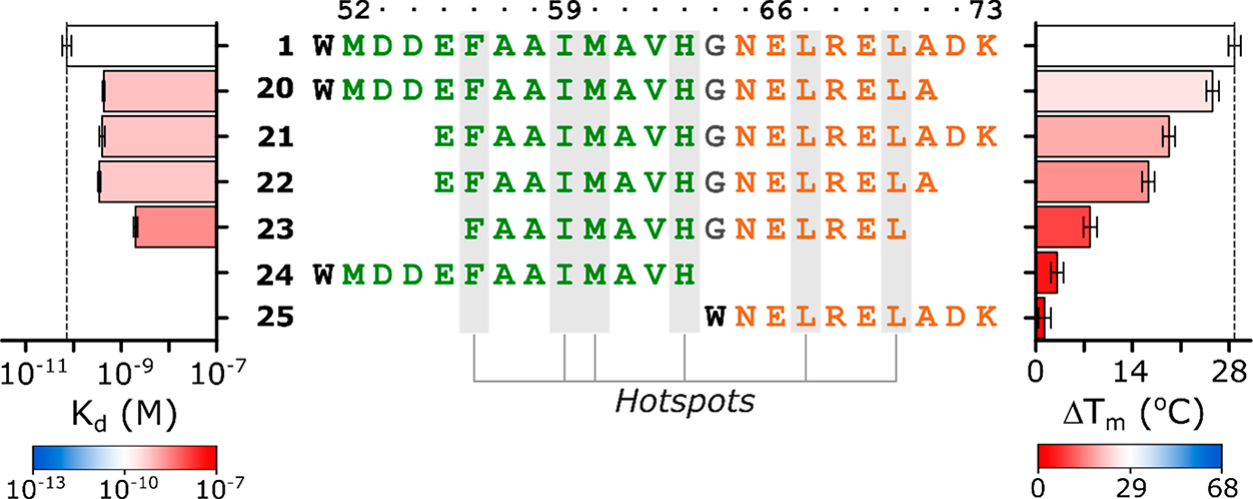
Interaction of truncated analogues of peptide **1** to Doc. Summary of the *K*_d_ (SPR) and Δ*T*_m_ (DSF) values of the interaction of Doc to peptide **1** and truncated analogues **20**–**25** are shown in the left and right bar charts, respectively.

To determine the minimum binding sequence required for the interaction between Phd_STm_ and Doc_STm_, a set of truncated analogues of peptide **1** (Figure 4) was synthesized. Peptides **20**–**23** consist of deletions of terminal residues of the sequence, with peptide **23** representing the shortest sequence with all six hotspots still present (Phe56 to Leu70). Peptides **24** and **25** correspond, respectively, to the sequences of the α1 and α2 helices observed in the homology model (Figure 2d). The terminal truncations in **20**–**22** resulted in a loss of approximately 5-fold in *K*_d_ when compared with peptide **1**, while for peptide **23**, a 30-fold decrease was observed (Figure 4 and ESI Table S1), showing that none of these truncations abolished the interaction with the toxin. Surprisingly, an almost complete loss of affinity was observed for peptides **24** and **25**, as no measurable affinities could be obtained by SPR and only a slight thermal stabilization of approximately 3 °C was observed for peptide **24** by DSF (Figure 4). The simultaneous addition of both peptides **24** and **25** did not result in any further thermal stabilization (ESI Table S4 and Figure S5), excluding the possibility of a conditional binding mechanism where one helix is required to allow the interaction of the second. In contrast to **1**, only marginal helicity could be induced in **25** in the presence of 30% TFE, suggesting a high entropic penalty for it to assume the required helical conformation for interaction with Doc_STm_. However, for peptide **24** a pronounced helical fold could be observed in 30% TFE (ESI Figure S3), agreeing with the higher Δ*T*_m_ compared with **25** and suggesting that **24** can bind Doc_STm_, albeit with high micromolar or millimolar *K*_d_.

### “Gatekeeper” Hotspot Residues Are Crucial for Inactivation of Doc

**Figure 5:**
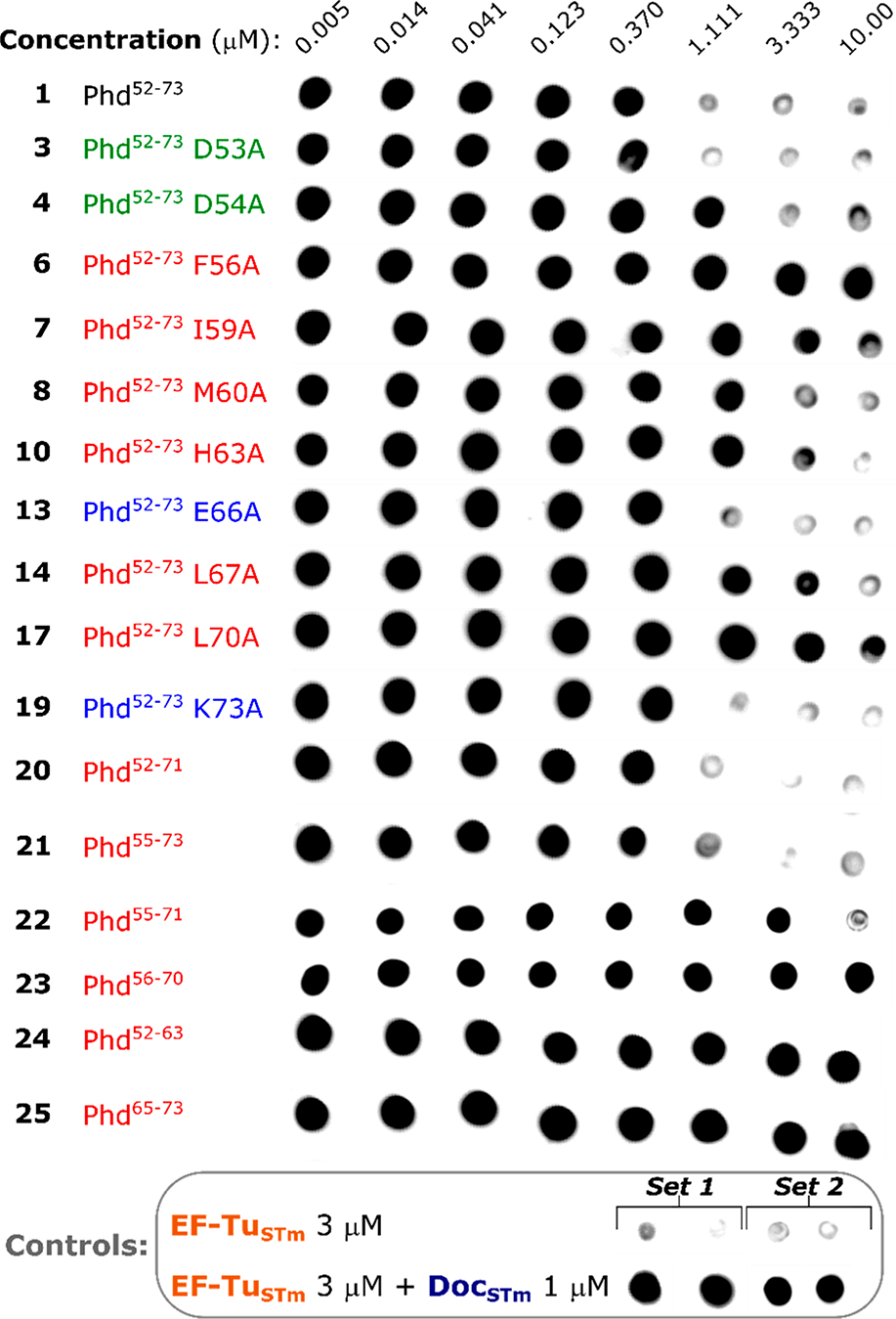
Inhibitory activity of the Phd antitoxin peptides. Dot blot detection of phosphorylated EF-Tu_STm_ in the presence of peptides **1**, **3**, **4**, **6**, **7**, **8**, **10**, **13**, **14**, **17**, **19**, **20**, **21**, **22**, **23**, **24** and **25**. All peptides were tested at eight concentrations, ranging from 10 μM to 5 nM (3-fold dilutions). Negative (EF-Tu_STm_ 3 μM) and positive (EF-Tu_STm_ 3 μM + Doc_STm_ 1 μM) phosphorylation controls of the assay are shown in the bottom chart. Set 2 of control samples were blotted simultaneously with the reaction using peptides **22** and **23**, while the set 1 of control samples were blotted simultaneously with samples from the reaction with the remaining peptides shown. Peptides with weaker affinity to Doc_STm_ than **1** are shown in red, peptides with similar affinity are shown in blue, and peptides with higher affinity are shown in green. A second, independent experiment is shown in ESI Figure S6.

To determine the effect of the alanine substitutions and sequence truncations on the antitoxin inhibitory activity, a subset of Phd_STm_ peptides was tested in the kinase activity assay. Peptide **1** was able to fully inhibit EF-Tu_STm_ phosphorylation when tested at concentrations equal or higher than the concentration of Doc_STm_ (1 μM) (Figure 2e). Alanine substituted peptides with weaker (**6**, **7**, **8**, **10**, **14**, **17**), similar (**13** and **19**), or higher (**3** and **4**) binding affinity to Doc_STm_ than the wild-type sequence (**1**) were selected for evaluation of their ability to neutralize Doc (Figure 5). Inhibition of EF-Tu_STm_ phosphorylation was achieved in most cases, albeit at varied concentration ranges. Peptides in the similar or higher affinity groups inhibited Doc_STm_ at similar concentrations to peptide **1** (Figure 5). However, most peptides in the weaker affinity group achieved Doc_STm_ inhibition only at higher concentrations, indicating a decrease in inhibitory activity when compared with **1** (Figure 5).

Surprisingly, the inhibitory activity of the weaker affinity peptides did not directly correlate with their respective *K*_d_ to Doc_STm_. Peptides **6** (F56A) and **17** (L70A) were noticeably among the poorest inhibitors of EF-Tu_STm_ phosphorylation, despite possessing affinities at least 12-fold greater to Doc_STm_ than peptides **7** (I59A), **10** (H63A) and **14** (L67A) (Figure 5). This result suggests that while certain residues on Phd_STm_ play a significant role in the overall stability of the TA complex, residues Phe56 and Leu70 (the first hot spots from both the N- and C-terminus) are essential for locking the toxin into an inactive conformation.

Both the C-terminally truncated **20** and N-terminally truncated **21** showed inhibition of Doc_STm_ comparable to **1**, while the weak/nonbinding peptides **24** and **25** were inactive. Interestingly, a severe loss of inhibitory activity was observed for the simultaneously N- and C-terminally truncated peptides **22** (*K*_d_ in the same range as **20** and **21**) and **23**, with the latter being inactive in all concentrations tested. Although hot spots Phe56 and Leu70 are still present on peptides **22** and **23**, these residues now occupy the peptide termini, suggesting that the orientation of these “gatekeeper” residues is key for the inhibition of Doc_STm_.

### Antitoxin Peptides Can Rescue *Salmonella* from Toxin-Induced Growth Arrest

**Figure 6:**
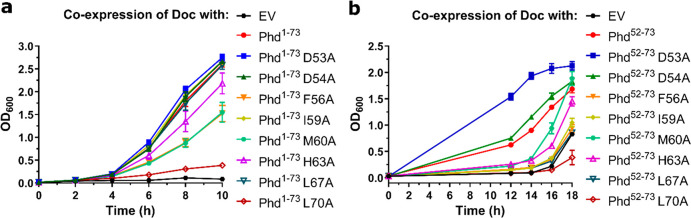
Phd proteins and peptides rescue *Salmonella* from Doc toxicity. Growth curves (measured as OD_600_) of (a) *S.* Typhimurium (14028) *Δphd-doc::Km* strains coexpressing Doc_STm_ (pBAD33) and different Phd_STm_^1–73^ protein mutants (pCA24N) and (b) *S.* Typhimurium (14028) *Δphd-doc::Km* strains coexpressing Doc_STm_ (pBAD33) and different Phd_STm_^52–73^ antitoxin peptides (pCA24N). OD_600_ at each time point is the average of three independent experiments. EV: empty vector.

In order to assess if Phd_STm_ peptides could neutralize Doc_STm_*in vivo*, growth rescue experiments were performed. Two sets of *S.* Typhimurium strains coexpressing Doc_STm_ and sequences corresponding to either the full-length antitoxin (Phd_STm_^1–73^) or solely its neutralization domain (Phd_STm_^52–73^) were generated. Beyond the wild-type sequences, both sets also included variants corresponding to the modifications present in peptides **3**, **4**, **6**, **7**, **8**, **10**, **14**, and **17**. The growth of all *S.* Typhimurium strains was monitored over time by measuring OD_600_. Control strains lacking the Doc_STm_ plasmid did not present any significant growth defects (ESI Figure S7a). In contrast, cells carrying solely the Doc_STm_ plasmid displayed a pronounced growth defect even after 18 h of culture (Figure 6a,b). Coexpression of Doc_STm_ with full-length, wild-type Phd_STm_^1–73^, as expected, prevented Doc-induced growth inhibition (Figure 6a). The peptide constituting the wild-type neutralization domain only rescued growth after a delay and longer incubation times of 16–18 h (Figure 6b and ESI Figure S7b). While expression of peptide **4** (D54A, *K*_d_ = 13 pM) restored bacterial growth following similar kinetics as the wild-type sequence (**1**, *K*_d_ = 73 pM), a faster recovery was achieved with peptide **3** (D53A, *K*_d_ = 13 pM). Coexpression of antitoxin peptides or proteins in which hotspot residues were mutated to alanine led to varying results. While Phd^52–73^-M60A peptide fully rescued *Salmonella* growth after 18 h of culture, Phd^52–73^-H63A peptide provided only a partial rescue and Phd^52–73^-I59A and Phd^52–73^-L67A peptides were unable to rescue growth. When expressing the corresponding alanine variants of full-length Phd^1–73^ antitoxin, Phd^1–73^-I59A, and Phd^1–73^-L67A were able to fully rescue growth after 10 h of culture, while Phd^1–73^-M60A and Phd^1–73^-H63A displayed only a partial growth rescue. Interestingly, antitoxin peptides with mutations of the gatekeeper residues (Phd^52–73^-F56A and Phd^52–73^-L70A) as well as the protein variant Phd^1–73^-L70A were not able to counteract Doc_STm_-induced growth inhibition of *Salmonella* (Figure 6a,b), and additionally, Phd^1–73^-F56A protein only showed a partial growth rescue. Overall, these findings confirm the importance of the gatekeeper residues Phe56 and Leu70 in Phd_STm_ antitoxin for Doc inhibition and demonstrate that high-affinity Phd_STm_ peptides can inactivate Doc_STm_ in *Salmonella*.

## Discussion

The isolation of wild-type toxins for binding and functional studies has been widely regarded as a challenging task, with the most successful methods involving expression in strains tolerant to the toxin^23^ or refolding of the denatured TA complex.^16^ Our method that relies on providing the cells with a mutated target immune to toxin activity, allows the usage of common bacterial expression strains and avoids time-consuming and poor-yielding refolding steps. Here, this was achieved by coexpression of an EF-Tu_STm_ mutant immune to toxin phosphorylation, allowing protein translation to proceed even when endogenous EF-Tu was inactivated. Although this strategy may not be applicable to TA families targeting a broader range of targets, such as ribonuclease toxins (e.g., HicA, MazF, RelE),^24^ this may be achievable for others (e.g., HipA^25,26^ and FicT^27^) and significantly improve toxin production for structural studies and screening campaigns.

Following the production of Doc_STm_ and confirmation of its activity, we measured its interaction with the Phd_STm_ antitoxin. Interestingly, the *K*_d_ for the complex was approximately 60 pM, remarkably tighter than the *K*_d_ of 350 nM reported for the *E. coli* P1 phage homologue (ESI Table S5),^20^ and among the highest measured affinities for any TA system. This discrepancy suggests that despite a conserved function, the ability of Phd to bind and neutralize Doc can vary between different species of bacteria. Although type II TA pairs are known to form tight complexes, for many families the precise binding affinities are still unknown, limiting comparisons across different modules.

We then focused on the Phd_STm_ C-terminal domain and designed antitoxin peptide **1**. Both its affinity for and inhibition of Doc_STm_ were comparable to the full-length Phd_STm_, validating that this region was sufficient to mimic the antitoxin. An alanine scan of **1** revealed six predominantly hydrophobic hot spot residues, which were all found to directly interact with Doc_STm_ via hydrophobic pockets present on the protein surface. Two of these hot spot positions could be also observed for the Doc_P1_-Phd_P1_ interaction (ESI Figure S1f), with Phe56 and Phe60 (corresponding to Phe56 and Met60 in Phd_STm_, respectively) previously shown to be important for antitoxin activity of Phd_P1_.^28^ Unexpectedly, the substitution of either Asp53 or Asp54 to alanine resulted in an increase in binding affinity. This finding shows that despite the remarkably high affinity of Phd_STm_^52–73^ to Doc_STm_, there are still opportunities for improvement, which can be exploited in the design of Doc_STm_ inhibitors.

When evaluating the effect of the substitutions and truncations on the inhibitory activity, we were surprised to observe residue-specific effects that were not directly correlated to their binding affinities. Unexpectedly, replacement of Phe56 or Leu70 caused the most drastic loss of inhibition activity among the alanine variants of hot spot residues despite others (Ile59, His63, and Leu67) making a greater contribution to the stability of the complex (*K*_d_ ≈ 20 nM upon alanine mutation). Beyond **6** (F56A, *K*_d_ = 852 pM) and **17** (L70A, *K*_d_ = 1.55 nM), peptides **22** (Phd^55–71^, *K*_d_ = 347 pM) and **23** (Phd^56–70^, *K*_d_ = 1.98 nM) with truncations simultaneously neighboring these two “gatekeeper” residues were also poor Doc_STm_ inhibitors. In this case, the loss of neutralization activity may result from a higher flexibility of Phe56 and Leu70 when occupying terminal positions.

Both Phe56 and Leu70 are highly conserved in the Phd antitoxin of multiple members of the Enterobacteriaceae family (*e.g. E. coli*, *Klebsiella pneumoniae*, *Citrobacter freundii*), suggesting that their key role in Doc neutralization is conserved among species beyond *S.* Typhimurium (ESI Figure S8). Our findings also reveal that the formation of a stable complex with picomolar affinities does not necessarily result in effective inhibition of Doc_STm_. In fact, similar to other type II TAs (e.g., HipA-HipB),^29^ the antitoxin does not directly bind to the catalytic site of the toxin. Previous studies with Doc_P1_ have shown that the Phd_P1_ neutralization domain prevents binding of ATP by the toxin^15^; however, no high resolution structures of ATP bound-toxin were reported to elucidate how this is achieved. The differences we observed between affinity and inhibitory activity imply that neutralization might be achieved by locking the toxin into a conformationally inactive state.

To evaluate the significance of our findings directly in *S.* Typhimurium, the growth of strains coexpressing Doc_STm_ and different Phd_STm_ constructs was monitored. In these experiments, Doc_STm_-induced growth inhibition was counteracted by coexpression with both the full-length and the C-terminal domain peptides of Phd_STm_ antitoxin. The rescue was faster with Phd^1–73^ antitoxin than with the Phd peptide, possibly due to different expression levels, the poor intracellular stability of peptides^30^ or the formation of additional neutralization interfaces with the full-length antitoxin. Nevertheless, observing neutralization of Doc_STm_ toxicity upon basal expression of Phd_STm_ peptides is encouraging, as it indicates that permeable compounds mimicking the binding mechanism of our peptides would successfully target and inhibit Doc. Furthermore, the replacement of the “gatekeeper” residues Phe56 and Leu70 had the most detrimental effect on counteracting Doc_STm_-induced growth inhibition in both the full-length Phd_STm_ antitoxin and the Phd_STm_ peptide, underlining the importance of theses residues for Doc_STm_ inhibition by Phd_STm_*in vivo*.

## Conclusion

We have carried out an extensive characterization of the Phd_STm_-Doc_STm_ pair, a contributor to macrophage-induced persistence of *S.* Typhimurium, focusing on main features responsible for its high stability and toxin inhibition. We have developed a new methodology for the purification of wild-type Doc_STm_, representing a novel strategy for the recombinant production of active bacterial toxins.

By using antitoxin-mimetic peptides as chemical tools to specifically study the inhibition of Doc_STm_ toxin by the neutralization domain of Phd_STm_ antitoxin, we found that six hot spot residues and the correct positioning of Phe56 and Leu70 to ensure appropriate orientation of these “gatekeeper” residues in Phd_STm_ are required for efficient inhibition of Doc_STm._ Our peptides additionally act as templates for the design of novel Doc_STm_ inhibitors that will be used in future studies to interrogate the biological role of this TA system in *S.* Typhimurium. As TA systems are still significantly underexplored as antimicrobial targets, further biological characterization with such chemical tools is essential to elucidate their real therapeutic potential.

## Methods

### Biophysical and Biochemical Methods

#### Differential Scanning Fluorimetry

Experiments were performed in a Mx3005P qPCR System (Agilent) collecting fluorescence data with a temperature ramp of 25 to 95 °C. Samples were prepared in a buffer containing 20 mM K_2_HPO_4_ and 50 mM (NH_4_)_2_SO_4_ at pH 8.0. The SYPRO Orange dye (Sigma-Aldrich, 5000× stock in DMSO) was used to monitor protein denaturation and was diluted to a final concentration of 3×. For binding experiments, the final concentration of Doc was 5 μM. Each condition was performed in triplicate (ESI section 4) and the melting curves were plotted using GraphPad Prism 5 (GraphPad Software, U.S.A.). The melting temperatures were obtained by fitting the sigmoidal section of the curves to a Boltzmann sigmoid function.

#### Surface Plasmon Resonance

Experiments were performed in a Biacore S200 (Cytiva) with a Series S sensor chip NTA (Cytiva). The data was analyzed using the Biacore Evaluation Software (Cytiva) and curves were plotted using GraphPad Prism 5 (GraphPad Software, U.S.A.). All experiments were performed at 22 °C with a running buffer containing 10 mM HEPES, 500 mM NaCl, 50 μM EDTA, and 0.005% (v/v) TWEEN 20 at pH 8.0. The surface was conditioned following the standard manufacturer’s guidelines and protein immobilization was performed with a 30–60 s pulse (flow rate of 5 μL/min) of Doc (150 nM) in running buffer. After injection, the absolute response levels typically increased by 200–400 RU. Surface regeneration was performed with a 1 min pulse of 0.5 M imidazole followed by a 1 min pulse of 0.35 M of EDTA at pH 8.0.

Binding to Doc was performed using a multicycle kinetics (MCK) setup for peptides **7**, **10**, and **14**, while the remaining samples were tested using a single-cycle kinetics (SCK) setup. The surface was fully regenerated in between each analyte in both cases. In the MCK experiments, increasing concentrations of each analyte were injected with an 80 s pulse (30 μL/min) and dissociation times varied depending on the time required to fully dissociate the complex. The double-referenced sensorgrams (raw data subtracted from a blank injection and reference surface responses) were analyzed using a kinetic and/or a steady-state 1:1 binding model. In the SCK experiments, typically six concentrations of each analyte were sequentially injected with 80 s pulses (30 μL/min), and dissociation times varied depending on the time required to dissociate at least 5% of the complex. The double-referenced sensorgrams were fitted using a kinetic 1:1 binding model.

At least three independent replicates were measured for each analyte (ESI section 5). When a steady-state fit was applied, the reported *K*_d_ and its uncertainty correspond, respectively, to the average and standard deviations of the *K*_d_ obtained in each measurement. When a kinetic fit was applied, the reported rate constants (*k*_on_ and *k*_off_) and their uncertainties correspond, respectively, to the average and standard deviations of the rate constants obtained in each measurement. In this case, reported *K*_d_ were calculated using the equation below, and the standard deviation of the rate constants was propagated.
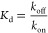


#### Dot Blot Phosphorylation Assay

Recombinant Doc (final concentration: 1 μM) was mixed with recombinant EF-Tu (final concentration: 3 μM) and varying concentrations of recombinant Phd protein or synthetic Phd peptides (final concentrations: 10 μM to 5 nM) in assay buffer (50 mM HEPES, pH 7.5, 25 mM (NH_4_)_2_SO_4_, 2 mM TCEP, 2 mM MgCl_2_ and 1 mM ATP). Samples were prepared to a final volume of 10 μL. EF-Tu (3 μM) in assay buffer was used as negative control (no Doc and Phd peptide/protein), while EF-Tu (3 μM) mixed with Doc (1 μM) in assay buffer was used as positive control (no Doc inhibitor). Samples were incubated for 16 h at RT and subsequently spotted on a nitrocellulose membrane. Phosphorylated EF-Tu was detected by immunodecoration using a rabbit monoclonal antiphosphothreonine antibody (Abcam, ab218195) at 1:2000 dilution (1 h at 4 °C), followed by incubation with a goat antirabbit IgG (H+L) HRP conjugate antibody (Advansta) at 1:10 000 dilution (1 h at RT). Chemiluminescence was developed using the HRP Luminata kit (Merck, WBLUR0100) and captured by an ImageQuant LAS4000 Western blot imaging system (Cytiva).

### Biological Methods

#### Growth Rescue Experiment in *S.* Typhimurium

For the generation of Phd/Doc-expressing *Salmonella* strains, the previously described *S.* Typhimurium (14028) *phd-doc::Km* strain with a knockout of the endogenous Phd–Doc system was used.^10^ A group of pCA24N plasmids was generated by inserting the sequences of full-length wild-type Phd_STm_ antitoxin, Phd_STm_ antitoxin variants containing selected single alanine substitutions or sequences equivalent to selected Phd_STm_ peptides with expression starting at Met52. Each of these plasmids (including an empty pCA24N vector) were cotransformed with a Doc_STm_-expressing pBAD33 plasmid into the aforementioned *Salmonella* strain, enabling coexpression of Doc_STm_ (inducible expression) and different Phd_STm_ constructs (basal leaky expression). The cotransformations above were also performed with an empty pBAD33 plasmid, as a control for the absence of Doc_STm_ expression.

For the growth rescue experiment, overnight (1% tryptone, 0.5% yeast extract, 0.5% NaCl, 1% glucose, 100 μg/mL of carbenicillin, and 34 μg/mL of chloramphenicol) cultures of the generated *S.* Typhimurium strains were diluted to an OD_600_ of approximately 0.006 into fresh M9 minimal medium supplemented with 0.5% or 1% arabinose, 0.4% glycerol, 0.4% casamino acids, 100 μg/mL of carbenicillin, and 34 μg/mL of chloramphenicol. Cell growth at a given time was monitored at OD_600_ with a Genesys 140 Visible Spectrophotometer (Thermo Scientific).

## Supporting Information Available

Additional experimental methods, additional protein expression and purification data, dot blot phosphorylation assay control data, summary of the biophysical binding data, analytical characterization of the synthetic Phd peptides, CD spectroscopy data, homology model, additional DSF data for peptides **24** and **25**, replicate of dot blot phosphorylation assay with selected peptides, additional growth rescue experiment data, literature data for P1 phage Doc, sequence logo for Phd^52–73^, information about the used plasmids and protein constructs (Tables S6–S8), detailed DSF (Tables S9 and S10 and Figures S9–S12) and SPR data (Tables S11–S34 and Figures S13–S36) and links to online data repository (Table S35) (PDF)
