# Supplementary material for: Characterization of the Key Determinants of Phd Antitoxin Mediated Doc Toxin Inactivation in Salmonella
Source: ACS Chem Biol. 2022 Jun 1;17(6):1598–606. doi: 10.1021/acschembio.2c00276 (PMC9207808; doi:10.1021/acschembio.2c00276)
Supplement: Supplementary file 1 — cb2c00276_si_001.pdf [file cb2c00276_si_001.pdf]

Supporting information

Characterisation of the key determinants of Phd antitoxin mediated Doc toxin inactivation in *Salmonella*

Guilherme V. de Castro[1#], Dennis J. Worm[1#], Grzegorz J. Grabe[2], Fiona C. Rowan[1], Lucy Haggerty[1], Ana L. de la Lastra[1], Oana Popescu[1], Sophie Helaine[2] and Anna Barnard[1]*

1) Department of Chemistry, Molecular Sciences Research Hub, Imperial College London, 82 Wood Lane, London W12 0BZ, United Kingdom
2) Department of Microbiology, Harvard Medical School, 4 Blackfan Circle, Boston, MA 02115, USA

Correspondence to: Anna Barnard, Molecular Sciences Research Hub, Imperial College London, 82 Wood Lane, London W12 0BZ, UK. E-Mail: a.barnard@imperial.ac.uk

Contents

1. Additional experimental methods 2
2. Supplementary figures and tables 11
3. Protein constructs, vectors and primers 25
4. Differential scanning fluorimetry data 28
5. Surface plasmon resonance data 33
6. Online data repository 57
7. References 58

1. Additional experimental methods

Peptide analytical data (LC-MS traces, MALDI spectra and CD spectroscopy) and raw data from the biophysical (DSF and SPR) and biological (growth rescue in *S.* Typhimurium) assays can be accessed at the following DOI: 10.14469/hpc/7924. DOIs for individual peptide/experiment folders in the repository can be found in the Table S35.

Peptide synthesis

Materials

9-fluorenylmethoxycarbonyl (Fmoc)-protected amino acids were purchased from AGCT Bioproducts, CEM and Fluorochem. Preloaded Wang resins and Fmoc-Rink Amide MBHA resin were purchased from Advanced Chemtech and Novabiochem, respectively, and *N,N'*-diisopropylcarbodiimide (DIC) was from Fluorochem. Piperidine, acetic anhydride, 2,2′-(Ethylenedioxy)diethanethiol (DODT), triisopropylsilane (TIS), 1,2-ethanedithiol (EDT) and formic acid were from Sigma-Aldrich and Ethyl 2-cyano-2-(hydroxyimino)acetate (Oxyma Pure) was obtained from CEM. Acetonitrile (ACN), dimethylformamide (DMF), dimethyl sulfoxide (DMSO) and diethyl ether were purchased from VWR and dichloromethane (DCM) and trifluoroacetic acid (TFA) were obtained from Fisher Scientific UK.

Solid-phase synthesis, purification and analysis of peptides

Peptides were synthesized by solid phase peptide synthesis (SPPS) using a Liberty Blue automated microwave peptide synthesizer (CEM) and the standard 9-fluorenylmethoxycarbonyl/tert-Butyl (Fmoc/tBu) strategy. Fmoc-Lys(Boc)-Wang resin (50 µmol scale, 0.64 mmol/g, peptides **1-18**, **21** and **25**), Fmoc-Ala-Wang resin (50 µmol scale, 0.9 mmol/g, peptides **19**, **20** and **22**) and Fmoc-Leu-Wang resin (50 µmol scale, 0.83 mmol/g, **23**) were used to obtain peptides as C-terminal acids, while Fmoc-Rink Amide MBHA resin (50 µmol scale, 0.51 mmol/g) was used to obtain peptide **24** as C-terminal amide. For automated SPPS, a 5-fold molar excess of Fmoc-amino acid (250 µmol, 0.2 M solution in DMF) was coupled with 5 eq. Oxyma Pure (250 µmol, 0.5 M solution in DMF) and 10 eq. DIC (500 µmol, 0.5 M solution in DMF) in DMF for 2 min (single coupling) or 2 x 2 min (double coupling) at 90 °C. Cleavage of the N-terminal Fmoc protecting group was accomplished by using 10% (v/v) piperidine, 0.1 M Oxyma Pure in DMF for 1-1.5 min at 90 °C. After completion of automated SPPS, peptides were manually acetylated at the N-terminus with 5% acetic anhydride in DMF for 3 x 15 min.

Cleavage from the resin and simultaneous side chain deprotection was accomplished using a mixture of TFA/TIS/H_2O/DODT (92.5/2.5/2.5/2.5, 3 ml) for 30 min at 40 °C in the CEM Razor rapid peptide cleavage system or a mixture of TFA/TIS/H_2O/EDT (92.5/2.5/2.5/2.5, 3 ml) for 3 hours at room temperature. The cleavage solution was concentrated to around 1 mL under a nitrogen stream and crude peptides were precipitated and washed with ice-cold diethyl ether. The resulting peptide pellets were either dried *in vacuo* or dissolved in ACN/H_2O and subsequently lyophilized. Purification of the peptides was achieved on a Waters LC-MS system with a 2545 quaternary gradient module, 2767 sample manager, system fluidics organizer and 3100 mass detector, or a Shimadzu LC-20AR preparative HPLC system. In the Waters LC-MS system, a preparative reversed-phase Waters XBridge C18 OBD column (100 mm x 19 mm, 5 µm, 130 Å) with a flow rate of 20 mL/min, different linear gradients of eluent B1 (0.1 % (v/v) formic acid in ACN) in eluent A1 (0.1 % (v/v) formic acid in water) and mass-directed fraction collection were used to isolate the desired products. In the Shimadzu HPLC system, a preparative reversed-phase Phenomenex Aeris PEPTIDE XB-C18 column (150 mm x 21 mm, 5 µm, 100 Å) with a flow rate of 20 mL/min, different linear gradients of eluent B2 (0.08 % (v/v) TFA in ACN) in eluent A2 (0.1 % (v/v) TFA in water) and detection at 220 nm were used to isolate desired products. Purity and correct identity of the peptides was determined on the Waters LC-MS using an analytical reversed-phase Waters XBridge C18 column (100 mm × 4.6 mm, 5 µm, 130 Å, 1.2 ml/min) and a linear gradient of 20% to 98% eluent B1 in eluent A1 over 10 min. Chromatograms were recorded at $\lambda = 220$ nm and the masses of the products were detected in the range from 400-2000 m/z. The identity of the pure peptides was additionally confirmed by MALDI-ToF mass spectrometry (Micromass, Waters). The observed masses were in agreement with the calculated masses and a purity of > 93 % could be obtained for all compounds by LC-MS analysis (ESI Tables S2 and S3).

Molecular Cloning and Protein Constructs

The full-length *Salmonella typhimurium* sequences of Doc (Uniprot: E8XF70), Phd (Uniprot: E8XF71) and EF-Tu (Uniprot: P0A1H5) were used to generate all the plasmids described. For generation of the Doc-His protein construct, the sequence of Doc was PCR-amplified from *S.* Typhimurium genomic DNA and cloned into pET28 plasmid using NcoI (5' end) and XhoI (3' end) restriction sites and standard DNA ligation. For Doc expression in the growth rescue experiments, the PCR-amplified sequence of Doc was cloned into a modified pBAD33 vector with flipped resistance cassette (ampicillin instead of chloramphenicol)[1] using SacI (5' end) and XbaI (3' end) restriction sites.

The pET28-FLAG vector was generated by cloning the sequence for the FLAG tag into pET28 upstream of the multiple cloning site. The sequence of Phd^{1-73} protein was PCR-amplified from *S.* Typhimurium genomic DNA and cloned into pET28-FLAG vector using NheI (5' end) and BamHI (3' end) restriction sites. For expression of Phd^{1-73} protein variants and Phd^{52-73} peptides in the growth rescue experiments, respective Phd sequences were cloned into pCA24N vector[2] using MfeI (5' end) and NotI (3' end) restriction sites. For the co-expression of EF-Tu with Doc, the EF-Tu sequence was PCR-amplified from *S.* Typhimurium genomic DNA and cloned into modified pBAD33 vector using SacI (5' end) and HindIII (3' end) restriction sites and standard DNA ligation. A ribosomal binding site (RBS) was subsequently inserted into the pBAD33 plasmid upstream of the cloned EF-Tu sequence using 5' phosphorylated primers. For generation of the EF-Tu-His protein construct, the PCR-amplified EF-Tu sequence was cloned into the pET24a vector using NdeI (5' end) and XhoI (3' end) restriction sites. Generation of the EF-Tu^{T383A} mutant in both pBAD33 and pET24a plasmids was accomplished by PCR amplification of the EF-Tu insert using the respective forward cloning primer and a designated mutagenesis reverse primer containing the T383A mutation, followed by reinsertion of the amplified sequence into empty, modified pBAD33 plasmid and pET24a plasmid using SacI/HindIII (pBAD33) and NdeI/XhoI (pET24a) restriction sites, respectively. EF-Tu^{T383V} mutant in both pBAD33 and pET24a plasmids was accomplished by amplification into a linear vector using non-overlapping primers (just the forward primer containing the mutation). The linear strand was phosphorylated at the 5' ends and ligated using standard protocols, followed by a transformation where successfully mutated vector was selected after DNA sequencing of colonies. Details of each protein construct and plasmid, as well as the used primer sequences, can be found in the ESI file section 3.

Protein Expression, Purification and Characterisation

Preparation of BL21-AI *E. coli* cells for co-expression of EF-Tu and Doc proteins

A microcentrifuge tube containing 50 µL of competent BL21-AI™ One Shot™ chemically competent *E. coli* cells (ThermoFisher Scientific) was kept in ice for 20 minutes, followed by the addition of 1.5 µL of the desired pBAD plasmid for expression of EF-Tu (wild-type, T383A or T383V) at 50–100 ng/µL. After 10 minutes in ice, the cells were incubated in a 42 °C water bath for 45 seconds, then returned to ice for 5 minutes. The microcentrifuge tube was removed from ice and 450 µL of SOC medium (ThermoFisher Scientific) were added, followed by a 45-60 minutes incubation at 37 °C. After this period, 100 µL of this cell suspension was spread over a Luria-Bertani (LB) agar plate containing 100 µg/mL of ampicillin (Amp) and left to

grow overnight at 37 °C. Successfully transformed cells were then selected from the observed colonies.

Before introducing the plasmid containing the Doc protein sequence, these cells were made chemically competent again using the following procedure: colonies from the previous transformation were used to inoculate 10 mL of LB medium (100 µg/mL of Amp). After shaking overnight at 37 °C the resulting high-density culture was transferred to a 2 L conical flask containing 1 L of LB medium (100 µg/mL of Amp). The flask was left shaking at 37 °C until the optical density of the bacterial culture at 600 nm (OD_{600}) reached 0.35–0.40. At this stage the cells were chilled on ice for 30 minutes and harvested by centrifugation at 3000 x *g* (30 minutes at 4 °C). The pellet was gently resuspended in 100 mL of an ice-cold solution of 0.1 M $MgCl_2$ and the cells were harvested by centrifugation at 2000 x *g* (15 minutes at 4 °C). The pellet was gently resuspended in 200 mL an ice-cold solution of 0.1 M $CaCl_2$ and kept in ice for 20 minutes. After this period the cells were harvested by centrifugation at 2000 x *g* (15 minutes at 4 °C) and the pellet was gently resuspended in 50 mL of an ice-cold solution of 15% glycerol and 0.085 M $CaCl_2$. The cells were harvested by centrifugation at 1000 x *g* (15 minutes at 4 °C) and the pellet was gently resuspended in 50 mL of an ice-cold solution of 15% glycerol and 0.085 M $CaCl_2$. This suspension of chemically competent cells was split in sterile 0.5 mL microcentrifuge tubes as 50 µL aliquots, flash frozen with liquid nitrogen and stored at -80 °C.

Growth curves in *E. coli*

Chemically competent BL21-AI *E. coli* cells were transformed with the following set of plasmids using standard protocols, generating in total four transformants: 1) pET28 Doc; 2) pET28 Doc + pBAD EF-Tu; 3) pET28 Doc + pBAD EF-Tu^{T383A}; 4) pET28 Doc + pBAD EF-Tu^{T383V}. Successfully transformed cells were used to inoculate 15 mL of LB medium with suitable antibiotics (ESI Table S6) in a 50 mL conical flask and left shaking overnight at 37 °C. In the following morning, for each transformant, three 50 mL conical flasks (Labelled A, B and C) containing 25 mL of LB (with suitable antibiotics) were inoculated with 0.25 mL of the overnight culture. In all cases flasks A and B were supplied with 0.1% (w/v) of L-arabinose and all flasks were left shaking at 37 °C. Growth was monitored and once the bacterial cultures in flasks A reached OD_{600} of 0.6–0.8, isopropyl-β-D-thiogalactoside (IPTG) was added to a final concentration of 0.5 mM. Aliquots at this time point were collected and lysed for SDS-PAGE following standard protocols. Growth was monitored for further ~7 h, where a final aliquot was collected for SDS-PAGE. The same experiment was repeated replacing LB medium with

terrific broth (TB) medium, and due to the higher density TB cultures, growth was monitored for a further 20 h after IPTG induction (ESI Figure S1).

Protein purification – General conditions

All the procedures were performed at 4 °C. The following buffers were prepared for the immobilised metal ion affinity chromatography (IMAC), ion-exchange chromatography (IEC) and size-exclusion chromatography (SEC) steps:

- IMAC binding buffer: 0.05 M K_2HPO_4, 0.3 M NaCl, 10% glycerol, pH 8.0.
- IMAC elution buffer: 0.05 M K_2HPO_4, 0.3 M NaCl, 0.5 M imidazole, 10% glycerol, pH 8.0.
- Doc IEC binding buffer: 0.02 M K_2HPO_4, 10% glycerol, pH 8.0.
- Doc IEC elution buffer: 0.02 M K_2HPO_4, 1 M NaCl, 10% glycerol, pH 8.0.
- EF-Tu IEC binding buffer: 0.02 M K_2HPO_4, 2 mM tris(2-carboxyethyl)phosphine (TCEP), 5% glycerol, pH 8.0.
- EF-Tu IEC elution buffer: 0.02 M K_2HPO_4, 1 M NaCl, 2 mM TCEP, 5% glycerol, pH 8.0.
- Doc SEC buffer: 0.02 M K_2HPO_4, 0.025 M $(NH_4)_2SO_4$, 10% glycerol, pH 8.0.
- Phd SEC buffer: 0.025 M K_2HPO_4, 0.15 M NaCl, 5% glycerol, pH 8.0.
- EF-Tu SEC buffer: 0.02 M tris(hydroxymethyl)aminomethane (TRIS), 0.15 M NaCl, 1 mM TCEP, 5% glycerol, pH 8.0.

Expression and purification of the Doc toxin

Since a higher percentage of monomeric EF-Tu$_{STm}$ was observed for T383V when compared to T383A, suggesting higher solubility, co-expression with EF-Tu$_{STm}$ T383V was preferred for the large-scale production of Doc (ESI Figure S1). Chemically competent BL21-AI *E. coli* cells containing the EF-Tu^{T383V} pBAD plasmid (50 µL aliquot) were transformed with the Doc pET28 plasmid using the same transformation protocol described for the pBAD plasmid. After the outgrowth period in SOC medium, the whole cell suspension was transferred to a 2 L baffled conical flask containing 0.5 L of terrific broth (TB) medium supplemented with 0.1% (w/v) of L-arabinose, 50 µg/mL of kanamycin (Kan) and 100 µg/mL of Amp. Plating and overnight culture steps were skipped to minimise the time cells carried the toxic Doc plasmid, which

significantly improved reproducibility. This flask was left in an incubator overnight at 30 °C shaking at 250 rpm. The temperature was then increased to 37 °C and the flask was left shaking at same speed. Typically, after 1.5–2.5 h the bacterial culture reached an OD_{600} of 1.0–1.5. At this stage the temperature was lowered to 18 °C and IPTG was added to a final concentration of 0.5 mM. The cells were left shaking at 250 rpm overnight and were harvested by centrifugation at 3000 x *g* (30 minutes at 4 °C).

The cell pellets resuspended in ice-cold IMAC binding buffer (5 mL per gram of cell paste), followed by the addition of cOmplete™ mini EDTA-free protease inhibitor cocktail (Roche, 1 tablet per 50 mL of lysate), Benzonase® Nuclease 250 U/µL (Sigma-Aldrich, 1 µL per 100 mL of lysate) and $MgCl_2$ to a final concentration of 5 mM. This suspension was left shaking for 30 minutes and the cells were lysed with two passages at 25 kpsi and 5 °C in a CF1 Cell Disrupter (Constant Systems Ltd.) coupled with a ThermoFlex1400 (ThermoFisher Scientific) cooling unit. The lysate was clarified by centrifugation at 18,000 rpm (45 minutes at 4 °C) in a Sorvall LYNX 4000 superspeed centrifuge (ThermoFisher Scientific) with a Fiberlite™ F21-8 x 50y fixed-angle rotor attached (ThermoFisher Scientific).

Using an ÄKTA pure (Cytiva) protein purification system, the supernatant was loaded into a 5 mL HiTrap TALON® crude column (Cytiva) previously equilibrated with the IMAC binding buffer. Two or three columns were attached in tandem when lysate volumes exceeded 100 mL to avoid Doc precipitation after elution (poor solubility when too concentrated). After the sample application was completed, the column was washed with 3–5 column volumes (CV) of 0.5% (v/v) of IMAC elution buffer in IMAC binding buffer. Bound proteins were eluted with a 0.5–100% (v/v) gradient of IMAC elution buffer in IMAC binding buffer over 8 CV, with Doc typically being eluted at 25–75% (v/v) of the gradient as a broad peak with low absorbance at 280 nm (A_{280}).

Doc containing fractions (verified by SDS-PAGE) were combined, transferred to a 3.5K MWCO SnakeSkin™ Dialysis Tubing (ThermoFisher Scientific) and dialysed overnight in a beaker containing 2 L of Doc IEC binding buffer. The sample was filtered (0.2 µm) and loaded in a 5 mL HiTrap Q HP anion exchange column (Cytiva) previously equilibrated with the Doc IEC binding buffer. After the sample loading, the column was washed with 10 CV of Doc IEC binding buffer and bound proteins were eluted with a 0–75% gradient of Doc IEC elution buffer in Doc IEC binding buffer over 20 CV. In these conditions, Doc does not bind to the column and is collected with high purity during the sample application and column wash steps, while most of the impurities were collected in the elution step. Attempts to increase the pH to retain

Doc in the column for the elution step were not preferred as those led to protein with poor activity in the biophysical assays.

Doc containing fractions were concentrated using a 3000 MWCO PES Vivaspin® 20 centrifugal concentrator (Sartorius) to approximately 25–50 µM, filtered (0.2 µm) and loaded in a HiLoad 16/600 Superdex 75pg column (Cytiva) previously equilibrated with Doc SEC buffer. After an isocratic elution of Doc SEC buffer over 1 CV, Doc containing fractions were eluted with high purity in between retention volumes of 75–90 mL. Using the manufacturer's calibration curves, the eluted sample corresponded to a molecular weight of approximately 15 kDa, showing that the Doc protein was obtained in a monomeric form (expected 14.8 kDa). Fractions were combined and concentrated to approximately 50–75 µM (attempts to concentrate the protein further resulted in precipitation), flash frozen in liquid nitrogen and stored at -80 °C. The yield of the purification was of approximately 3 mg of Doc per litre of TB medium.

Expression and purification of the Phd antitoxin

BL21(DE3) *E. coli* cells were transformed with the pET-FLAG Phd plasmid using standard protocols. Successfully transformed cells were used to inoculate 15 mL of TB medium (Kan 50 µg/mL) in a 50 mL conical flask. The flask was left shaking overnight at 37 °C and in the following morning the contents were split into two 2 L conical flasks containing 0.75 L of LB media (Kan 50 µg/mL). The flasks were left shaking at 37 °C and typically after 2–3 h the bacterial culture reached an OD_{600} of 1.0–1.5. At this stage the temperature was lowered to 18 °C and IPTG was added to a final concentration of 0.5 mM. The cells were left shaking overnight at this temperature and were harvested by centrifugation at 3000 x *g* (30 minutes at 4 °C).

The lysis and IMAC purification steps were performed using the same procedures, reagents and buffers as described for Doc. The Phd protein eluted at 10–65% (v/v) of the gradient as a broad peak and fractions were combined. To remove the hexahistidine (His_6) tag, 250 µL of 10 kU/mL Thrombin from bovine plasma (Sigma-Aldrich, diluted in water from the lyophilised powder) were added to the pooled fractions. This mixture was transferred to a 3.5K MWCO SnakeSkin™ Dialysis Tubing (ThermoFisher Scientific) and dialysed overnight in a beaker containing 2 L of IMAC binding buffer. After this period the sample was submitted to the same IMAC purification step. Due to the cleavage of the His_6 tag, the Phd protein was collected during the sample application and column wash steps, while most of the impurities were collected in the elution step.

Phd containing fractions were concentrated using a 3000 MWCO PES Vivaspin® 20 centrifugal concentrator (Sartorius) to approximately 2.0–5.0 mL, filtered (0.2 µm) and loaded in a HiLoad 16/600 Superdex 75pg column previously equilibrated with Phd SEC buffer. After an isocratic elution of Phd SEC buffer over 1 CV, Phd containing fractions were eluted with high purity in between retention volumes of 60–75 mL. Using the manufacturer's calibration curves, the eluted sample corresponded to a molecular weight of approximately 30 kDa (for an ordered globular protein), showing that the Phd protein (10 kDa) was likely obtained as the expected[3] flexible dimer (longer migration in the gel filtration column). Fractions were combined and concentrated to approximately 275 µM, flash frozen in liquid nitrogen and stored at -80 °C. The yield of the purification was of approximately 25 mg of Phd per litre of TB medium.

Expression and purification of EF-Tu and EF-Tu^{T383V}

BL21(DE3) *E. coli* cells were transformed with the pET24a plasmid (wild-type or T383V) using standard protocols. Successfully transformed cells were used to inoculate 15 mL of TB medium (Kan 50 µg/mL) in a 50 mL conical flask. The flask was left shaking overnight at 37 °C and in the following morning the contents were split into two 2 L conical flasks containing 0.75 L (Kan 50 µg/mL). The flasks were left shaking at 37 °C and typically after 2–3 h the bacterial culture reached an OD_{600} of 1.0–1.5. At this stage the temperature was lowered to 18 °C and IPTG was added to a final concentration of 0.5 mM. The cells were left shaking overnight at this temperature and were harvested by centrifugation at 3000 x *g* (30 minutes at 4 °C).

The lysis and TALON purification steps were performed using the same procedures, reagents and buffers described for Doc. The protein eluted at 10–65% (v/v) of the gradient as a broad peak. Fractions were combined, transferred to a 3.5K MWCO SnakeSkin™ Dialysis Tubing (ThermoFisher Scientific) and dialysed overnight in a beaker containing 2 L of EF-Tu IEC buffer.

The sample was filtered (0.2 µm) and loaded in a double 5 mL HiTrap Q HP anion exchange column (Cytiva) previously equilibrated with the EF-Tu IEC binding buffer. After the sample loading, the column was washed with 5 CV of EF-Tu IEC binding buffer and bound proteins were eluted with a 0–75% gradient of EF-Tu IEC elution buffer in EF-Tu IEC binding buffer over 10 CV. EF-Tu containing fractions were recovered in the elution phase at 35–50% of the gradient, combined and concentrated using a 3000 MWCO PES Vivaspin® 20 centrifugal concentrator (Sartorius) to approximately 2.0–5.0 mL.

The sample was filtered (0.2 µm) and loaded in a HiLoad 16/600 Superdex 75pg column previously equilibrated with EF-Tu SEC buffer. After an isocratic elution in the same buffer over 1 CV, EF-Tu containing fractions were eluted with high purity in between retention volumes of 55–65 mL. Using the manufacturer's calibration curves, the eluted sample corresponded to a molecular weight of approximately 45 kDa, showing that the EF-Tu proteins were obtained in a monomeric form (expected 44.3 kDa). Fractions were combined and concentrated to approximately 260 µM, flash frozen in liquid nitrogen and stored at -80 °C. For the EF-Tu wild-type and T383V proteins, the approximate yield of per litre of TB medium was, respectively, 16 mg and 5 mg.

Biophysical methods

Circular dichroism

Lyophilized peptides were dissolved in a 20 mM potassium phosphate buffer (pH 7.4) or in 30% (v/v) trifluoroethanol (TFE) in 20 mM potassium phosphate buffer to concentrations of 25-50 µM. Peptide solutions (300 µL) were transferred into a Hellma QS quartz cuvette (1 mm) and CD spectra were recorded with a Chirascan V100 CD spectrometer (Applied Photophysics) in the 190-260 nm far UV range at 25 ºC with 1 nm band width, 1 nm step size, 1 sec per step and five scans per measurement. In addition, a background measurement of buffer or 30% TFE in buffer was performed. Scans for each measurement were averaged, the averaged spectra were smoothened using Savitsky-Golay smoothing (window size of 5) and the buffer spectra were subtracted from the respective peptide spectra. CD values in mdeg were converted to mean residue ellipticity [θ] (deg. · cm^2 · $dmol^{-1}$) and the peptide spectra were plotted using GraphPad Prism 5.

$$[\theta] = \frac{mdeg\ \times mean\ residue\ weight\ in\ g/mol}{path\ length\ in\ mm\ \times concentration\ in\ mg/mL}$$

Computational methods

Homology model

Phd-Doc toxin-antitoxin complex model was generated using SWISS-MODEL automated protein structure homology-modelling server (PMID: 29788355) available at https://swissmodel.expasy.org/ using Doc (PDB:3K33) and Phd (PDB:3K33) as template structures.

2. Supplementary figures and tables

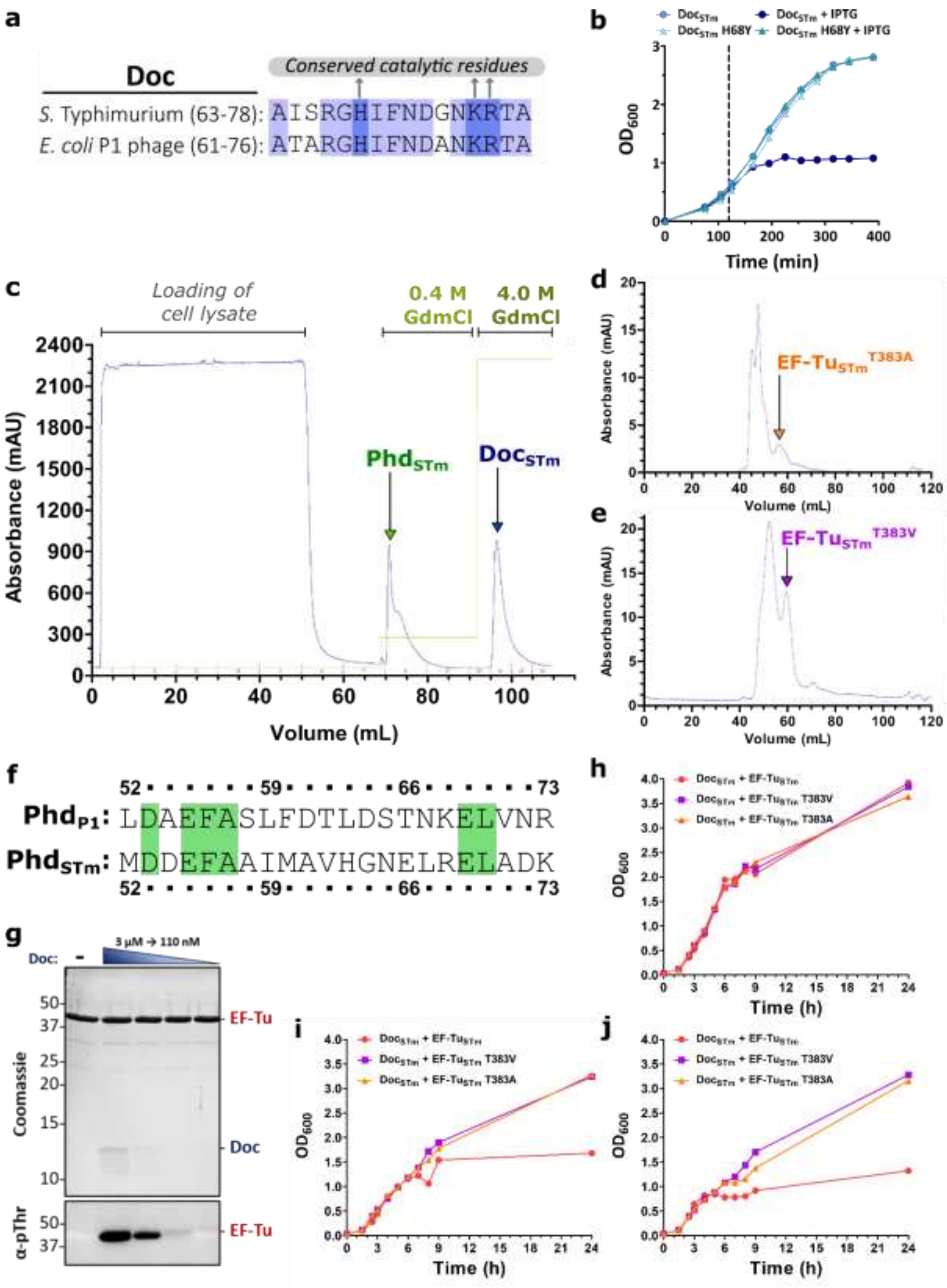

Figure S1. Supplementary protein expression and purification data. (a) Sequence alignment of the Doc_{STm} and Doc_{P1}, with conserved residues highlighted in light and dark blue. **(b)** Growth curves of E. coli expressing Doc_{STm} wild-type and H68Y in the absence or presence of IPTG. **(c)** Denaturation of the Doc_{STm}-Phd_{STm} complex by increasing concentrations of guanidinium chloride (GdmCl) directly on a 1 mL HisTrap FF column (Cytiva). The proteins were co-expressed in BL21 pLysS cells carrying a pET-DUET vector encoding for the full length Doc_{STm} (C-terminal hexahistidine tag) and Phd_{STm}. Size-exclusion purification of EF-Tu^{T383A} **(d)** and EF-Tu^{T383V} **(e)** proteins (both C-terminal hexahistidine tagged) from a purification trial, where a higher fraction of monomeric EF-Tu^{T383V} was observed. In both cases the expression lysate was initially purified in a 1 mL HisTrap FF column (Cytiva) and the imidizole-eluted fractions were combined, concentrated and loaded onto a Superdex 75 16-600 (Cytiva). **(f)** Sequence alignment of the neutralisation domain of Phd_{P1} and Phd_{STm} (conserved residues in green). **(g)** Coomassie and western blot (α-phosphothreonine) analysis of phosphorylation reactions of EF-Tu_{STm} (fixed at 2.25 µM) in the presence of 1 mM ATP and refolded Doc_{STm}. Growth curves of the co-expression of Doc with EF-Tu wild-type (red), T383A (orange) and T383V (violet) in terrific broth (TB) medium supplied with: **(h)** No IPTG and no L-Arabinose; **(i)** No IPTG and 0.1% (w/v) Arabinose; **(j)** 0.5 mM IPTG and 0.1% (w/v) L-Arabinose.

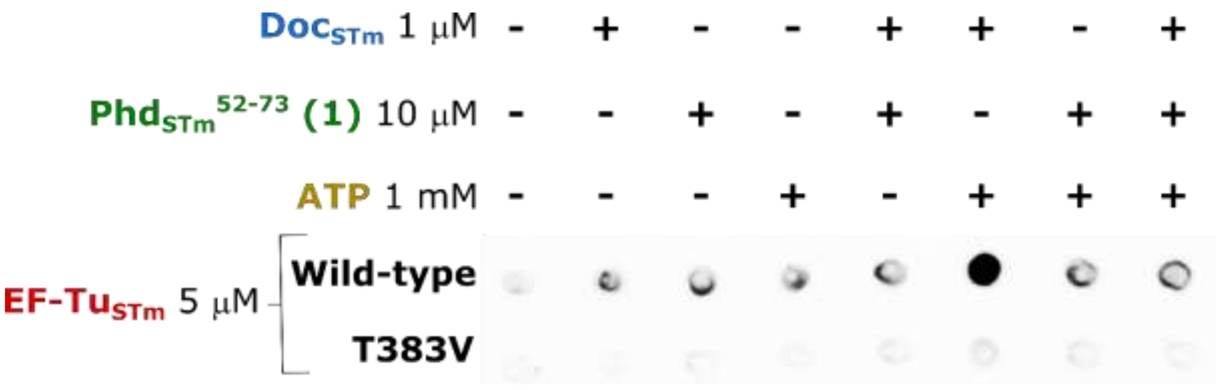

Figure S2. EF-Tu$_{STm}$ phosphorylation is site-specific and ATP-dependent. Dot blot phosphorylation assay performed with EF-Tu$_{STm}$ (wild-type or T383V variant) in the presence of multiple combinations of Doc$_{STm}$, peptide **1** and ATP.

Table S1. Summary of the melting temperature shift (ΔT_m), kinetic contants (k_{on}, k_{off} and half-life, where applicable) and dissociation constants (K_D) of Phd_{STm}^{1-73} and peptides **1-25** (analytes) binding to Doc_{STm}. All SPR datasets were fitted to a 1:1 kinetic or steady-state binding model.

**Analyte**	**ΔT_m (°C)** [a]	**$k_{on} \times 10^6$ (M^{-1} s^{-1})**	**$k_{off} \times 10^{-6}$ (s^{-1})**	**K_D (pM)**	**Half-life (h)** [b]
Phd_{STm}^{1-73}	31.3 ± 0.9	0.473 ± 0.109	29 ± 6	61 ± 19	6.7 ± 1.4
**1** (Phd_{STm}^{52-73})	28.8 ± 0.9	1.250 ± 0.259	91 ± 11	73 ± 18	2.1 ± 0.3
**2** (Phd_{STm}^{52-73}-M52A)	28.0 ± 1.0	1.136 ± 0.140	59 ± 11	52 ± 12	3.3 ± 0.6
**3** (Phd_{STm}^{52-73}-D53A)	25.6 ± 0.9	5.073 ± 0.369	65 ± 18	13 ± 4	2.9 ± 0.8
**4** (Phd_{STm}^{52-73}-D54A)	26.2 ± 0.9	4.733 ± 0.155	62 ± 17	13 ± 4	3.1 ± 0.8
**5** (Phd_{STm}^{52-73}-E55A)	23.4 ± 0.9	1.542 ± 0.147	75 ± 5	49 ± 6	2.6 ± 0.2
**6** (Phd_{STm}^{52-73}-F56A)	21.1 ± 1.0	0.989 ± 0.434	843 ± 231	852 ± 441	0.23 ± 0.06
**7** (Phd_{STm}^{52-73}-I59A)	15.5 ± 1.5	N.D. [c]	N.D. [c]	19950 ± 5176	N.D. [c]
**8** (Phd_{STm}^{52-73}-M60A)	20.8 ± 1.0	2.221 ± 1.307	1223 ± 504	553 ± 397	0.16 ± 0.6
**9** (Phd_{STm}^{52-73}-V62A)	24.6 ± 1.2	2.266 ± 0.942	388 ± 57	171 ± 76	0.50 ± 0.07
**10** (Phd_{STm}^{52-73}-H63A)	20.9 ± 1.0	N.D. [c]	N.D. [c]	21750 ± 15810	N.D. [c]
**11** (Phd_{STm}^{52-73}-G64A)	26.9 ± 1.1	2.350 ± 1.103	197 ± 49	84 ± 45	1.0 ± 0.2
**12** (Phd_{STm}^{52-73}-N65A)	28.2 ± 1.2	1.396 ± 0.154	203 ± 103	145 ± 76	1.0 ± 0.5
**13** (Phd_{STm}^{52-73}-E66A)	27.7 ± 0.9	2.705 ± 1.041	112 ± 38	42 ± 21	1.7 ± 0.6
**14** (Phd_{STm}^{52-73}-L67A)	14.3 ± 0.9	N.D. [c]	N.D. [c]	21430 ± 5247	N.D. [c]
**15** (Phd_{STm}^{52-73}-R68A)	27.7 ± 0.9	1.602 ± 0.332	185 ± 52	115 ± 40	1.0 ± 0.3
**16** (Phd_{STm}^{52-73}-E69A)	30.7 ± 0.9	2.101 ± 0.333	136 ± 41	65 ± 22	1.4 ± 0.4
**17** (Phd_{STm}^{52-73}-L70A)	15.2 ± 1.0	3.837 ± 1.398	5967 ± 404	1550 ± 580	0.032 ± 0.002
**18** (Phd_{STm}^{52-73}-D72A)	28.0 ± 0.9	2.508 ± 0.964	84 ± 33	33 ± 18	2.3 ± 0.9
**19** (Phd_{STm}^{52-73}-K73A)	25.2 ± 1.1	1.441 ± 0.462	126 ± 11	87 ± 29	1.5 ± 0.1
**20** (Phd_{STm}^{52-71})	25.6 ± 0.9	0.917 ± 0.023	393 ± 18	428 ± 23	0.49 ± 0.02
**21** (Phd_{STm}^{55-73})	19.3 ± 0.9	1.252 ± 0.080	496 ± 58	396 ± 52	0.39 ± 0.05
**22** (Phd_{STm}^{55-71})	16.3 ± 0.9	1.449 ± 0.085	503 ± 16	347 ± 23	0.38 ± 0.01
**23** (Phd_{STm}^{56-70})	7.9 ± 1.0	1.413 ± 0.136	2794 ± 42	1978 ± 193	0.069 ± 0.001
**24** (Phd_{STm}^{52-63})	3.1 ± 0.9	N.D. [d]	N.D. [d]	N.D. [d]	N.D. [d]
**25** (Phd_{STm}^{65-73})	1.3 ± 0.9	N.D. [d]	N.D. [d]	N.D. [d]	N.D. [d]

N.D.: Not determined. [a] Subtraction of the T_m of Doc_{STm} free in solution (42.9 ± 0.9 °C) from the measured T_m in the presence of each analyte. [b] Half-life, defined as $t_{1/2} = \ln 2 / k_{off}$.[4] [c] Steady-state fit, kinetic constants not obtained. [d] Affinity was too weak to be determined by SPR. No significant or concentration dependent responses.

Table S2. Analysis of pure Phd peptides by analytical LC-MS. A reversed-phase Waters XBridge C18 column (100 mm x 4.6 mm, 5 µm, 130 Å) with a flow rate of 1.2 mL/min and a linear gradient of 20% to 98% eluent B1 (0.1% formic acid in acetonitrile) in eluent A1 (0.1% formic acid in water) over 10 min was used.

**Compound**	**Sequence**	**t_R [min]**	**Purity**
**1** (Phd_{STm}^{52-73})	Ac-WMDDEFAAIMAVHGNELRELADK-OH	7.95	99%
**2** (Phd_{STm}^{52-73}-M52A)	Ac-W**A**DDEFAAIMAVHGNELRELADK-OH	7.91	99%
**3** (Phd_{STm}^{52-73}-D53A)	Ac-WM**A**DEFAAIMAVHGNELRELADK-OH	8.29	99%
**4** (Phd_{STm}^{52-73}-D54A)	Ac-WMD**A**EFAAIMAVHGNELRELADK-OH	8.55	98%
**5** (Phd_{STm}^{52-73}-E55A)	Ac-WMDD**A**FAAIMAVHGNELRELADK-OH	8.35	97%
**6** (Phd_{STm}^{52-73}-F56A)	Ac-WMDDE**A**AAIMAVHGNELRELADK-OH	7.56	99%
**7** (Phd_{STm}^{52-73}-I59A)	Ac-WMDDEFAA**A**MAVHGNELRELADK-OH	8.18	99%
**8** (Phd_{STm}^{52-73}-M60A)	Ac-WMDDEFAAI**A**AVHGNELRELADK-OH	7.82	99%
**9** (Phd_{STm}^{52-73}-V62A)	Ac-WMDDEFAAIMA**A**HGNELRELADK-OH	7.71	95%
**10** (Phd_{STm}^{52-73}-H63A)	Ac-WMDDEFAAIMAV**A**GNELRELADK-OH	9.81	96%
**11** (Phd_{STm}^{52-73}-G64A)	Ac-WMDDEFAAIMAVH**A**NELRELADK-OH	8.09	97%
**12** (Phd_{STm}^{52-73}-N65A)	Ac-WMDDEFAAIMAVHG**A**ELRELADK-OH	8.10	96%
**13** (Phd_{STm}^{52-73}-E66A)	Ac-WMDDEFAAIMAVHGN**A**LRELADK-OH	8.07	97%
**14** (Phd_{STm}^{52-73}-L67A)	Ac-WMDDEFAAIMAVHGNE**A**RELADK-OH	7.70	99%
**15** (Phd_{STm}^{52-73}-R68A)	Ac-WMDDEFAAIMAVHGNEL**A**ELADK-OH	8.55	97%
**16** (Phd_{STm}^{52-73}-E69A)	Ac-WMDDEFAAIMAVHGNELR**A**LADK-OH	8.00	94%
**17** (Phd_{STm}^{52-73}-L70A)	Ac-WMDDEFAAIMAVHGNELRE**A**ADK-OH	7.75	97%
**18** (Phd_{STm}^{52-73}-D72A)	Ac-WMDDEFAAIMAVHGNELRELA**A**K-OH	7.98	98%
**19** (Phd_{STm}^{52-73}-K73A)	Ac-WMDDEFAAIMAVHGNELRELAD**A**-OH	8.51	93%
**20** (Phd_{STm}^{52-71})	Ac-WMDDEFAAIMAVHGNELRELA-OH	8.98	99%
**21** (Phd_{STm}^{55-73})	Ac-EFAAIMAVHGNELRELADK-OH	6.99	98%
**22** (Phd_{STm}^{55-71})	Ac-EFAAIMAVHGNELRELA-OH	7.97	99%
**23** (Phd_{STm}^{56-70})	Ac-FAAIMAVHGNELREL-OH	8.06	99%
**24** (Phd_{STm}^{52-63})	Ac-WMDDEFAAIMAVH-NH_2	8.77	99%
**25** (Phd_{STm}^{65-73})	Ac-WNELRELADK-OH	7.26	99%

t_R: Retention time.

Table S3. Analysis of pure Phd peptides by LC-MS (ESI$^+$) and MALDI-ToF-MS.

**Compound**	**M_{exact} (calc) [Da]**	**M_W (calc) [Da]**	**ESI$^+$ (LC-MS)**	**MALDI-ToF**
			**m/z (exp)**	**M_{exact} (exp) [M+H]+**
**1** ($Phd_{STm}^{52\text{-}73}$)	2702.3	2704.0	1352.9 (m/2), 902.4 (m/3)	2703.4
**2** ($Phd_{STm}^{52\text{-}73}$-M52A)	2642.2	2643.9	1323.0 (m/2), 882.4 (m/3)	2643.3
**3** ($Phd_{STm}^{52\text{-}73}$-D53A)	2658.3	2660.0	1331.0 (m/2), 887.8 (m/3)	2659.3
**4** ($Phd_{STm}^{52\text{-}73}$-D54A)	2658.3	2660.0	1330.9 (m/2), 887.7 (m/3)	2659.2
**5** ($Phd_{STm}^{52\text{-}73}$-E55A)	2644.2	2646.0	1324.5 (m/2), 883.2 (m/3)	2645.2
**6** ($Phd_{STm}^{52\text{-}73}$-F56A)	2626.2	2627.9	1315.0 (m/2), 877.1 (m/3)	2627.1
**7** ($Phd_{STm}^{52\text{-}73}$-I59A)	2660.2	2661.9	1331.8 (m/2), 888.2 (m/3)	2660.9
**8** ($Phd_{STm}^{52\text{-}73}$-M60A)	2642.2	2643.9	1322.9 (m/2), 882.3 (m/3)	2643.3
**9** ($Phd_{STm}^{52\text{-}73}$-V62A)	2674.2	2676.0	1339.0 (m/2), 893.1 (m/3)	2675.4
**10** ($Phd_{STm}^{52\text{-}73}$-H63A)	2636.2	2638.0	1319.8 (m/2)	2637.2
**11** ($Phd_{STm}^{52\text{-}73}$-G64A)	2716.3	2718.0	1360.0 (m/2), 907.1 (m/3)	2717.1
**12** ($Phd_{STm}^{52\text{-}73}$-N65A)	2659.2	2661.0	1331.5 (m/2), 888.0 (m/3)	2660.1
**13** ($Phd_{STm}^{52\text{-}73}$-E66A)	2644.2	2646.0	1323.9 (m/2), 883.1 (m/3)	2645.5
**14** ($Phd_{STm}^{52\text{-}73}$-L67A)	2660.2	2662.0	1331.9 (m/2), 888.1 (m/3)	2661.0
**15** ($Phd_{STm}^{52\text{-}73}$-R68A)	2617.2	2618.9	1310.4 (m/2)	2618.0
**16** ($Phd_{STm}^{52\text{-}73}$-E69A)	2644.2	2646.0	1323.9 (m/2), 883.1 (m/3)	2645.2
**17** ($Phd_{STm}^{52\text{-}73}$-L70A)	2660.2	2662.0	1331.9 (m/2), 888.4 (m/3)	2661.2
**18** ($Phd_{STm}^{52\text{-}73}$-D72A)	2658.3	2660.0	1331.1 (m/2), 887.8 (m/3)	2659.4
**19** ($Phd_{STm}^{52\text{-}73}$-K73A)	2645.2	2646.9	1324.5 (m/2)	2646.3
**20** ($Phd_{STm}^{52\text{-}71}$)	2459.1	2460.8	1231.3 (m/2), 821.4 (m/3)	2460.2
**21** ($Phd_{STm}^{55\text{-}73}$)	2155.1	2156.5	1079.3 (m/2), 720.0 (m/3)	2155.9
**22** ($Phd_{STm}^{55\text{-}71}$)	1912.0	1913.2	957.6 (m/2)	1912.8
**23** ($Phd_{STm}^{56\text{-}70}$)	1711.9	1713.0	857.5 (m/2)	1712.6
**24** ($Phd_{STm}^{52\text{-}63}$)	1575.7	1576.8	1576.7 (m/1)	1576.2
**25** ($Phd_{STm}^{65\text{-}73}$)	1314.7	1315.4	1315.6 (m/1), 658.4 (m/2)	1315.1

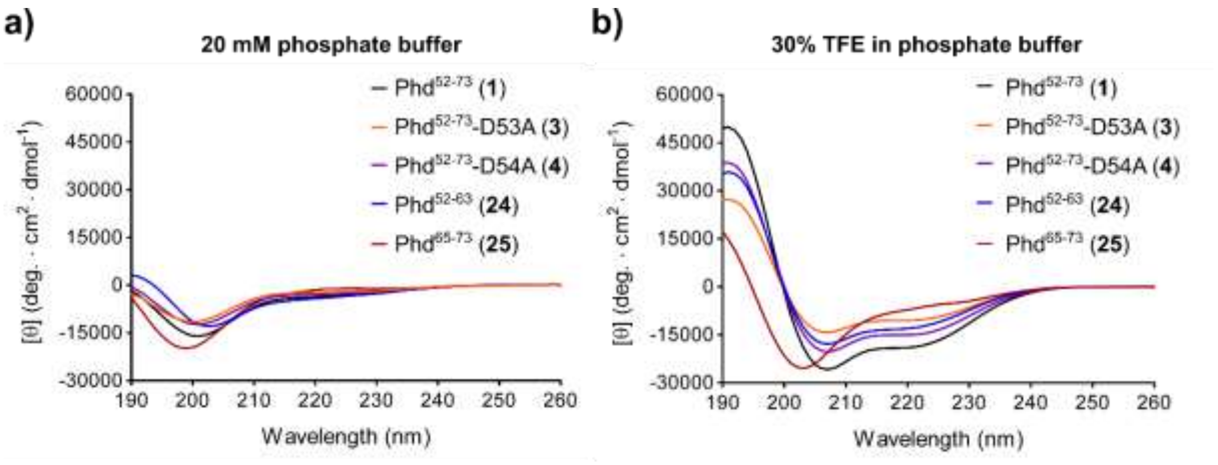

Figure S3. CD spectra of peptides **1**, **3**, **4**, **24** and **25** recorded at 25°C in **(a)** 20 mM potassium phosphate buffer (pH 7.4) and **(b)** 30% (v/v) trifluoroethanol (TFE) in 20 mM potassium phosphate buffer.

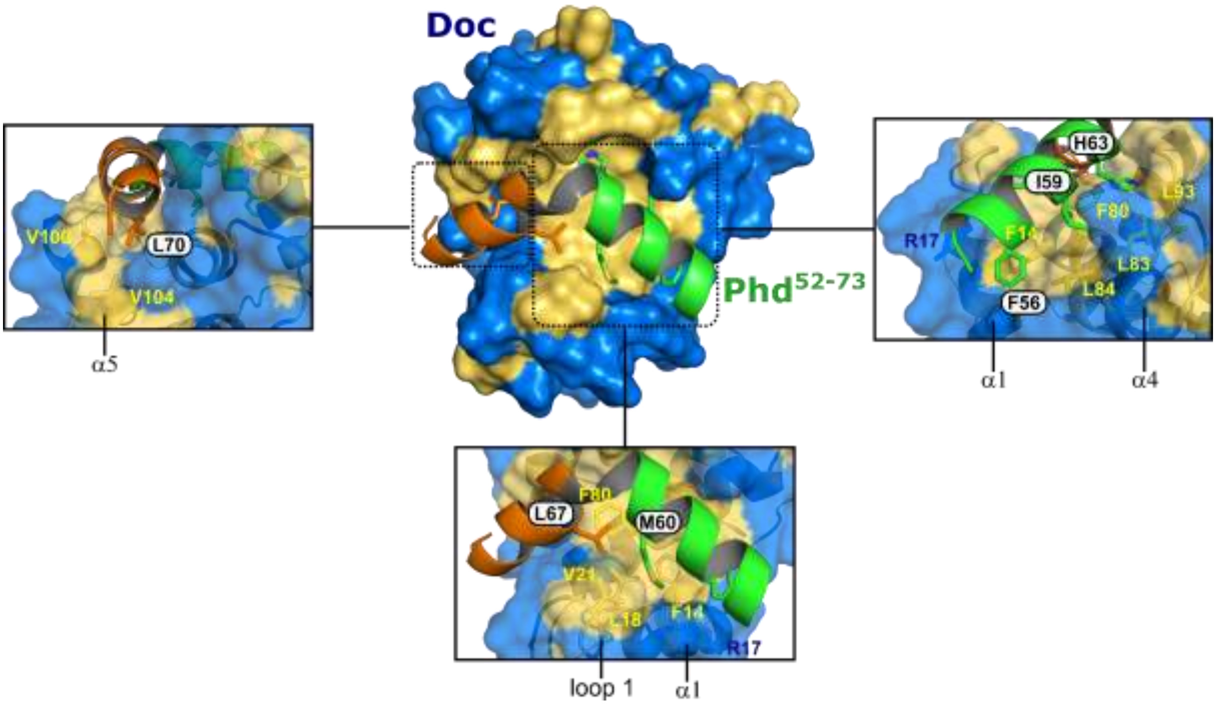

Figure S4. Interaction between Phd_{STm} C-terminal domain and Doc_{STm} in the homology model. Enlarged views highlight the hotspot binding sites and interactions. Hydrophobic residues of Doc_{STm} are coloured in light yellow, with all other residues coloured in blue. The α1 helix of Phd_{STm} is coloured in green, the Gly64 kink in grey and the α2 helix in dark orange.

Table S4. Summary of the differential scanning fluorimetry results of Doc_{STm} at 5 µM in the presence of multiple combinations of peptides **24** (Phd_{STm}^{52-63}) and **25** (Phd_{STm}^{65-73}).

Peptide 24	Peptide 25	Fitting range (°C)	Replicate 1		Replicate 2		Replicate 3		Average T_m (°C)
			T_m (°C)	R^2	T_m (°C)	R^2	T_m (°C)	R^2	
50 µM	-	39.1 – 51.7	46.1	0.9989	46.1	0.9989	45.8	0.9982	46.0 ± 0.1
-	50 µM	37.1 – 50.7	44.1	0.9986	44.2	0.9986	44.4	0.9973	44.2 ± 0.1
100 µM	-	38.1 – 50.2	46.1	0.9993	46.4	0.9991	46.6	0.9981	46.3 ± 0.3
-	100 µM	35.5 - 50.1	43.2	0.9992	43.2	0.9995	43.4	0.9993	43.3 ± 0.1
100 µM	25 µM	40.0 – 52.1	46.1	0.9992	46.1	0.9991	46.2	0.9984	46.2 ± 0.1
100 µM	50 µM	39.0 – 52.0	46.2	0.9993	46.2	0.9992	46.6	0.9969	46.3 ± 0.2
100 µM	100 µM	39.7 – 53.1	46.0	0.9994	46.3	0.9993	46.8	0.9984	46.4 ± 0.4
50 µM	100 µM	39.1 – 52.0	45.5	0.9991	45.7	0.9990	46.0	0.9974	45.7 ± 0.2
25 µM	100 µM	39.6 – 50.5	44.9	0.9987	45.1	0.9990	45.2	0.9986	45.1 ± 0.1

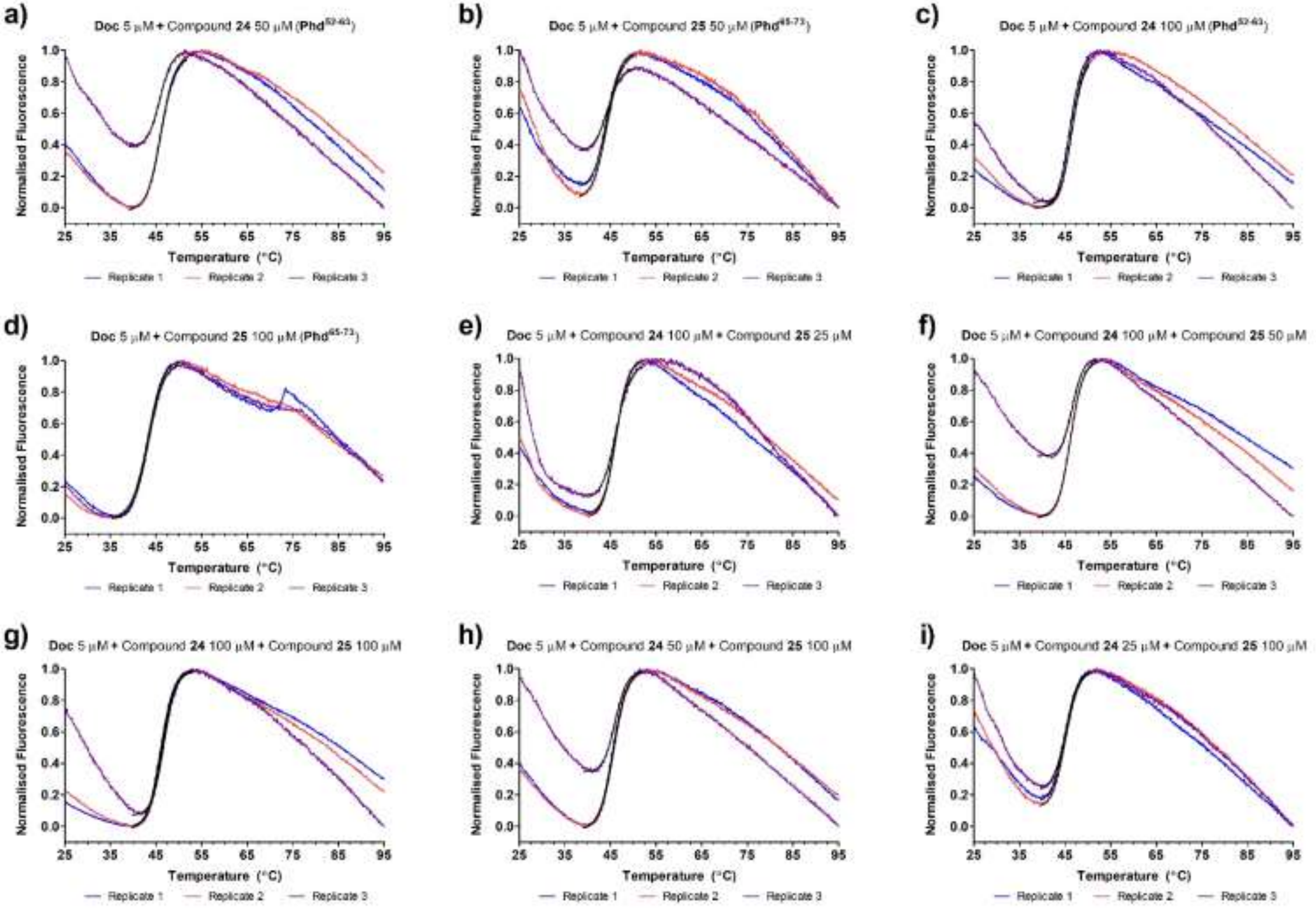

Figure S5. Melting curves of Doc_{STm} in the presence of: **24** at 50 µM (a); **25** at 50 µM (b); **24** at 100 µM (c); **25** at 100 µM (d); **24** at 100 µM and **25** at 25 µM (e); **24** at 100 µM and **25** at 50 µM (f); **24** at 100 µM and **25** at 100 µM (g); **24** at 50 µM and **25** at 100 µM (h); **24** at 25 µM and **25** at 100 µM (i).

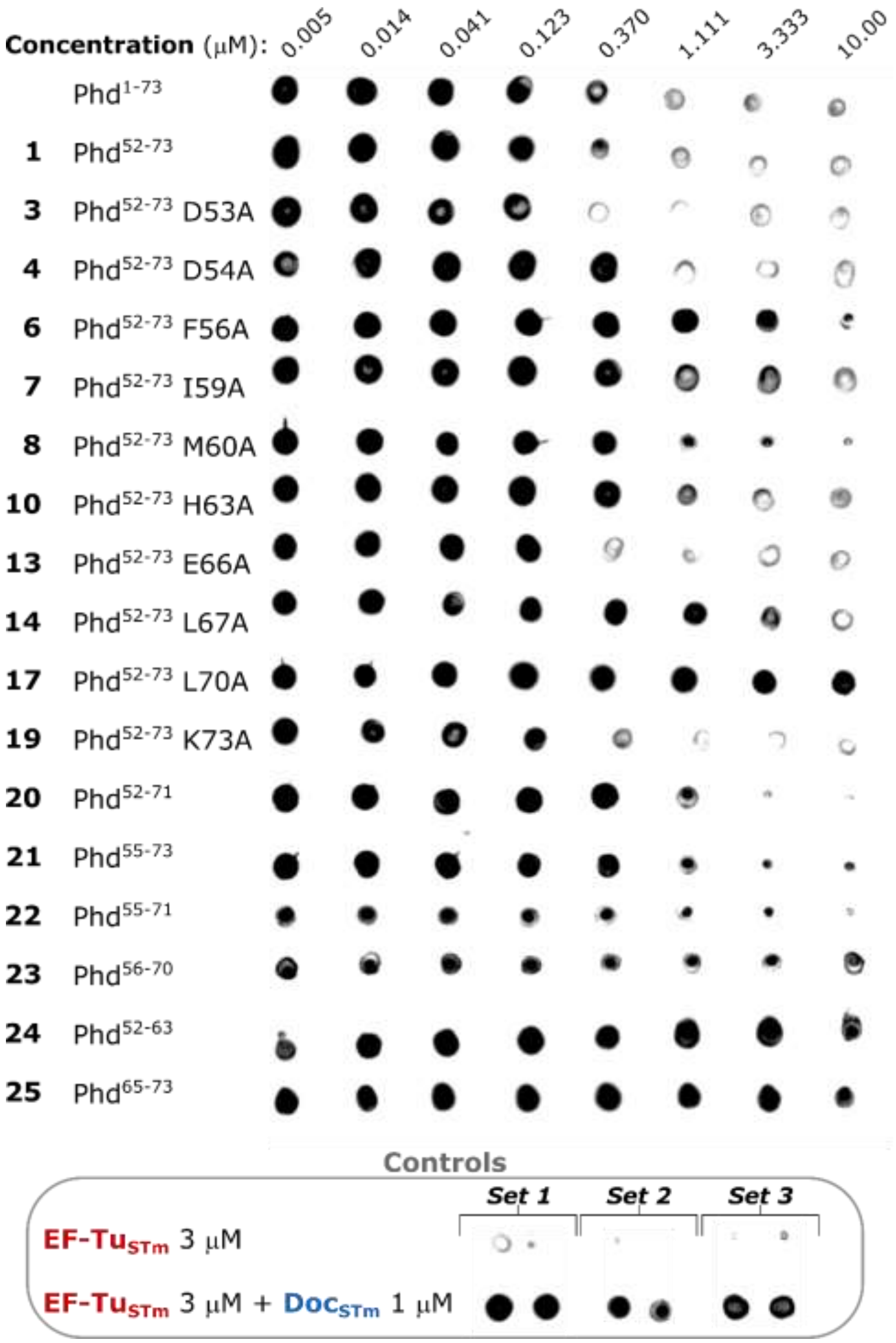

Figure S6. Replicate of the EF-Tu$_{STm}$ phosphorylation assay. Dot blot detection of phosphorylated EF-Tu$_{STm}$ in the presence of Phd$^{1-73}$ or peptides **1**, **3**, **4**, **6**, **7**, **8, 10**, **13**, **14**, **17**, **19**, **20**, **21**, **22**, **23**, **24** and **25**. All peptides were tested at eight concentrations, ranging from 10 μM to 5 nM (3-fold dilutions). Negative (EF-Tu$_{STm}$ 3 μM) and positive (EF-Tu$_{STm}$ 3 μM + Doc$_{STm}$ 1 μM) controls of the assay are shown in the bottom chart. Set 3 of control samples were blotted simultaneously with the reactions using peptides **22** and **23**, set 2 of control samples were blotted simultaneously with the reactions using peptides **6**, **8**, **17**, **20**, **21**, **24** and **25**, while set 1 of control samples were blotted simultaneously with the the remaining peptides shown.

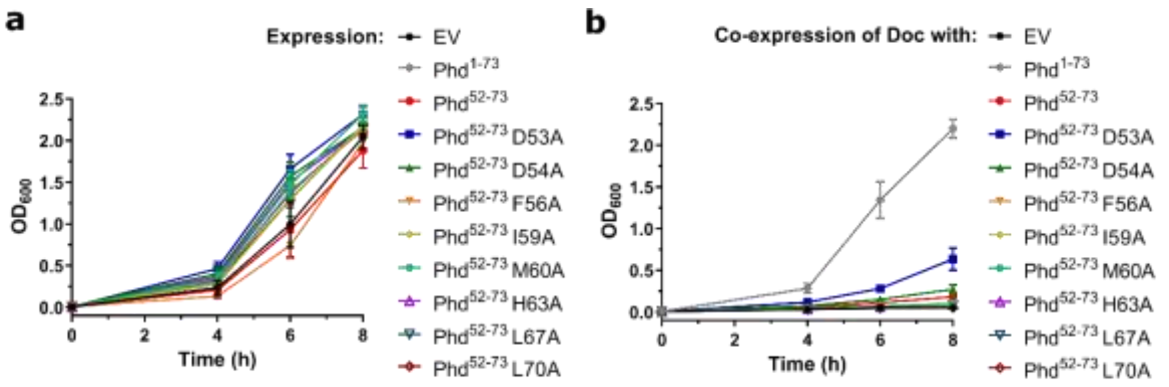

Figure S7. Growth curves (measured as OD_{600}, short incubation of 8 hours) of **(a)** *S.* Typhimurium (14028) *Δphd-doc::Km* control strains transformed with empty pBAD33 vector (no Doc expression) but expressing different Phd_{STm} antitoxin protein and peptide variants from pCA24N plasmid and **(b)** *S.* Typhimurium (14028) *Δphd-doc::Km* strains co-expressing Doc_{STm} (pBAD33) and different Phd_{STm} antitoxin protein and peptide variants (pCA24N). OD_{600} at each time point is the average of four independent experiments. EV: empty vector.

Genotypes of *Salmonella enterica* serovar Typhimurium (14028) strains generated in this study:

Δphd/doc::km pBAD33 pCA24N
Δphd/doc::km pBAD33 pCA24N::phd$^{1-73}$
Δphd/doc::km pBAD33 pCA24N::phd$^{52-73}$
Δphd/doc::km pBAD33 pCA24N::phd$^{52-73,\ D53A}$
Δphd/doc::km pBAD33 pCA24N::phd$^{52-73,\ D54A}$
Δphd/doc::km pBAD33 pCA24N::phd$^{52-73,\ F56A}$
Δphd/doc::km pBAD33 pCA24N::phd$^{52-73,\ I59A}$
Δphd/doc::km pBAD33 pCA24N::phd$^{52-73,\ M60A}$
Δphd/doc::km pBAD33 pCA24N::phd$^{52-73,\ H63A}$
Δphd/doc::km pBAD33 pCA24N::phd$^{52-73,\ L67A}$
Δphd/doc::km pBAD33 pCA24N::phd$^{52-73,\ L70A}$
Δphd/doc::km pBAD33::doc pCA24N
Δphd/doc::km pBAD33::doc pCA24N::phd$^{1-73}$
Δphd/doc::km pBAD33::doc pCA24N::phd$^{1-73,\ D53A}$
Δphd/doc::km pBAD33::doc pCA24N::phd$^{1-73,\ D54A}$
Δphd/doc::km pBAD33::doc pCA24N::phd$^{1-73,\ F56A}$
Δphd/doc::km pBAD33::doc pCA24N::phd$^{1-73,\ I59A}$
Δphd/doc::km pBAD33::doc pCA24N::phd$^{1-73,\ M60A}$
Δphd/doc::km pBAD33::doc pCA24N::phd$^{1-73,\ H63A}$
Δphd/doc::km pBAD33::doc pCA24N::phd$^{1-73,\ L67A}$
Δphd/doc::km pBAD33::doc pCA24N::phd$^{1-73,\ L70A}$
Δphd/doc::km pBAD33::doc pCA24N::phd$^{52-73}$
Δphd/doc::km pBAD33::doc pCA24N::phd$^{52-73,\ D53A}$
Δphd/doc::km pBAD33::doc pCA24N::phd$^{52-73,\ D54A}$
Δphd/doc::km pBAD33::doc pCA24N::phd$^{52-73,\ F56A}$
Δphd/doc::km pBAD33::doc pCA24N::phd$^{52-73,\ I59A}$
Δphd/doc::km pBAD33::doc pCA24N::phd$^{52-73,\ M60A}$
Δphd/doc::km pBAD33::doc pCA24N::phd$^{52-73,\ H63A}$
Δphd/doc::km pBAD33::doc pCA24N::phd$^{52-73,\ L67A}$
Δphd/doc::km pBAD33::doc pCA24N::phd$^{52-73,\ L70A}$

Table S5. Summary of the binding affinities between the Phd_{STm} protein and peptide **1** to Doc_{STm} and equivalent reported *E. coli* P1 phage values.

Organism	Complex	K_d (nM) ^{SPR}	K_d (nM) ^{ITC}	Reference
S. Typhimurium	Doc-Phd^{1-73}	0.061 ± 0.019	-	This study
S. Typhimurium	Doc-Phd^{52-73} (peptide **1**)	0.073 ± 0.018	-	This study
E. coli P1 Phage	Doc-Phd^{1-73}	350	-	[5]
E. coli P1 Phage	Doc^{H66Y}-Phd^{1-73}	1300	-	[5]
E. coli P1 Phage	Doc-Phd^{51-73}	-	240	[5]
E. coli P1 Phage	Doc^{H66Y}-Phd^{52-73}	-	1	[6]

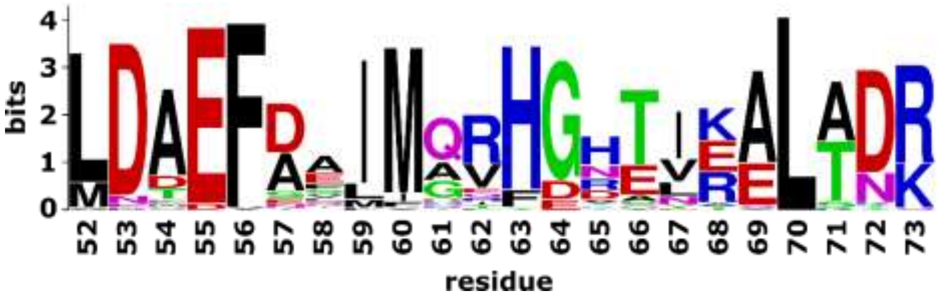

Figure S8. Sequence logo of non-redundant Phd protein sequences from different bacterial species aligned to the Phd$_{STm}$ C-terminal domain (52-73), showing that Phe56 and Leu70 are highly conserved. To generate the logo, the sequence of Phd$_{STm}^{52-73}$ was blasted in NCBI Blast using the RefSeq Select protein database. The resulting multiple sequence alignment was corrected by removing Phd sequences that were shorter than the Phd$_{STm}$ C-terminal 22-amino acid toxin neutralization domain, which yielded a total of 64 unique sequences. The 52-73 amino acid range was selected for the alignment and the logo generated using https://weblogo.berkeley.edu/logo.cgi.

3. Protein constructs, vectors and primers

Table S6. General information of the protein constructs and plasmids prepared.

Protein	Uniprot code	Domain	Mutations	Tags	MW (Da)**	Vector	Cloning sites	Resistance
Doc_{STm}	E8XF70	1 – 122	-	6-Histidine (Ct)	14841.29	pET28	NcoI/XhoI	Kanamycin
Doc_{STm}	E8XF70	1 – 122	-	-	13588	pBAD33[a]	SacI/XbaI	Ampicillin
Phd_{STm}	A0A0F7JE36	1 – 73	-	6-Histidine* and FLAG (Nt)	9996.10 (11878.15)	pET28-FLAG	NheI/BamHI	Ampicillin
Phd_{STm}	A0A0F7JE36	1 – 73	Various mutants	-	varied	pCA24N	MfeI/NotI	Chloramphenicol
Phd_{STm}	A0A0F7JE36	52 – 73	Various mutants	-	varied	pCA24N	MfeI/NotI	Chloramphenicol
EF-Tu_{STm}	P0A1H5	1 – 394	-	-	43283.55	pBAD33[a]	SacI/HindIII	Ampicillin
EF-Tu_{STm}	P0A1H5	1 – 394	T383A	-	43253.53	pBAD33[a]	SacI/HindIII	Ampicillin
EF-Tu_{STm}	P0A1H5	1 – 394	T383V	-	43281.58	pBAD33[a]	SacI/HindIII	Ampicillin
EF-Tu_{STm}	P0A1H5	1 – 394	-	6-Histidine (Ct)	44348.67	pET24a	NdeI/XhoI	Kanamycin
EF-Tu_{STm}	P0A1H5	1 – 394	T383A	6-Histidine (Ct)	44318.65	pET24a	NdeI/XhoI	Kanamycin
EF-Tu_{STm}	P0A1H5	1 – 394	T383V	6-Histidine (Ct)	44346.7	pET24a	NdeI/XhoI	Kanamycin

[a]A modified pBAD33 vector with flipped resistance cassette (ampicillin instead of chloramphenicol)[1] was used. Nt: N-terminus; Ct: C-terminus; MW : Molecular weight.

* Can be removed by thrombin digestion.** Values in parentheses correspond to the molecular weight before tag cleavage.

Table S7. Primer sequences used for the cloning and mutagenesis of Doc, Phd and EF-Tu constructs.[a]

Cloning primer	Target vector	Primer sequence
Doc_NcoI_fw	pET28	GGTAATCCATGGGCATGACCCTACAACTTATCTCAGC
Doc_XhoI_rv	pET28	GGTAATCTCGAGGGCACGTAAGCGAACG
Phd_NheI_fw	pET-FLAG	GGTGGTGCTAGCATGTTTATGCGTACGG
Phd_BamHI_rv	pET-FLAG	GGTAATGGATCCTCATTTATCCGCCAGCTCC
EF-Tu_SacI_fw	pBAD33	GGTGGTGAGCTCATGTCTAAAGAAAAGTTTGAACG
EF-Tu_HindIII_rv	pBAD33	CCACCAAAGCTTTTAGCCGAGAACTTTAGC
EF-Tu_NdeI_fw	pET24a	GGTGGTCATATGTCTAAAGAAAAGTTTGAACG
EF-Tu_Xho1_rv	pET24a	CCATTACTCGAGGCCGAGAACTTTAGCAACA
pBAD_RBS_fw	pBAD33	pCTAGCAGGAGGAAGAGCT[b]
pBAD_RBS_rv	pBAD33	pCTTCCTCCTG[b]
**Mutagenesis primer**	**Target vector**	**Primer sequence**
EF-Tu_T383A_HindIII_rv	pBAD33	CCTATTAAGCTTTTAGCCGAGAACTTTAGCAACAACGCCCGCGCCAACGGCACGGCC
EF-Tu_T383A_XhoI_rv	pET24a	CCTATTCTCGAGGCCGAGAACTTTAGCAACAACGCCCGCGCCAACGGCACGGCC
EF-Tu_T383V_fw	pBAD33/pET24a	GGCGGCCGTGTAGTTGGCGCG
EF-Tu_T383V_rv	pBAD33/pET24a	TTCACGGATTGCGAAACGCAGACCGTCG

[a]fw: forward primer, rv: reverse primer, [b]5' phosphorylated primer

Table S8. Full amino acid sequences of the proteins purified in this work. The desired protein sequence is underlined, mutated residues are highlighted in red, tags are highlighted in purple and cleavage sites are indicated with a slash in the middle of the sequence.

**Protein**	**Sequence**
Doc$^{1\text{-}122}$–His$_6$	MGMTLQLISAEEIIQFHDRLLRVTPGVTGMPDPGRAEALMYRVLKQIEYEGVTDVWLLAAMHLLAISRGHIFNDGNKRTALFIT<br>LLFLKRNGISLAANPDFVDMTVDAAAGRLTLEQIAVRLRALEHHHHHH
His$_6$ / FLAG–Phd$^{1\text{-}73}$	MGSSHHHHHHSSGLVPR/GSHMDYKDDDDKASMFMRTVNYSEARQNLAEVLESAVTGGPVTITRRGHKSAVIISAEEFERYQTA<br>RMDDEFAAIMAVHGNELRELADK
EF-Tu$^{1\text{-}394}$–His$_6$ (pET24a)	MSKEKFERTKPHVNVGTIGHVDHGKTTLTAAITTVLAKTYGGAARAFDQIDNAPEEKARGITINTSHVEYDTPTRHYAHVDCPG<br>HADYVKNMITGAAQMDGAILVVAATDGPMPQTREHILLGRQVGVPYIIVFLNKCDMVDDEELLELVEMEVRELLSQYDFPGDDT<br>PIVRGSALKALEGDAEWEAKIIELAGFLDSYIPEPERAIDKPFLLPIEDVFSISGRGTVVTGRVERGIIKVGEEVEIVGIKETQ<br>KSTCTGVEMFRKLLDEGRAGENVGVLLRGIKREEIERGQVLAKPGTIKPHTKFESEVYILSKDEGGRHTPFFKGYRPQFYFRTT<br>DVTGTIELPEGVEMVMPGDNIKMVVTLIHPIAMDDGLRFAIREGGRTVGAGVVAKVLGLEHHHHHH
EF-Tu$^{1\text{-}394}$ T383A–His$_6$ (pET24a)	MSKEKFERTKPHVNVGTIGHVDHGKTTLTAAITTVLAKTYGGAARAFDQIDNAPEEKARGITINTSHVEYDTPTRHYAHVDCPG<br>HADYVKNMITGAAQMDGAILVVAATDGPMPQTREHILLGRQVGVPYIIVFLNKCDMVDDEELLELVEMEVRELLSQYDFPGDDT<br>PIVRGSALKALEGDAEWEAKIIELAGFLDSYIPEPERAIDKPFLLPIEDVFSISGRGTVVTGRVERGIIKVGEEVEIVGIKETQ<br>KSTCTGVEMFRKLLDEGRAGENVGVLLRGIKREEIERGQVLAKPGTIKPHTKFESEVYILSKDEGGRHTPFFKGYRPQFYFRTT<br>DVTGTIELPEGVEMVMPGDNIKMVVTLIHPIAMDDGLRFAIREGGR**A**VGAGVVAKVLGLEHHHHHH
EF-Tu$^{1\text{-}394}$ T383V–His$_6$ (pET24a)	MSKEKFERTKPHVNVGTIGHVDHGKTTLTAAITTVLAKTYGGAARAFDQIDNAPEEKARGITINTSHVEYDTPTRHYAHVDCPG<br>HADYVKNMITGAAQMDGAILVVAATDGPMPQTREHILLGRQVGVPYIIVFLNKCDMVDDEELLELVEMEVRELLSQYDFPGDDT<br>PIVRGSALKALEGDAEWEAKIIELAGFLDSYIPEPERAIDKPFLLPIEDVFSISGRGTVVTGRVERGIIKVGEEVEIVGIKETQ<br>KSTCTGVEMFRKLLDEGRAGENVGVLLRGIKREEIERGQVLAKPGTIKPHTKFESEVYILSKDEGGRHTPFFKGYRPQFYFRTT<br>DVTGTIELPEGVEMVMPGDNIKMVVTLIHPIAMDDGLRFAIREGGR**V**VGAGVVAKVLGLEHHHHHH

4. Differential scanning fluorimetry data

Table S9. Summary of the DSF results obtained with Doc_{STm} at 5 µM. In all cases the sigmoidal fit was applied in the temperature range of 30.0 to 52.0 °C.

Replicate	T_m (°C)	R^2
1	42.46	0.9997
2	41.91	0.9997
3	41.48	0.9993
4	44.47	0.9996
5	43.57	0.9996
6	42.59	0.9995
7	42.89	0.9996
8	43.53	0.9993
9	43.06	0.9997
**Average**	**42.9 ± 0.9**	**-**

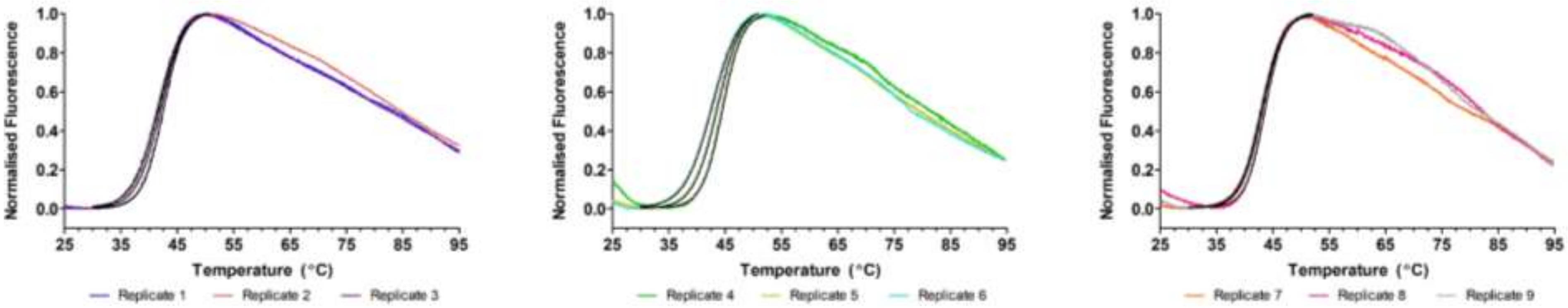

Figure S9. Melting curves of Doc_{STm} at 5 µM. Replicates are split into three graphs for better visualisation of the curves.

Table S10. Summary of the DSF results of Doc_{STm} at 5 µM in the presence of 50 µM of Phd_{STm}^{1-73} and peptides **1–23**.

Binder	Fitting range (°C)	Replicate 1		Replicate 2		Replicate 3		Average T_m (°C)
		T_m (°C)	R^2	T_m (°C)	R^2	T_m (°C)	R^2	
Phd^{1-73}	65.7 - 78.4	73.6	0.9993	73.9	0.9993	74.9	0.9994	74.2 ± 0.7
**1** (Phd_{STm}^{52-73})	63.2 - 76.0	71.8	0.9996	71.5	0.9996	71.6	0.9997	71.7 ± 0.1
**2** (Phd_{STm}^{52-73}-M52A)	63.2 – 74.7	71.3	0.9995	70.8	0.9995	70.6	0.9995	70.9 ± 0.4
**3** (Phd_{STm}^{52-73}-D53A)	59.1 – 72.9	68.3	0.9997	68.4	0.9997	68.8	0.9997	68.5 ± 0.3
**4** (Phd_{STm}^{52-73}-D54A)	61.5 – 72.5	69.0	0.9994	69.3	0.9993	69.1	0.9994	69.1 ± 0.2
**5** (Phd_{STm}^{52-73}-E55A)	59.6 – 72.9	66.3	0.9997	66.3	0.9997	66.2	0.9996	66.3 ± 0.1
**6** (Phd_{STm}^{52-73}-F56A)	46.7 – 70.4	64.5	0.9957	64.1	0.9963	63.5	0.9968	64.0 ± 0.5
**7** (Phd_{STm}^{52-73}-I59A)	48.0 – 67.7	59.7	0.9986	57.7	0.9987	57.6	0.9993	58.4 ± 1.2
**8** (Phd_{STm}^{52-73}-M60A)	54.6 – 68.5	64.1	0.9994	63.3	0.9994	63.7	0.9995	63.7 ± 0.4
**9** (Phd_{STm}^{52-73}-V62A)	58.0 – 72.9	68.3	0.9997	67.4	0.9997	66.9	0.9996	67.5 ± 0.7
**10** (Phd_{STm}^{52-73}-H63A)	51.1 – 68.9	63.3	0.9991	63.7	0.9996	64.2	0.9995	63.7 ± 0.4
**11** (Phd_{STm}^{52-73}-G64A)	59.0 – 74.7	68.8	0.9995	69.2	0.9997	68.9	0.9997	69.0 ± 0.2
**12** (Phd_{STm}^{52-73}-N65A)	60.7 – 75.2	71.8	0.9996	71.0	0.9997	70.4	0.9993	71.1 ± 0.7
**13** (Phd_{STm}^{52-73}-E66A)	62.0 – 74.5	70.8	0.9996	70.4	0.9995	70.6	0.9995	70.6 ± 0.2
**14** (Phd_{STm}^{52-73}-L67A)	47.2 – 64.5	57.4	0.9998	57.2	0.9998	57.0	0.9998	57.2 ± 0.2
**15** (Phd_{STm}^{52-73}-R68A)	62.2 – 74.5	70.6	0.9997	70.7	0.9996	70.4	0.9997	70.6 ± 0.1
**16** (Phd_{STm}^{52-73}-E69A)	63.6 – 78.2	73.5	0.9996	73.5	0.9996	73.9	0.9996	73.6 ± 0.2
**17** (Phd_{STm}^{52-73}-L70A)	47.5 – 65.5	58.5	0.9997	57.9	0.9997	57.8	0.9997	58.0 ± 0.4
**18** (Phd_{STm}^{52-73}-D72A)	60.0 – 74.9	71.1	0.9996	71.0	0.9996	70.6	0.9992	70.9 ± 0.3
**19** (Phd_{STm}^{52-73}-K73A)	57.0 – 72.7	68.8	0.9997	67.6	0.9997	67.7	0.9997	68.0 ± 0.6
**20** (Phd_{STm}^{52-71})	58.0 – 72.5	68.5	0.9995	68.4	0.9994	68.5	0.9994	68.5 ± 0.1
**21** (Phd_{STm}^{55-73})	53.3 – 66.5	62.3	0.9995	62.0	0.9995	62.4	0.9995	62.2 ± 0.2
**22** (Phd_{STm}^{55-71})	43.5 – 55.7	58.89	0.9995	59.16	0.9995	59.42	0.9994	59.2 ± 0.3
**23** (Phd_{STm}^{56-70})	52.0 – 63.7	50.54	0.9995	50.60	0.9996	51.08	0.9996	50.7 ± 0.3

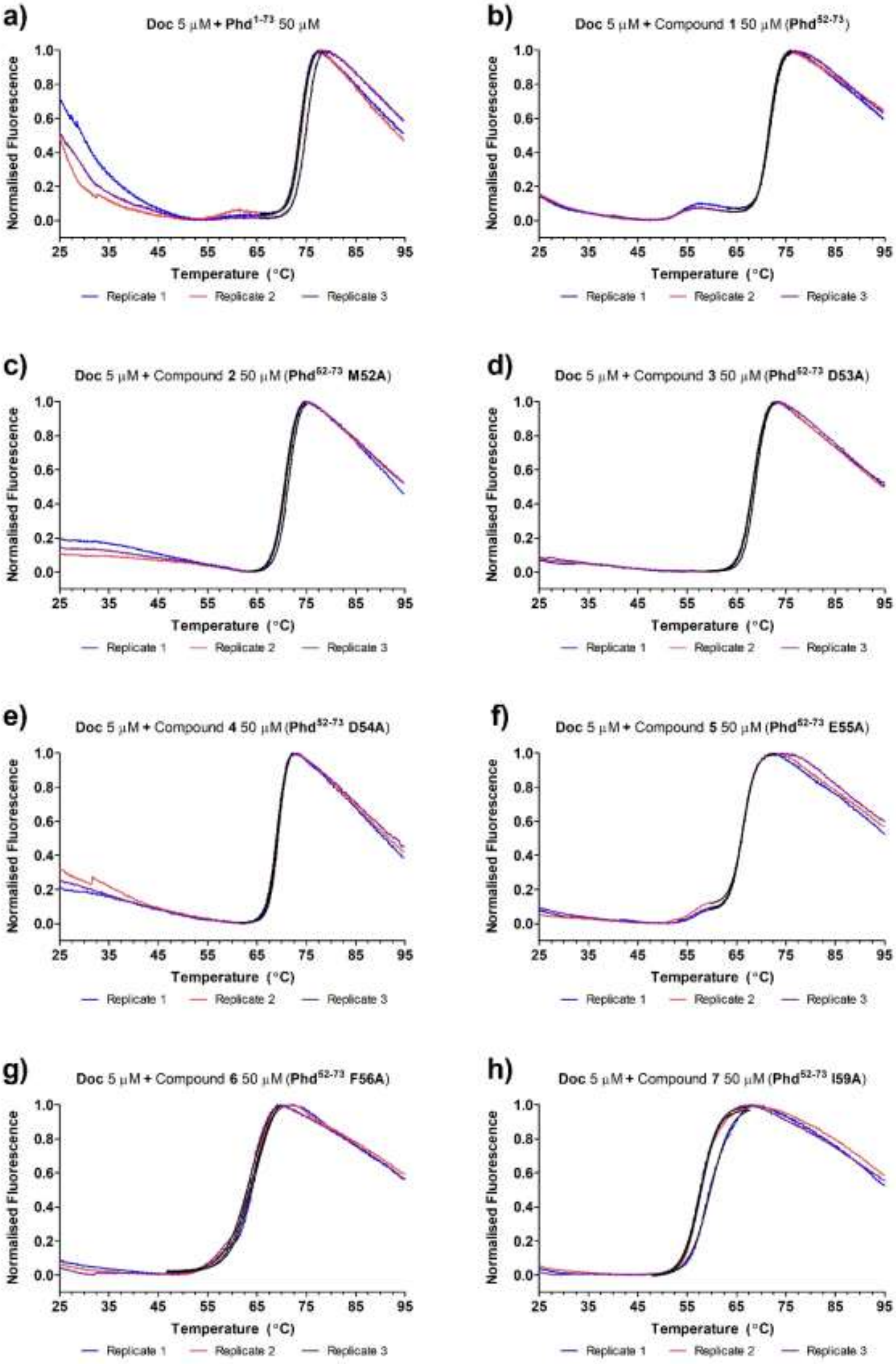

Figure S10. Melting curves of Doc_{STm} at 5 µM in the presence of 50 µM of $Phd_{STm}^{1\text{-}73}$ (a), **1** (b), **2** (c), **3** (d), **4** (e), **5** (f), **6** (g), **7** (h).

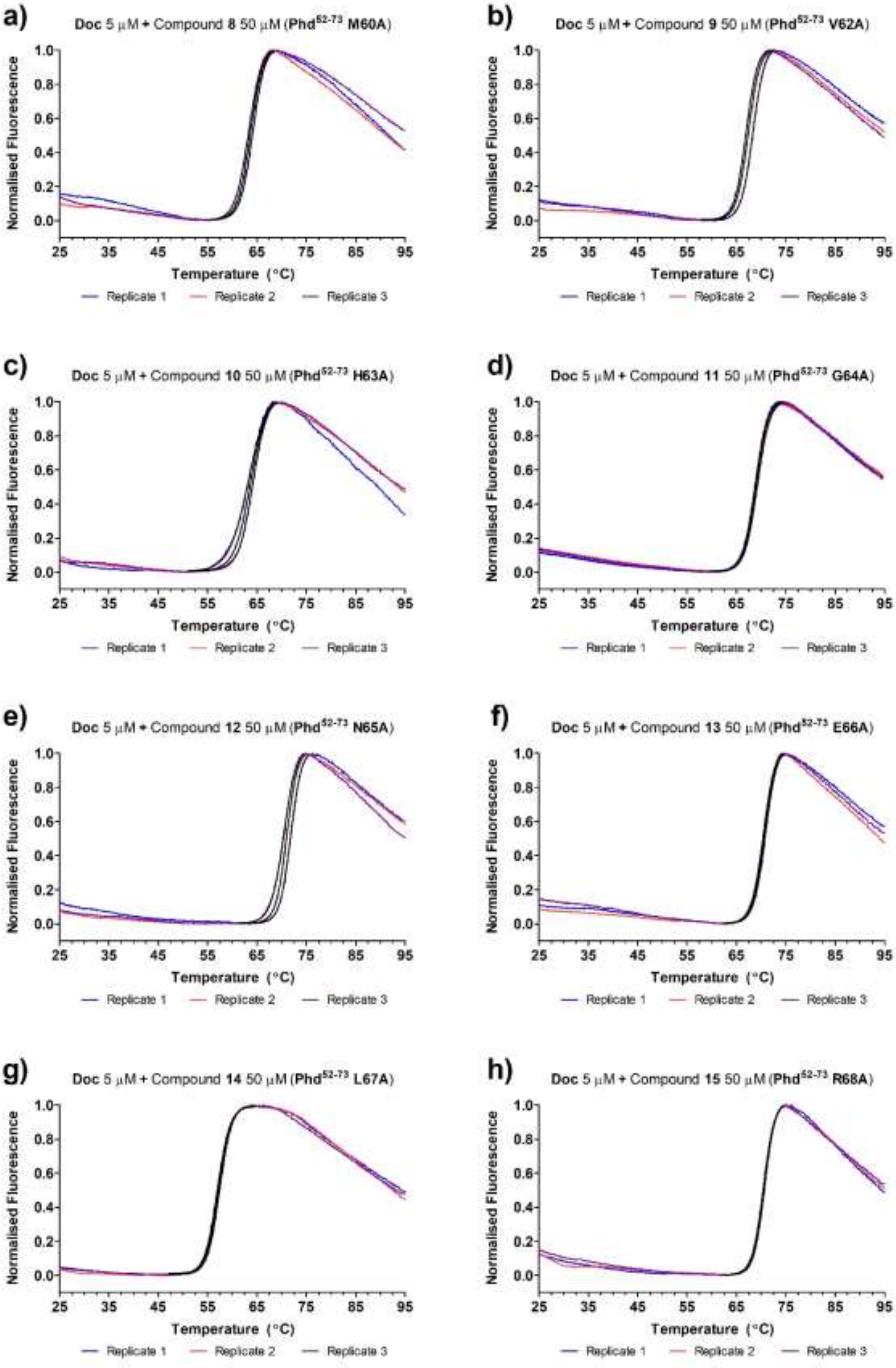

Figure S11. Melting curves of Doc_{STm} at 5 μM in the presence of 50 μM of **8** (a), **9** (b), **10** (c), **11** (d), **12** (e), **13** (f), **14** (g) and **15** (h).

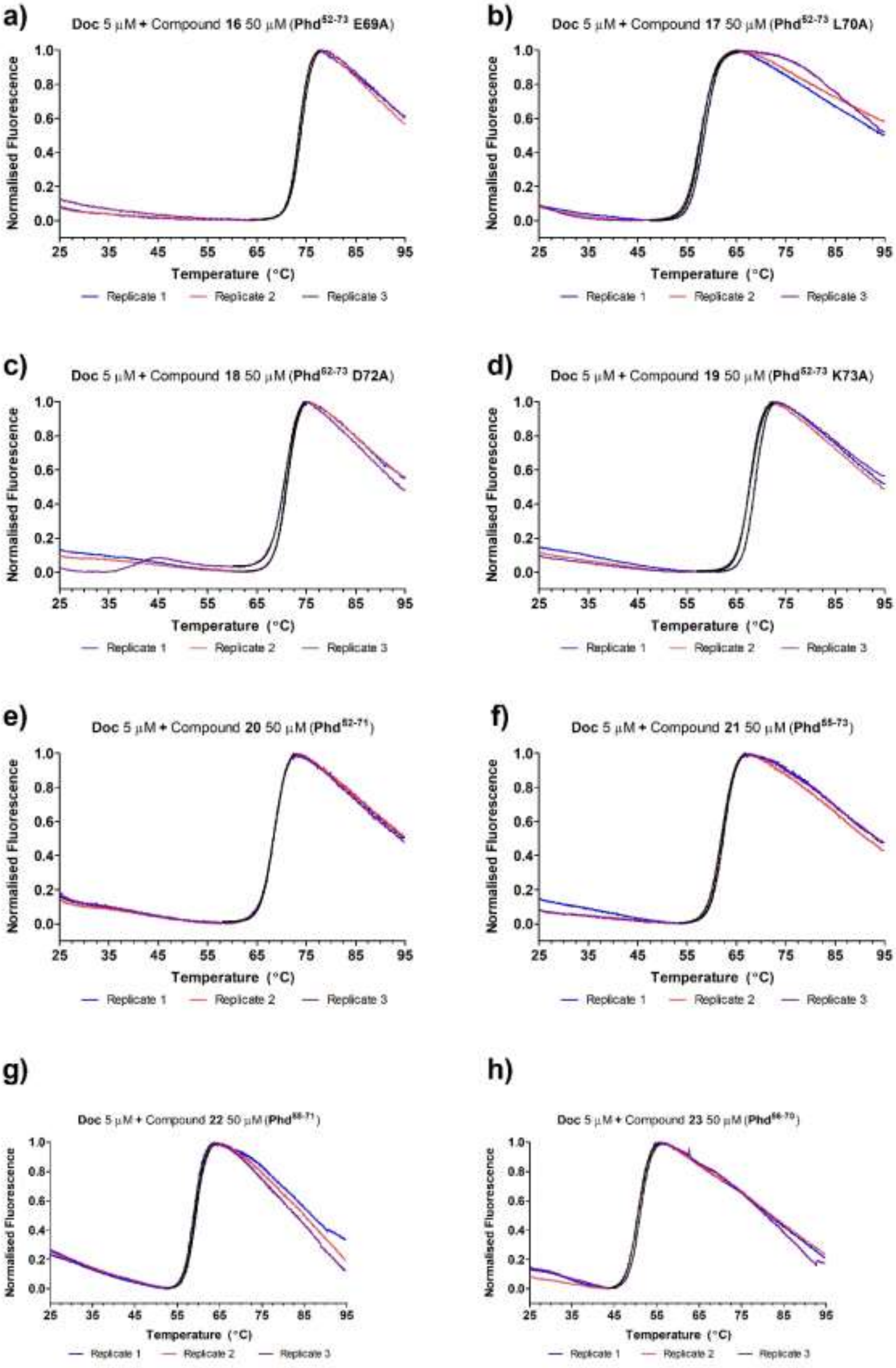

Figure S12. Melting curves of Doc_{STm} at 5 µM in the presence of 50 µM of **16** (a), **17** (b), **18** (c), **19** (d), **20** (e), **21** (f), **22** (g) and **23** (h).

5. Surface plasmon resonance data

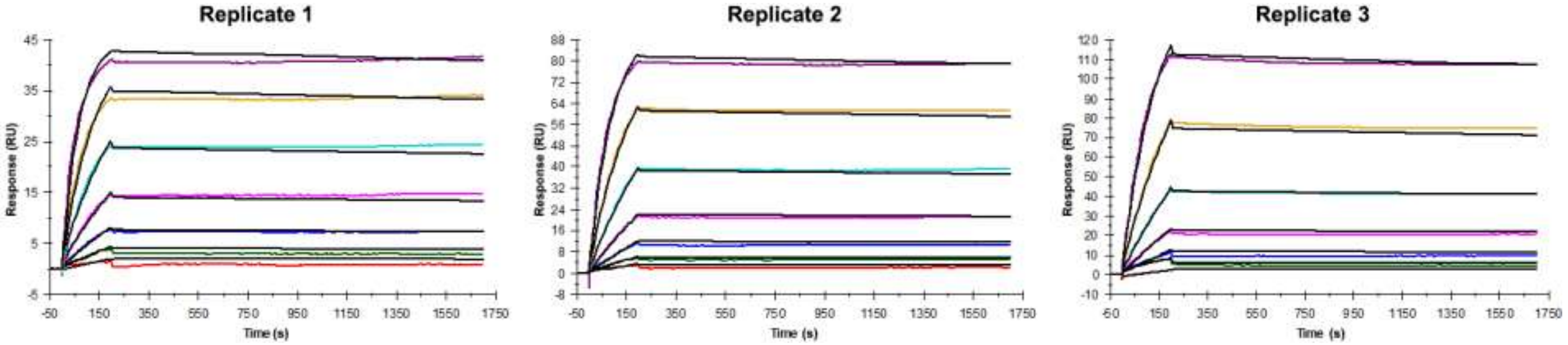

Figure S13. Sensorgrams obtained for each replicate of the $\mathrm{Phd}_{\mathrm{STm}}^{1\text{-}73}$ protein (analyte) binding to $\mathrm{Doc}_{\mathrm{STm}}$ (ligand). Due to struggles to fit the slow kinetic rate constants via single-cycle experiments, eight concentrations of the analyte were flown over the surface in multiple individual cycles to obtain more datapoints on the dissociation phase. Analyte concentrations ranged from 25 nM to 0.390625 nM (2-fold dilutions) and the association and dissociation times were 200 s and 1500 s, respectively. The surface was regenerated between each cycle and the ligand density on the surface varied between replicates (lowest and highest densities correspond to replicates 1 and 3, respectively) to verify the reproducibility of the results. Due to the slow association rate and mass-transport limitation at higher analyte concentrations, the data was fitted with a constant R_{MAX}, which was calculated based on the R_{MAX} of a positive control (peptide **1**).

Table S11. Summary of the fitted parameters obtained from each SPR measurement of the $\mathrm{Phd}_{\mathrm{STm}}^{1\text{-}73}$ protein.

Replicate	k_{on} (M^{-1} s^{-1})	k_{off} (s^{-1})	K_D (pM)	R_{MAX} (RU)	t_C (RU M^{-1} s^{-1})	Chi^2 (RU^2)	U-value
**1**	$5.958 \pm 0.001 \times 10^5$	$3.022 \pm 0.008 \times 10^{-5}$	50.7	45.0	$5.224 \pm 0.020 \times 10^{10}$	0.923	15
**2**	$4.357 \pm 0.001 \times 10^5$	$2.200 \pm 0.008 \times 10^{-5}$	50.5	91.8	$2.354 \pm 2.900 \times 10^{12}$	1.300	12
**3**	$3.873 \pm 0.001 \times 10^5$	$3.421 \pm 0.002 \times 10^{-5}$	88.3	139.9	$4.360 \pm 0.003 \times 10^7$	2.530	9

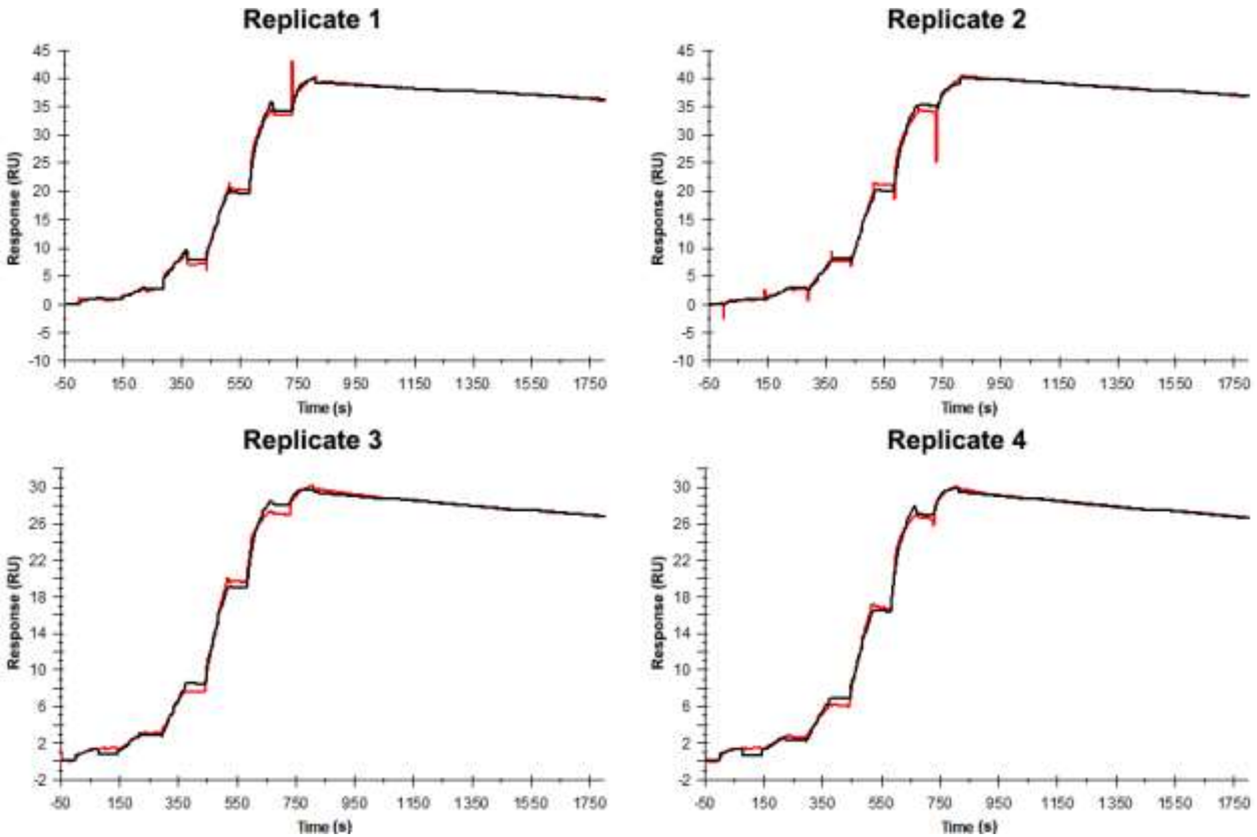

Figure S14. Sensorgrams obtained for each replicate of peptide **1** / $Phd_{STm}^{52\text{-}73}$ (analyte) binding to Doc_{STm} (ligand). Six concentrations of the analyte were flown over the surface in a single cycle, ranging from 50 nM to 0.206 nM (3-fold dilutions). The association and dissociation times were 80 s and 1000 s, respectively.

Table S12. Summary of the fitted parameters obtained from each SPR measurement of peptide **1**.

Replicate	k_{on} (M^{-1} s^{-1})	k_{off} (s^{-1})	K_D (pM)	R_{MAX} (RU)	t_C (RU M^{-1} s^{-1})	Chi^2 (RU^2)	U-value
**1**	$1.056 \pm 0.001 \times 10^6$	$7.750 \pm 0.040 \times 10^{-5}$	73.4	39.20 ± 0.01	$1.694 \pm 15.000 \times 10^{13}$	0.195	3
**2**	$1.065 \pm 0.001 \times 10^6$	$8.926 \pm 0.054 \times 10^{-5}$	83.8	40.20 ± 0.01	$2.540 \pm 1.100 \times 10^{11}$	0.371	3
**3**	$1.610 \pm 0.002 \times 10^6$	$9.520 \pm 0.047 \times 10^{-5}$	59.1	29.40 ± 0.01	$1.670 \pm 11.200 \times 10^{13}$	0.157	3
**4**	$1.270 \pm 0.002 \times 10^6$	$10.40 \pm 0.040 \times 10^{-5}$	81.9	29.50 ± 0.01	$2.430 \pm 0.986 \times 10^9$	0.113	2

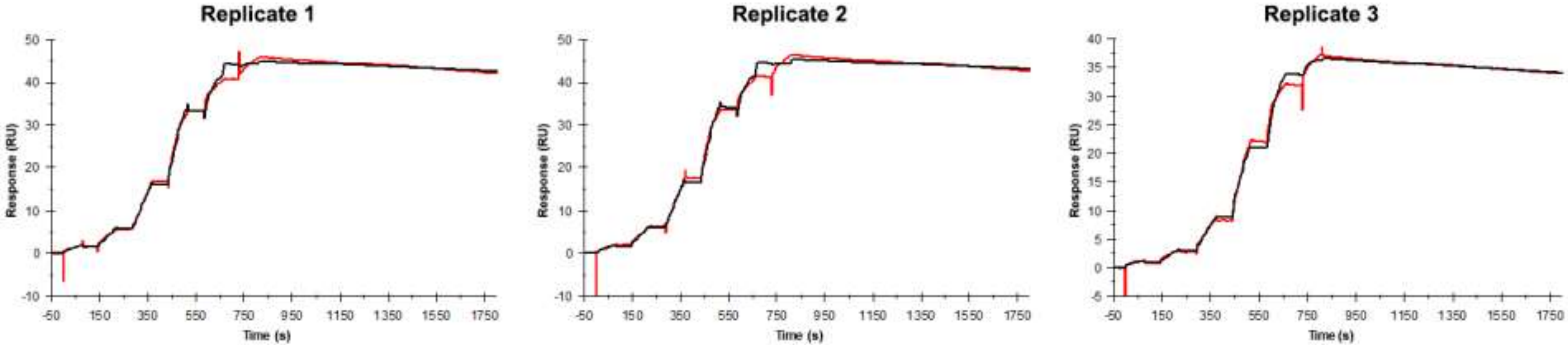

Figure S15. Sensorgrams obtained for each replicate of peptide **2** / $Phd_{STm}^{52\text{-}73}$ M52A (analyte) binding to Doc_{STm} (ligand). Six concentrations of the analyte were flown over the surface in a single cycle, ranging from 100 nM to 0.412 nM (3-fold dilutions) for replicates 1 and 2, and 50 nM to 0.206 nM (3-fold dilutions) for replicate 3. The association and dissociation times were 80 s and 1000 s, respectively.

Table S13. Summary of the fitted parameters obtained from each SPR measurement of peptide **2**.

Replicate	k_{on} (M^{-1} s^{-1})	k_{off} (s^{-1})	K_D (pM)	R_{MAX} (RU)	t_C (RU M^{-1} s^{-1})	Chi^2 (RU^2)	U-value
**1**	$1.039 \pm 0.002 \times 10^6$	$5.437 \pm 0.066 \times 10^{-5}$	52.3	44.9 ± 0.02	$1.311 \pm 0.500 \times 10^{12}$	0.777	7
**2**	$1.072 \pm 0.002 \times 10^6$	$5.126 \pm 0.065 \times 10^{-5}$	47.8	45.3 ± 0.02	$2.159 \pm 0.850 \times 10^{12}$	0.792	7
**3**	$1.297 \pm 0.002 \times 10^6$	$7.143 \pm 0.057 \times 10^{-5}$	55.1	36.7 ± 0.01	$1.249 \pm 6.100 \times 10^{13}$	0.367	5

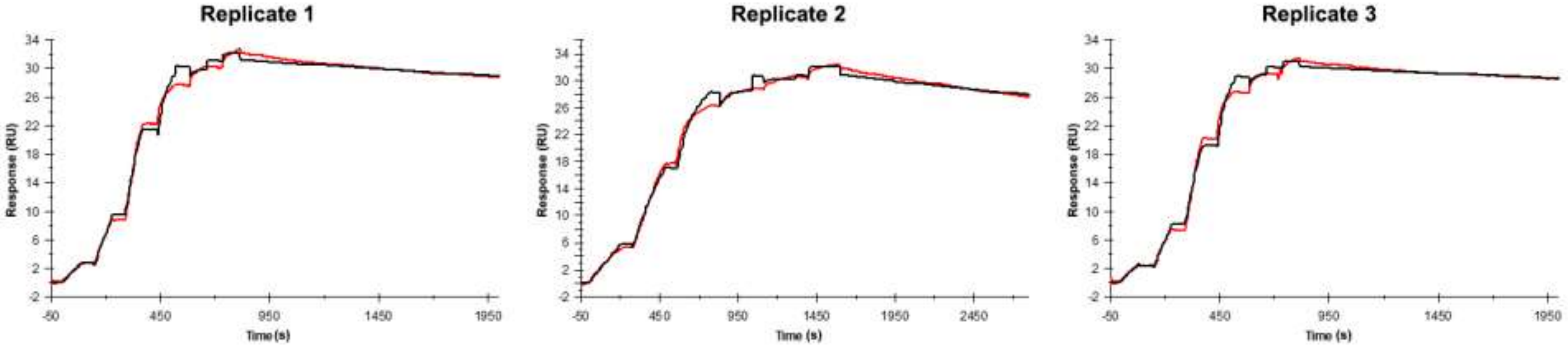

Figure S16. Sensorgrams obtained for each replicate of peptide **3** / $Phd_{STm}^{52\text{-}73}$ D53A (analyte) binding to Doc_{STm} (ligand). Six concentrations of the analyte were flown over the surface in a single cycle, ranging from 50 nM to 0.206 nM (3-fold dilutions). The association times were 80s for replicates 1 and 2 and 200 s for replicate 3. In all replicates the dissociation time was 1200 s.

Table S14. Summary of the fitted parameters obtained from each SPR measurement of peptide **3**.

Replicate	k_{on} (M^{-1} s^{-1})	k_{off} (s^{-1})	K_D (pM)	R_{MAX} (RU)	t_C (RU M^{-1} s^{-1})	Chi^2 (RU^2)	U-value
**1**	$5.480 \pm 0.010 \times 10^{6}$	$6.650 \pm 0.052 \times 10^{-5}$	12.1	31.2 ± 0.01	$4.970 \pm 3.920 \times 10^{12}$	0.434	5
**2**	$4.980 \pm 0.009 \times 10^{6}$	$8.250 \pm 0.052 \times 10^{-5}$	16.6	30.8 ± 0.01	$1.280 \pm 3.230 \times 10^{14}$	0.449	5
**3**	$4.760 \pm 0.008 \times 10^{6}$	$4.720 \pm 0.049 \times 10^{-5}$	9.92	30.2 ± 0.01	$3.270 \pm 9.670 \times 10^{13}$	0.358	7

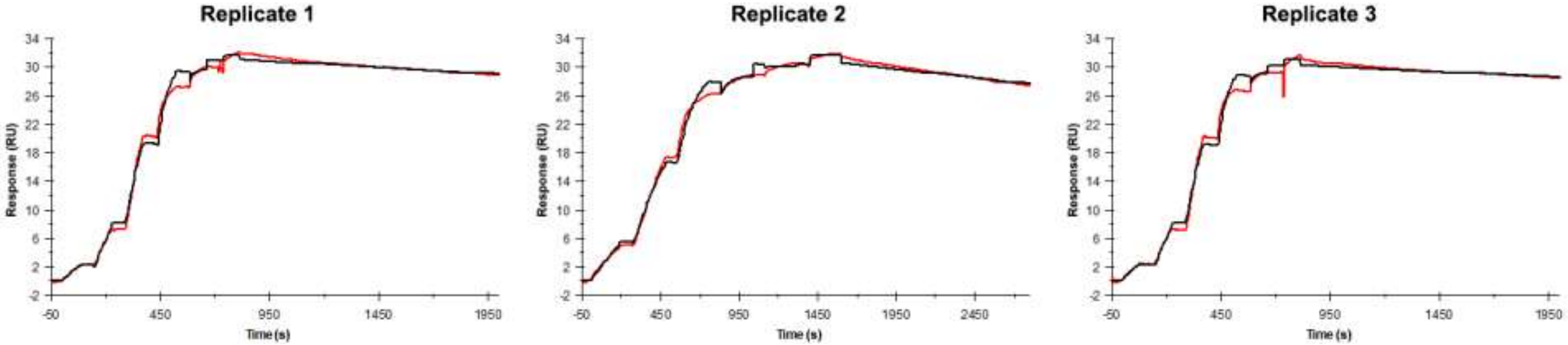

Figure S17. Sensorgrams obtained for each replicate of peptide **4** / Phd_{STm}^{52-73} D54A (analyte) binding to Doc_{STm} (ligand). Six concentrations of the analyte were flown over the surface in a single cycle, ranging from 50 nM to 0.206 nM (3-fold dilutions). The association times were 80s for replicates 1 and 2 and 200 s for replicate 3. In all replicates the dissociation time was 1200 s.

Table S15. Summary of the fitted parameters obtained from each SPR measurement of peptide **4**.

Replicate	k_{on} (M^{-1} s^{-1})	k_{off} (s^{-1})	K_D (pM)	R_{MAX} (RU)	t_C (RU M^{-1} s^{-1})	Chi^2 (RU^2)	U-value
**1**	$4.580 \pm 0.008 \times 10^6$	$5.840 \pm 0.050 \times 10^{-5}$	12.8	31.1 ± 0.01	$3.560 \pm 3.570 \times 10^{14}$	0.388	5
**2**	$4.890 \pm 0.007 \times 10^6$	$8.120 \pm 0.046 \times 10^{-5}$	16.6	30.6 ± 0.01	$2.390 \pm 2.430 \times 10^{13}$	0.347	5
**3**	$4.730 \pm 0.008 \times 10^6$	$4.760 \pm 0.049 \times 10^{-5}$	10.1	30.2 ± 0.01	$4.950 \pm 9.790 \times 10^{12}$	0.365	7

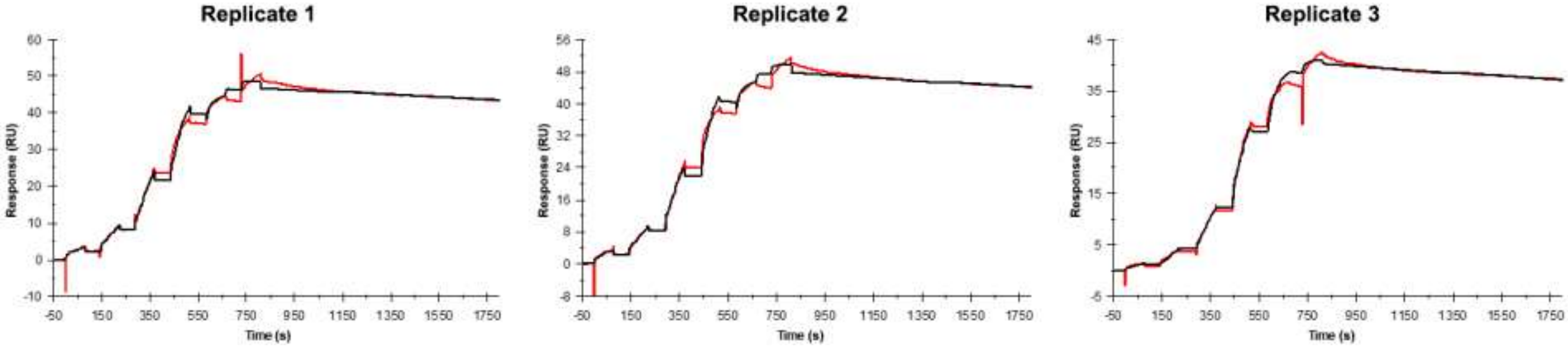

Figure S18. Sensorgrams obtained for each replicate of peptide **5** / $Phd_{STm}^{52\text{-}73}$ E55A (analyte) binding to Doc_{STm} (ligand). Six concentrations of the analyte were flown over the surface in a single cycle, ranging from 100 nM to 0.412 nM (3-fold dilutions) for replicates 1 and 2, and 50 nM to 0.206 nM (3-fold dilutions) for replicate 3. The association and dissociation times were 80 s and 1000 s, respectively.

Table S16. Summary of the fitted parameters obtained from each SPR measurement of peptide **5**.

Replicate	k_{on} (M^{-1} s^{-1})	k_{off} (s^{-1})	K_D (pM)	R_{MAX} (RU)	t_C (RU M^{-1} s^{-1})	Chi^2 (RU^2)	U-value
**1**	$1.453 \pm 0.003 \times 10^6$	$7.105 \pm 0.084 \times 10^{-5}$	48.9	46.6 ± 0.02	$1.410 \pm 3.800 \times 10^{13}$	1.390	7
**2**	$1.461 \pm 0.003 \times 10^6$	$7.493 \pm 0.082 \times 10^{-5}$	51.3	47.4 ± 0.02	$7.999 \pm 8.400 \times 10^{12}$	1.370	7
**3**	$1.711 \pm 0.003 \times 10^6$	$8.018 \pm 0.068 \times 10^{-5}$	46.9	40.1 ± 0.02	$3.497 \pm 88.00 \times 10^{13}$	0.627	5

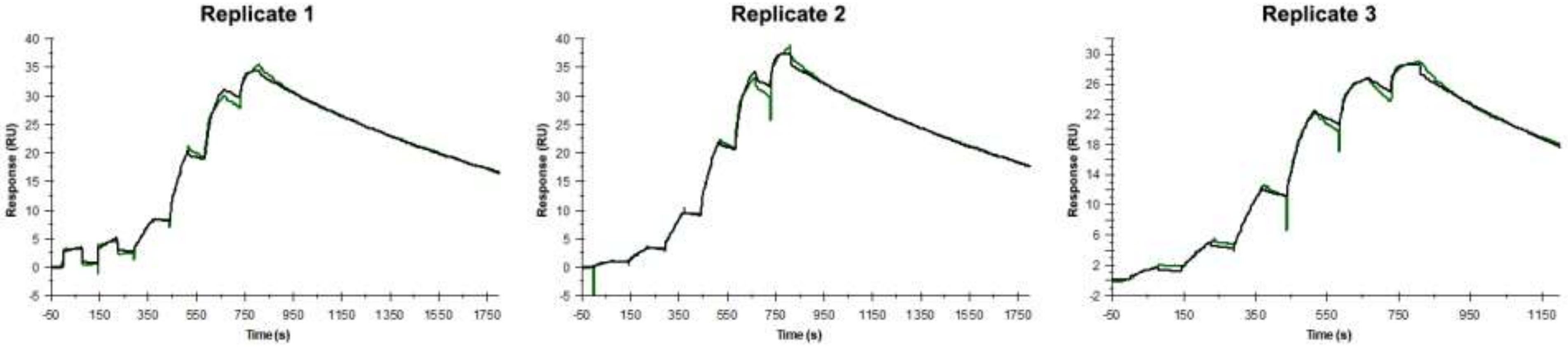

Figure S19. Sensorgrams obtained for each replicate of peptide **6** / Phd_{STm}^{52-73} F56A (analyte) binding to Doc_{STm} (ligand). Six concentrations of the analyte were flown over the surface in a single cycle, ranging from 100 nM to 0.412 nM (3-fold dilutions). In all replicates the association time was 80s. The dissociation times were 1000 s for replicates 1 and 2 and 400 s for replicate 3.

Table S17. Summary of the fitted parameters obtained from each SPR measurement of peptide **6**.

Replicate	k_{on} (M^{-1} s^{-1})	k_{off} (s^{-1})	K_D (pM)	R_{MAX} (RU)	t_C (RU M^{-1} s^{-1})	Chi^2 (RU^2)	U-value
**1**	$7.135 \pm 0.010 \times 10^5$	$7.167 \pm 0.008 \times 10^{-4}$	1000	34.1 ± 0.01	$1.322 \pm 0.850 \times 10^{13}$	0.285	1
**2**	$7.641 \pm 0.010 \times 10^5$	$7.021 \pm 0.007 \times 10^{-4}$	919	35.8 ± 0.01	$5.990 \pm 3.730 \times 10^{10}$	0.257	1
**3**	$14.90 \pm 0.03 \times 10^5$	$11.10 \pm 0.03 \times 10^{-4}$	745	27.6 ± 0.01	$2.910 \pm 2.760 \times 10^{13}$	0.237	1

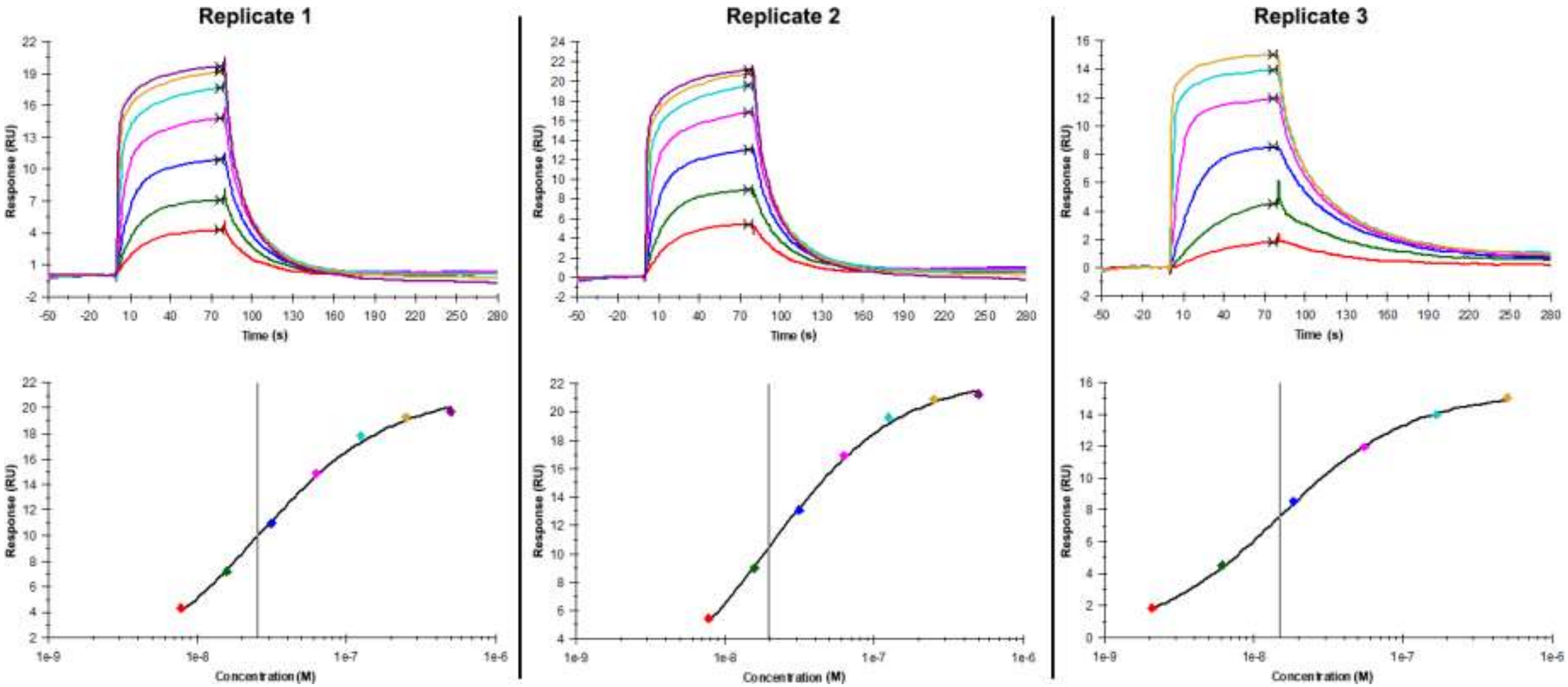

Figure S20. Sensorgrams (upper charts) and steady-state fit (lower charts) obtained for each replicate of peptide **7** / $Phd_{STm}^{52\text{-}73}$ I59A (analyte) binding to Doc_{STm} (ligand). The analyte was flown in multiple individual cycles, ranging from 500 nM to 7.8125 nM (2-fold dilutions) for replicates 1 and 2 and from 500 nM to 2.06 nM (3-fold dilutions) for replicate 3. The association and dissociation times were 80 s and 200 s, respectively.

Table S18. Summary of the fitted parameters obtained from each SPR measurement of peptide **7**.

Replicate	K_D (nM)	R_{MAX} (RU)	Offset (RU)	Chi^2 (RU^2)
**1**	25.40 ± 3.00	22.3 ± 0.7	-1.20 ± 0.86	0.113
**2**	19.35 ± 2.20	24.1 ± 0.9	-1.70 ± 1.00	0.099
**3**	15.10 ± 0.09	15.4 ± 0.2	0.00 ± 0.22	0.021

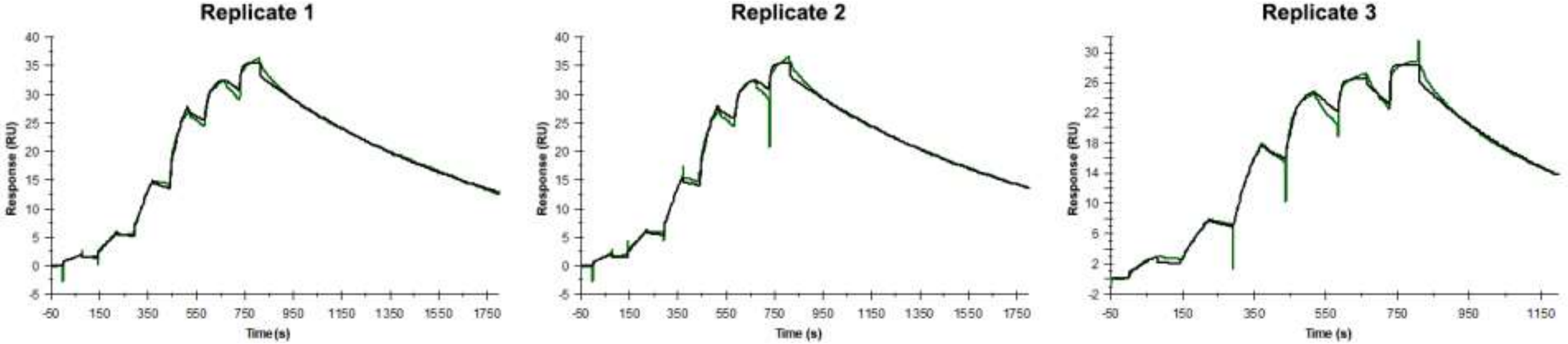

Figure S21. Sensorgrams obtained for each replicate of peptide **8** / $Phd_{STm}^{52\text{-}73}$ M60A (analyte) binding to Doc_{STm} (ligand). Six concentrations of the analyte were flown over the surface in a single cycle, ranging from 100 nM to 0.412 nM (3-fold dilutions). The association and dissociation times were 80 s and 1000 s, respectively.

Table S19. Summary of the fitted parameters obtained from each SPR measurement of peptide **8**.

Replicate	k_{on} (M^{-1} s^{-1})	k_{off} (s^{-1})	K_D (pM)	R_{MAX} (RU)	t_C (RU M^{-1} s^{-1})	Chi^2 (RU^2)	U-value
**1**	$1.442 \pm 0.002 \times 10^6$	$9.658 \pm 0.008 \times 10^{-4}$	670	33.6 ± 0.01	$1.566 \pm 1.000 \times 10^{13}$	0.275	1
**2**	$1.490 \pm 0.003 \times 10^6$	$9.101 \pm 0.010 \times 10^{-4}$	611	33.5 ± 0.01	$3.717 \pm 4.600 \times 10^{13}$	0.418	1
**3**	$3.730 \pm 0.016 \times 10^6$	$18.10 \pm 0.06 \times 10^{-4}$	485	26.3 ± 0.01	$8.970 \pm 0.178 \times 10^7$	0.248	1

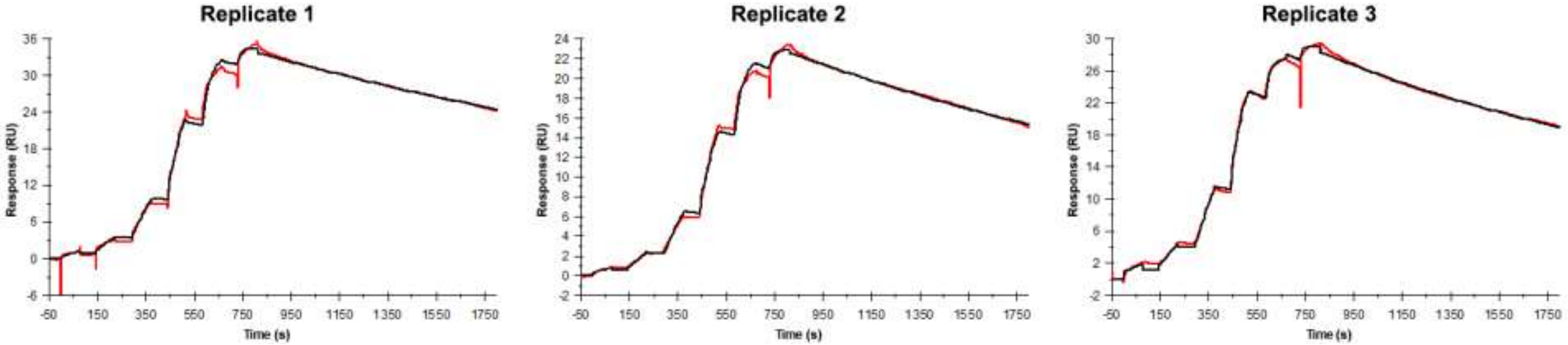

Figure S22. Sensorgrams obtained for each replicate of peptide **9** / $Phd_{STm}^{52\text{-}73}$ V62A (analyte) binding to Doc_{STm} (ligand). Six concentrations of the analyte were flown over the surface in a single cycle, ranging from 50 nM to 0.206 nM (3-fold dilutions). The association and dissociation times were 80 s and 1000 s, respectively.

Table S20. Summary of the fitted parameters obtained from each SPR measurement of peptide **9**.

Replicate	k_{on} (M^{-1} s^{-1})	k_{off} (s^{-1})	K_D (pM)	R_{MAX} (RU)	t_C (RU M^{-1} s^{-1})	Chi^2 (RU^2)	U-value
**1**	$1.798 \pm 0.011 \times 10^6$	$3.293 \pm 0.008 \times 10^{-4}$	183	33.8 ± 0.01	$2.110 \pm 0.220 \times 10^8$	0.365	1
**2**	$1.649 \pm 0.002 \times 10^6$	$3.936 \pm 0.006 \times 10^{-4}$	239	22.7 ± 0.01	$1.087 \pm 0.230 \times 10^{13}$	0.102	1
**3**	$3.350 \pm 0.015 \times 10^6$	$4.420 \pm 0.008 \times 10^{-4}$	132	28.4 ± 0.01	$6.920 \pm 0.112 \times 10^7$	0.151	1

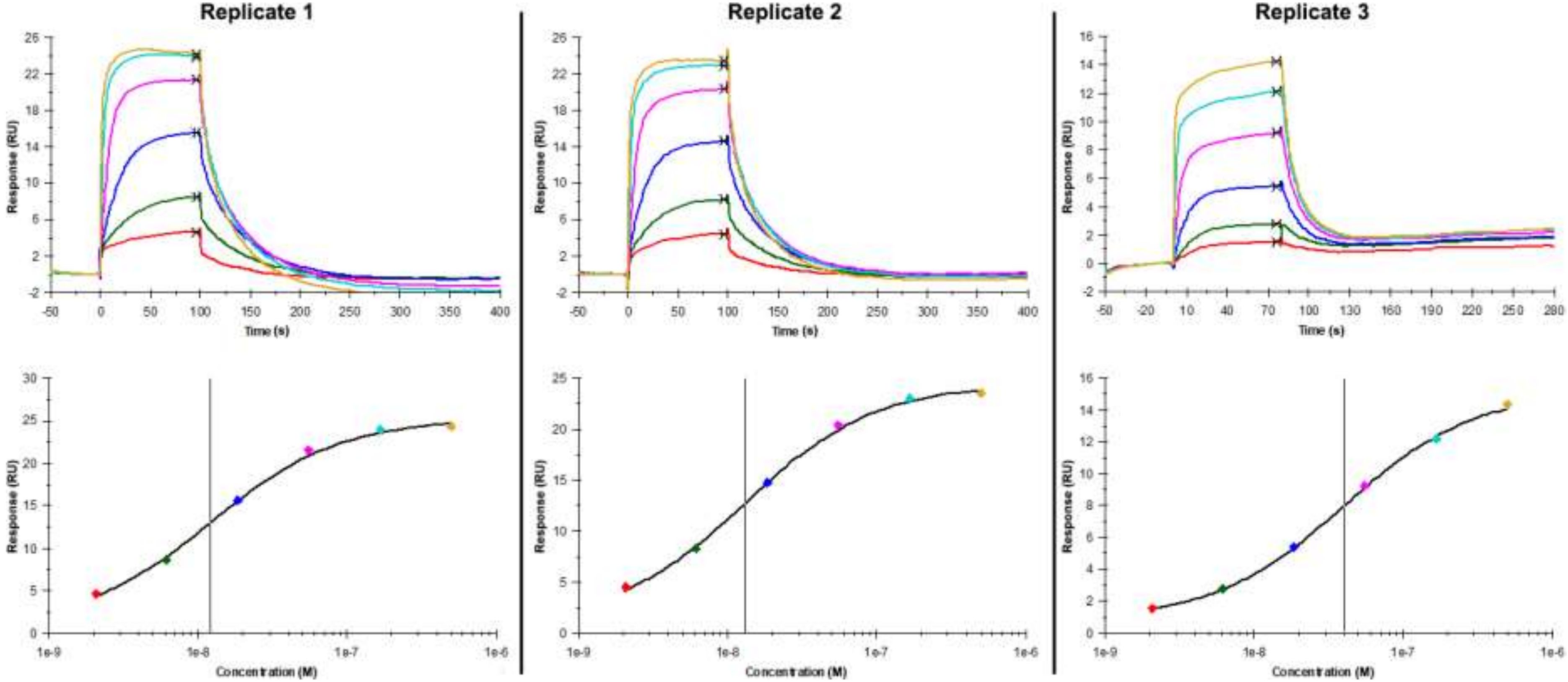

Figure S23. Sensorgrams (upper charts) and steady-state fit (lower charts) obtained for each replicate of peptide **10** / Phd_{STm}^{52-73} H63A (analyte) binding to Doc_{STm} (ligand). Six concentrations of the analyte were flown in multiple individual cycles, ranging from 500 nM to 2.06 nM (3-fold dilutions). The association and dissociation times were, respectively, 100 s and 300 s. For replicate 3 the association and dissociation times were, respectively, 80 s and 200 s

Table S21. Summary of the fitted parameters obtained from each SPR measurement of peptide **10**.

Replicate	K_D (nM)	R_{MAX} (RU)	Offset (RU)	Chi^2 (RU^2)
**1**	12.17 ± 1.8	24.5 ± 0.9	0.80 ± 0.97	0.318
**2**	13.08 ± 1.40	23.4 ± 0.6	1.00 ± 0.67	0.168
**3**	40.00 ± 2.87	14.3 ± 0.2	0.80 ± 0.18	0.032

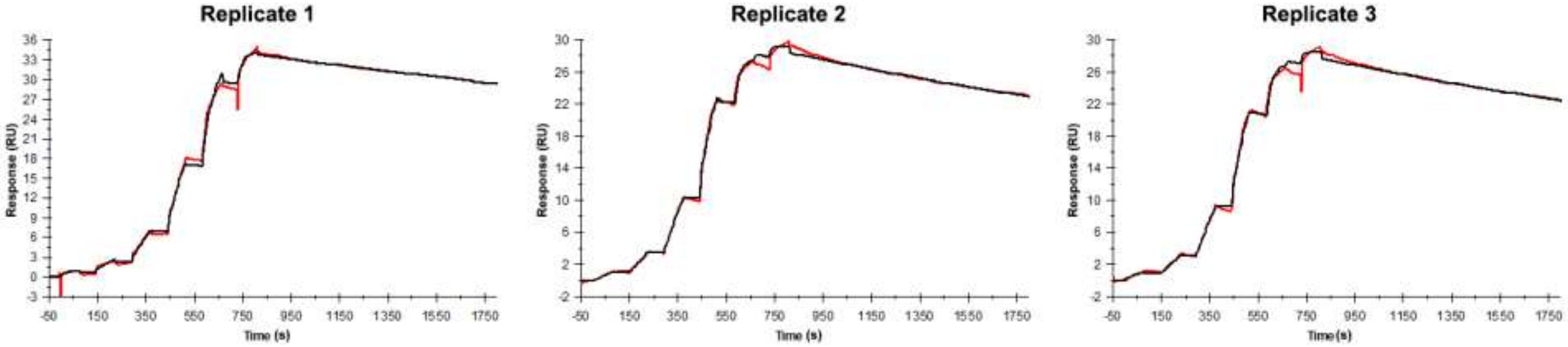

Figure S24. Sensorgrams obtained for each replicate of peptide **11** / Phd$_{STm}$$^{52\text{-}73}$ G64A (analyte) binding to Doc$_{STm}$ (ligand). Six concentrations of the analyte were flown over the surface in a single cycle, ranging from 50 nM to 0.206 nM (3-fold dilutions). The association and dissociation times were 80 s and 1000 s, respectively.

Table S22. Summary of the fitted parameters obtained from each SPR measurement of peptide **11**.

Replicate	k_{on} (M^{-1} s^{-1})	k_{off} (s^{-1})	K_D (pM)	R_{MAX} (RU)	t_C (RU M^{-1} s^{-1})	Chi^2 (RU^2)	U-value
**1**	$1.081 \pm 0.001 \times 10^6$	$1.420 \pm 0.004 \times 10^{-5}$	130	33.8 ± 0.01	$4.702 \pm 3.400 \times 10^{11}$	0.156	2
**2**	$3.070 \pm 0.014 \times 10^6$	$2.280 \pm 0.005 \times 10^{-5}$	74.3	28.4 ± 0.01	$5.270 \pm 0.073 \times 10^7$	0.125	1
**3**	$2.900 \pm 0.016 \times 10^6$	$2.230 \pm 0.006 \times 10^{-5}$	76.9	27.7 ± 0.01	$4.240 \pm 0.062 \times 10^7$	0.143	1

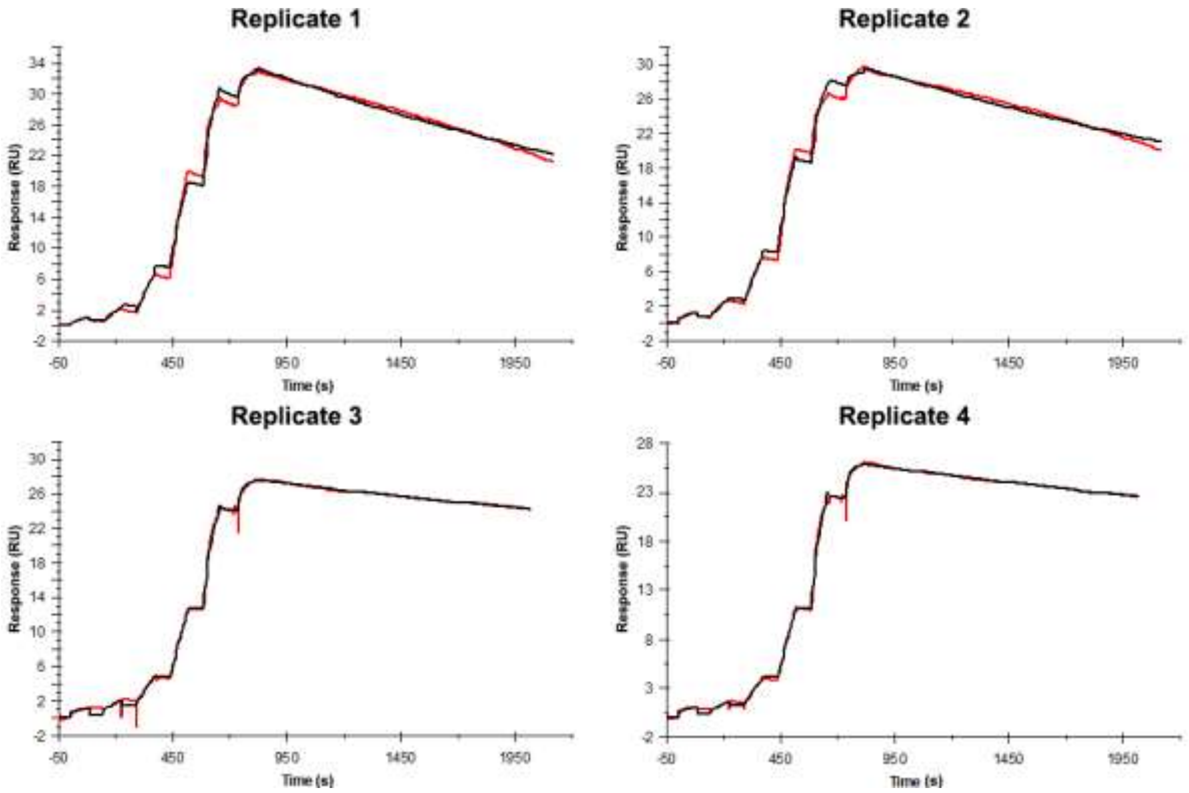

Figure S25. Sensorgrams obtained for each replicate of peptide **12** / Phd_{STm}^{52-73} N65A (analyte) binding to Doc_{STm} (ligand). Six concentrations of the analyte were flown over the surface in a single cycle, ranging from 50 nM to 0.206 nM (3-fold dilutions). In all replicates the association time was 80s. The dissociation times were 1300 s for replicates 1 and 2, and 1200 s for replicates 3 and 4.

Table S23. Summary of the fitted parameters obtained from each SPR measurement of peptide **12**.

Replicate	k_{on} (M^{-1} s^{-1})	k_{off} (s^{-1})	K_D (pM)	R_{MAX} (RU)	t_C (RU M^{-1} s^{-1})	Chi^2 (RU^2)	U-value
**1**	$1.259 \pm 0.002 \times 10^6$	$3.155 \pm 0.005 \times 10^{-4}$	251	33.5 ± 0.01	$7.047 \pm 1.700 \times 10^{12}$	0.338	1
**2**	$1.595 \pm 0.003 \times 10^6$	$2.643 \pm 0.005 \times 10^{-4}$	166	29.7 ± 0.01	$4.890 \pm 1.900 \times 10^{12}$	0.311	1
**3**	$1.290 \pm 0.004 \times 10^6$	$1.100 \pm 0.003 \times 10^{-4}$	85.3	27.6 ± 0.01	$2.410 \pm 0.026 \times 10^7$	0.077	2
**4**	$1.440 \pm 0.004 \times 10^6$	$1.200 \pm 0.002 \times 10^{-4}$	83.3	25.9 ± 0.01	$1.360 \pm 0.006 \times 10^7$	0.032	1

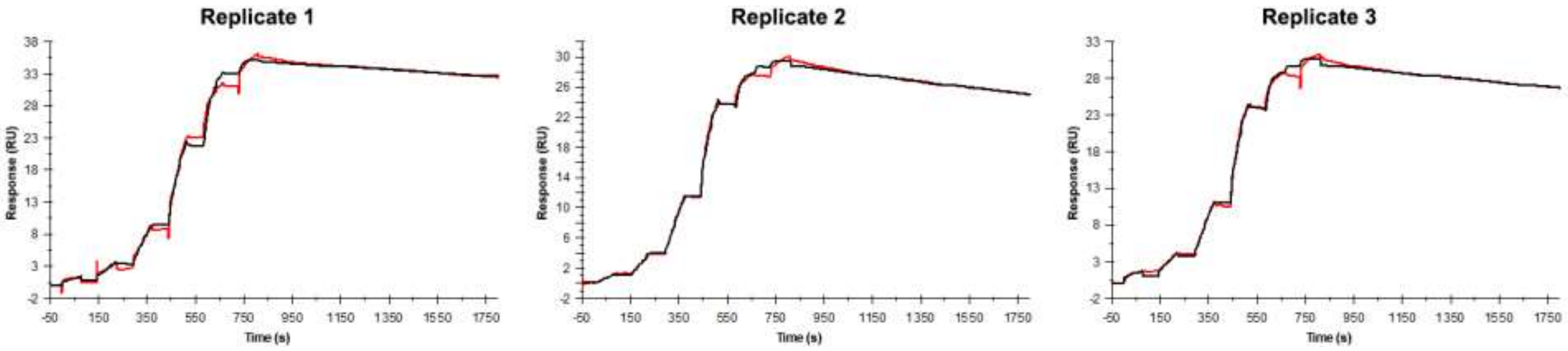

Figure S26. Sensorgrams obtained for each replicate of peptide **13** / $Phd_{STm}^{52\text{-}73}$ E66A (analyte) binding to Doc_{STm} (ligand). Six concentrations of the analyte were flown over the surface in a single cycle, ranging from 50 nM to 0.206 nM (3-fold dilutions). The association and dissociation times were 80 s and 1000 s, respectively.

Table S24. Summary of the fitted parameters obtained from each SPR measurement of peptide **13**.

Replicate	k_{on} (M^{-1} s^{-1})	k_{off} (s^{-1})	K_D (pM)	R_{MAX} (RU)	t_C (RU M^{-1} s^{-1})	Chi^2 (RU^2)	U-value
**1**	$1.504 \pm 0.002 \times 10^6$	$7.151 \pm 0.063 \times 10^{-5}$	47.5	35.0 ± 0.01	$4.63 \pm 12.00 \times 10^{12}$	0.406	5
**2**	$3.260 \pm 0.013 \times 10^6$	$14.70 \pm 0.04 \times 10^{-5}$	45.1	28.9 ± 0.01	$6.760 \pm 0.094 \times 10^7$	0.111	2
**3**	$3.350 \pm 0.017 \times 10^6$	$11.80 \pm 0.05 \times 10^{-5}$	35.2	29.9 ± 0.01	$4.830 \pm 0.058 \times 10^7$	0.159	2

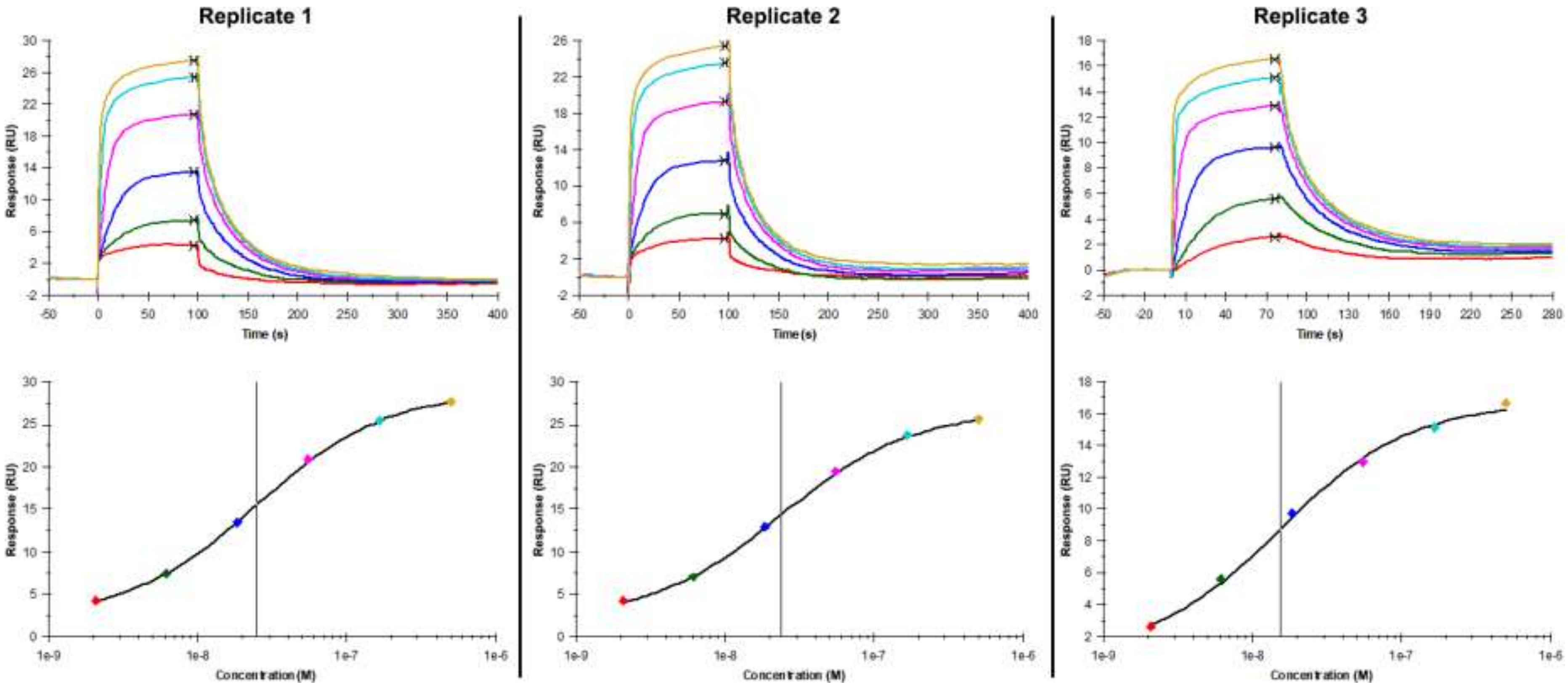

Figure S27. Sensorgrams (upper charts) and steady-state fit (lower charts) obtained for each replicate of peptide **14** / $Phd_{STm}^{52\text{-}73}$ L67A (analyte) binding to Doc_{STm} (ligand). Six concentrations of the analyte were flown in multiple individual cycles, ranging from 500 nM to 2.06 nM (3-fold dilutions). The association and dissociation times were, respectively, 100 s and 300 s. For replicate 3 the association and dissociation times were, respectively, 80 s and 200 s

Table S25. Summary of the fitted parameters obtained from each SPR measurement of peptide **14**.

Replicate	K_D (nM)	R_{MAX} (RU)	Offset (RU)	Chi^2 (RU^2)
**1**	24.96 ± 0.50	26.8 ± 0.1	2.10 ± 0.11	0.009
**2**	23.93 ± 0.94	24.5 ± 0.2	2.10 ± 0.20	0.028
**3**	15.4 ± 2.13	15.8 ± 0.5	0.90 ± 0.54	0.131

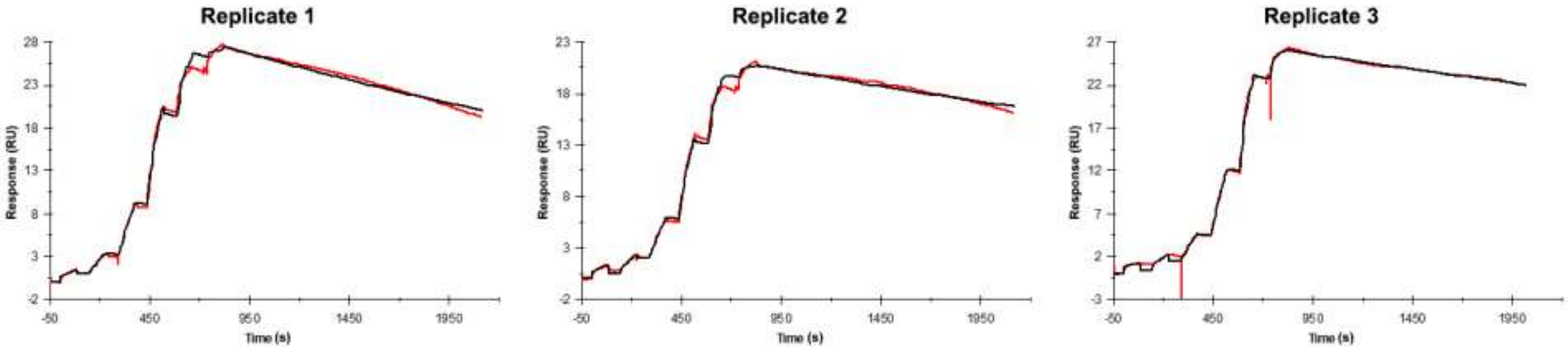

Figure S28. Sensorgrams obtained for each replicate of peptide **15** / Phd_{STm}^{52-73} R68A (analyte) binding to Doc_{STm} (ligand). Six concentrations of the analyte were flown over the surface in a single cycle, ranging from 50 nM to 0.206 nM (3-fold dilutions). In all replicates the association time was 80s. The dissociation times were 1300 s for replicate 1 and 2, and 1200 s for replicate 3.

Table S26. Summary of the fitted parameters obtained from each SPR measurement of peptide **15**.

Replicate	k_{on} (M^{-1} s^{-1})	k_{off} (s^{-1})	K_D (pM)	R_{MAX} (RU)	t_C (RU M^{-1} s^{-1})	Chi^2 (RU^2)	U-value
**1**	$1.951 \pm 0.003 \times 10^6$	$2.436 \pm 0.005 \times 10^{-4}$	124	27.6 ± 0.01	$6.39 \pm 22.00 \times 10^{12}$	0.245	1
**2**	$1.566 \pm 0.002 \times 10^6$	$1.662 \pm 0.004 \times 10^{-4}$	106	20.8 ± 0.01	$8.020 \pm 2.600 \times 10^{12}$	0.118	2
**3**	$1.290 \pm 0.004 \times 10^6$	$1.440 \pm 0.003 \times 10^{-4}$	112	26.1 ± 0.01	$2.240 \pm 0.024 \times 10^7$	0.073	1

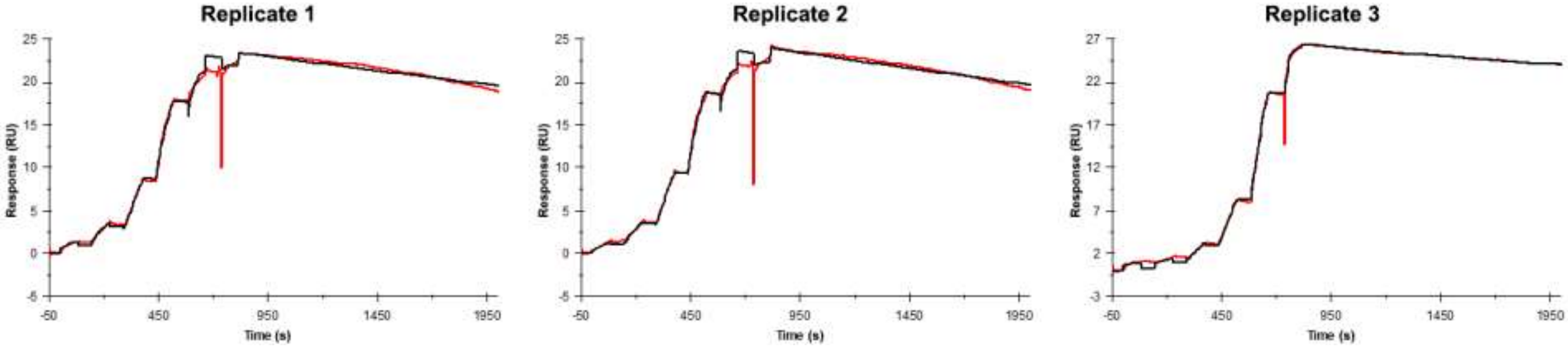

Figure S29. Sensorgrams obtained for each replicate of peptide **16** / Phd_{STm}^{52-73} E69A (analyte) binding to Doc_{STm} (ligand). Six concentrations of the analyte were flown over the surface in a single cycle, ranging from 50 nM to 0.206 nM (3-fold dilutions). The association and dissociation times were 80 s and 1200 s, respectively.

Table S27. Summary of the fitted parameters obtained from each SPR measurement of peptide **16**.

Replicate	k_{on} (M^{-1} s^{-1})	k_{off} (s^{-1})	K_D (pM)	R_{MAX} (RU)	t_C (RU M^{-1} s^{-1})	Chi^2 (RU^2)	U-value
**1**	$2.199 \pm 0.005 \times 10^{6}$	$1.511 \pm 0.007 \times 10^{-4}$	68.7	23.4 ± 0.01	$2.47 \pm 30.00 \times 10^{14}$	0.310	3
**2**	$2.373 \pm 0.005 \times 10^{6}$	$1.688 \pm 0.007 \times 10^{-4}$	70.3	24.0 ± 0.01	$9.908 \pm 24.000 \times 10^{14}$	0.348	2
**3**	$1.730 \pm 0.011 \times 10^{6}$	$0.8970 \pm 0.0037 \times 10^{-4}$	51.8	26.4 ± 0.01	$6.090 \pm 0.029 \times 10^{6}$	0.079	2

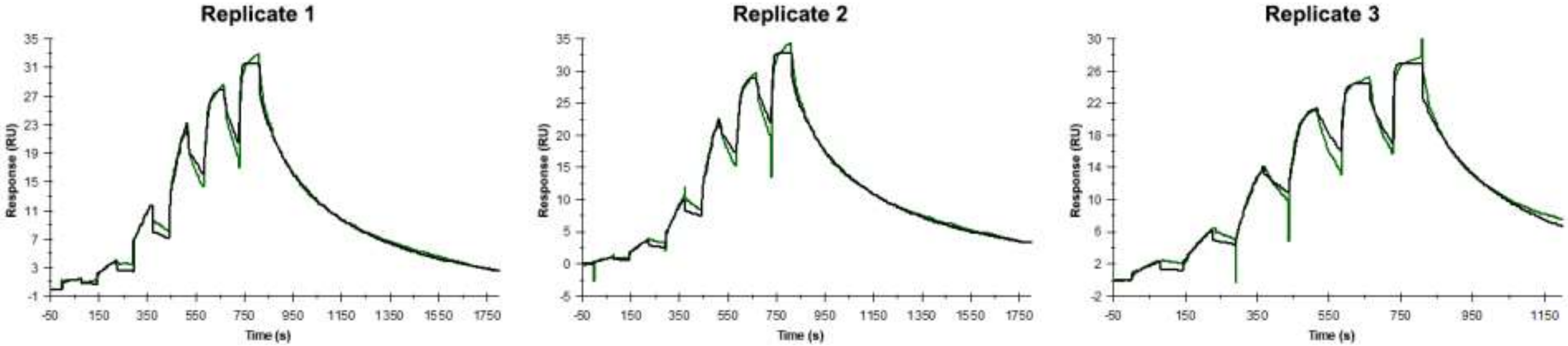

Figure S30. Sensorgrams obtained for each replicate of peptide **17** / Phd_{STm}^{52-73} L70A (analyte) binding to Doc_{STm} (ligand). Six concentrations of the analyte were flown over the surface in a single cycle, ranging from 100 nM to 0.412 nM (3-fold dilutions). In all replicates the association time was 80s. The dissociation times were 1000 s for replicates 1 and 2 and 400 s for replicate 3.

Table S28. Summary of the fitted parameters obtained from each SPR measurement of peptide **17**.

Replicate	k_{on} (M^{-1} s^{-1})	k_{off} (s^{-1})	K_D (nM)	R_{MAX} (RU)	t_C (RU M^{-1} s^{-1})	Chi^2 (RU^2)	U-value
**1**	$2.873 \pm 0.027 \times 10^6$	$6.019 \pm 0.059 \times 10^{-3}$	2.10	28.4 ± 0.04	$1.078 \pm 0.008 \times 10^7$	0.464	2
**2**	$3.197 \pm 0.035 \times 10^6$	$6.343 \pm 0.075 \times 10^{-3}$	1.98	30.3 ± 0.04	$1.034 \pm 0.008 \times 10^7$	0.584	2
**3**	$5.440 \pm 0.089 \times 10^6$	$5.540 \pm 0.079 \times 10^{-3}$	1.02	23.2 ± 0.04	$2.390 \pm 0.044 \times 10^7$	0.730	3

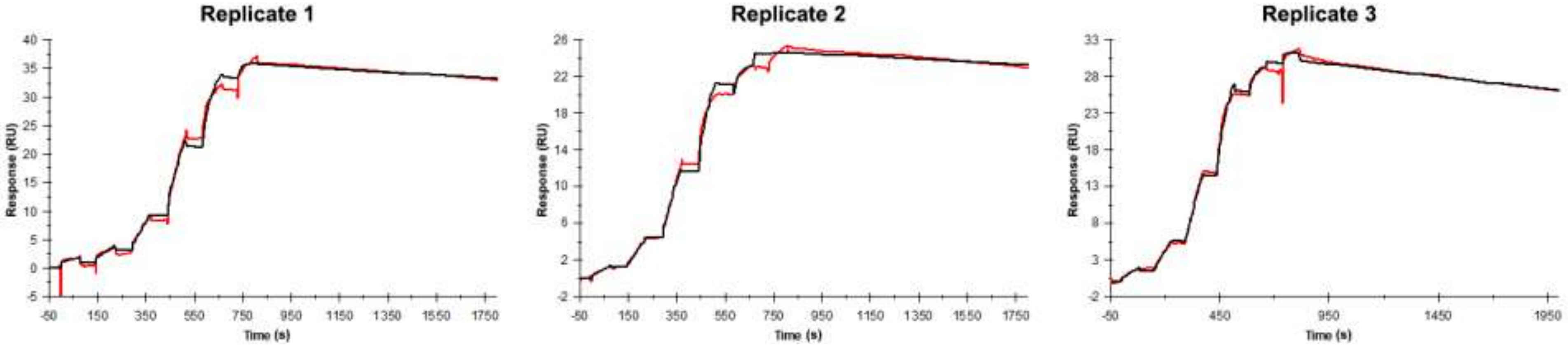

Figure S31. Sensorgrams obtained for each replicate of peptide **18** / $Phd_{STm}^{52\text{-}73}$ D72A (analyte) binding to Doc_{STm} (ligand). Six concentrations of the analyte were flown over the surface in a single cycle, ranging from 50 nM to 0.206 nM (3-fold dilutions). In all replicates the association time was 80s. The dissociation times were 1000 s for replicates 1 and 2 and 1200 s for replicate 3.

Table S29. Summary of the fitted parameters obtained from each SPR measurement of peptide **18**.

**Replicate**	**k_{on} (M^{-1} s^{-1})**	**k_{off} (s^{-1})**	**K_D (pM)**	**R_{MAX} (RU)**	**t_C (RU M^{-1} s^{-1})**	**Chi^2 (RU^2)**	**U-value**
**1**	$1.397 \pm 0.002 \times 10^6$	$0.7430 \pm 0.0069 \times 10^{-4}$	53.2	35.7 ± 0.01	$1.189 \pm 1.800 \times 10^{15}$	0.506	5
**2**	$3.007 \pm 0.005 \times 10^6$	$0.5670 \pm 0.0066 \times 10^{-4}$	18.9	24.7 ± 0.01	$7.652 \pm 15.000 \times 10^{12}$	0.245	7
**3**	$3.120 \pm 0.003 \times 10^6$	$1.200 \pm 0.003 \times 10^{-4}$	38.5	30.2 ± 0.01	$5.850 \pm 5.030 \times 10^{13}$	0.142	2

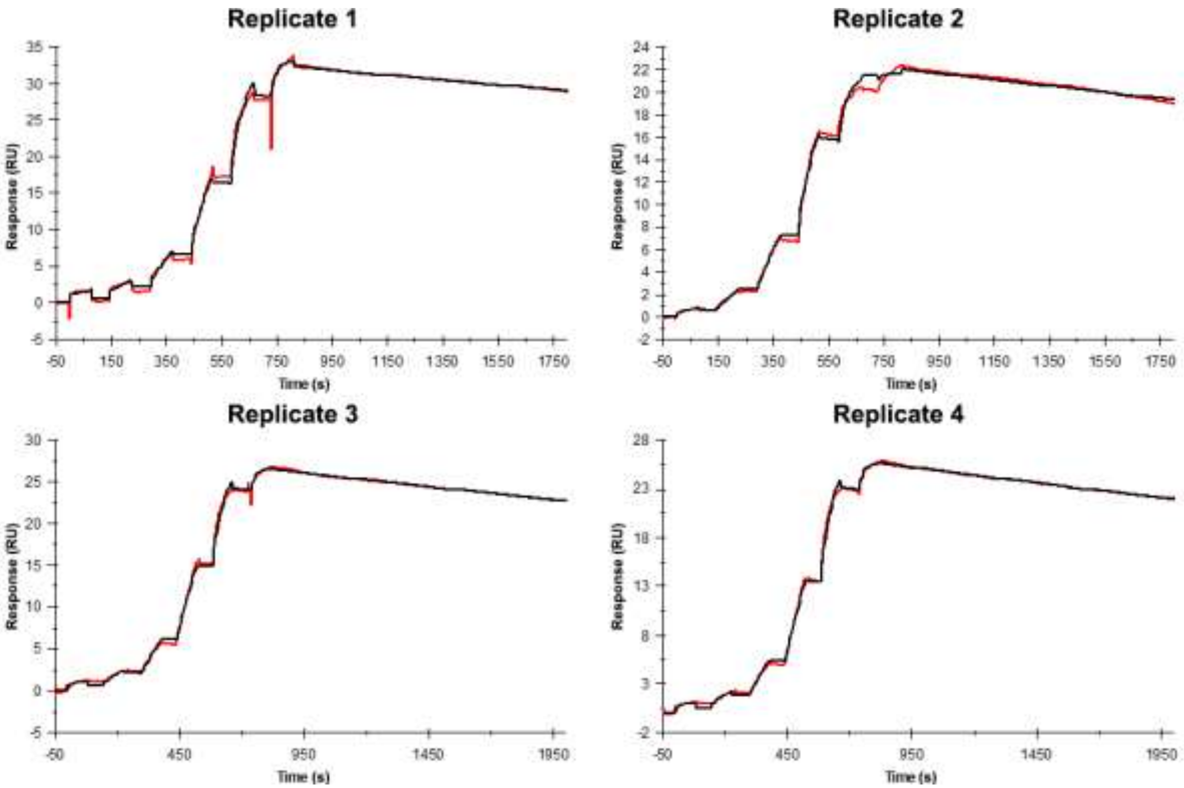

Figure S32. Sensorgrams obtained for each replicate of peptide **19** / Phd_{STm}^{52-73} K73A (analyte) binding to Doc_{STm} (ligand). Six concentrations of the analyte were flown over the surface in a single cycle, ranging from 50 nM to 0.206 nM (3-fold dilutions). In all replicates the association time was 80s. The dissociation times were 1000 s for replicates 1 and 2, and 1200 s for replicates 3 and 4.

Table S30. Summary of the fitted parameters obtained from each SPR measurement of peptide **19**.

Replicate	k_{on} (M^{-1} s^{-1})	k_{off} (s^{-1})	K_D (pM)	R_{MAX} (RU)	t_C (RU M^{-1} s^{-1})	Chi^2 (RU^2)	U-value
**1**	$1.085 \pm 0.001 \times 10^6$	$1.092 \pm 0.005 \times 10^{-4}$	101	32.4 ± 0.01	$4.165 \pm 25.000 \times 10^{12}$	0.211	3
**2**	$2.120 \pm 0.013 \times 10^6$	$1.344 \pm 0.006 \times 10^{-4}$	63.4	22.1 ± 0.01	$9.656 \pm 0.560 \times 10^7$	0.127	2
**3**	$1.280 \pm 0.002 \times 10^6$	$1.280 \pm 0.003 \times 10^{-4}$	100	26.5 ± 0.01	$2.260 \pm 8.360 \times 10^{10}$	0.092	1
**4**	$1.280 \pm 0.004 \times 10^6$	$1.320 \pm 0.002 \times 10^{-4}$	103	25.7 ± 0.01	$6.870 \pm 0.184 \times 10^7$	0.049	1

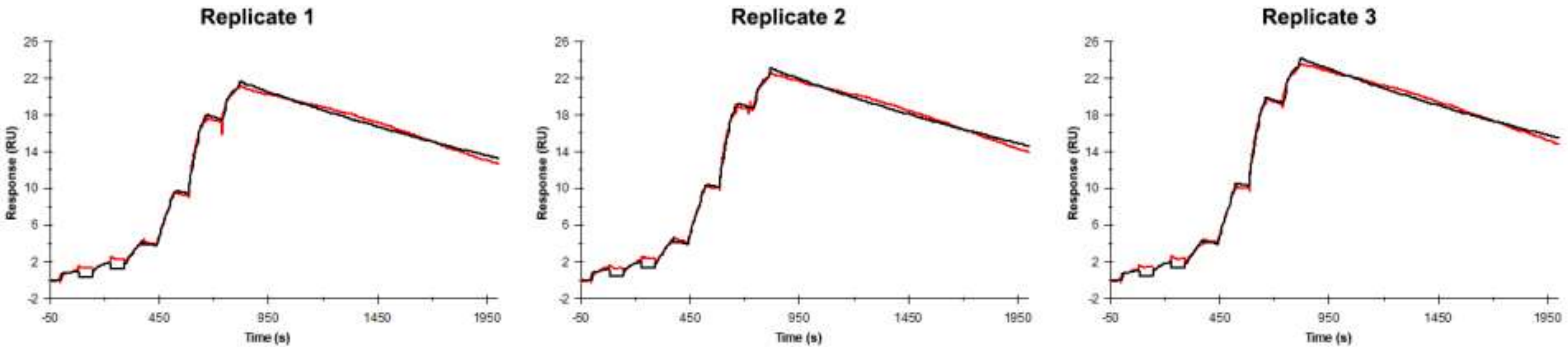

Figure S33. Sensorgrams obtained for each replicate of peptide **20** / Phd_{STm}^{52-71} (analyte) binding to Doc_{STm} (ligand). Six concentrations of the analyte were flown over the surface in a single cycle, ranging from 50 nM to 0.206 nM (3-fold dilutions). The association and dissociation times were 80 s and 1200 s, respectively.

Table S31. Summary of the fitted parameters obtained from each SPR measurement of peptide **20**.

Replicate	k_{on} (M^{-1} s^{-1})	k_{off} (s^{-1})	K_D (pM)	R_{MAX} (RU)	t_C (RU M^{-1} s^{-1})	Chi^2 (RU^2)	U-value
**1**	$9.247 \pm 0.016 \times 10^5$	$4.132 \pm 0.006 \times 10^{-4}$	447	22.0 ± 0.01	$4.539 \pm 8.000 \times 10^{12}$	0.166	1
**2**	$9.339 \pm 0.014 \times 10^5$	$3.870 \pm 0.006 \times 10^{-4}$	414	23.4 ± 0.01	$5.672 \pm 2.800 \times 10^{11}$	0.165	1
**3**	$8.910 \pm 0.014 \times 10^5$	$3.778 \pm 0.006 \times 10^{-4}$	424	24.5 ± 0.01	$8.001 \pm 11.000 \times 10^{12}$	0.164	1

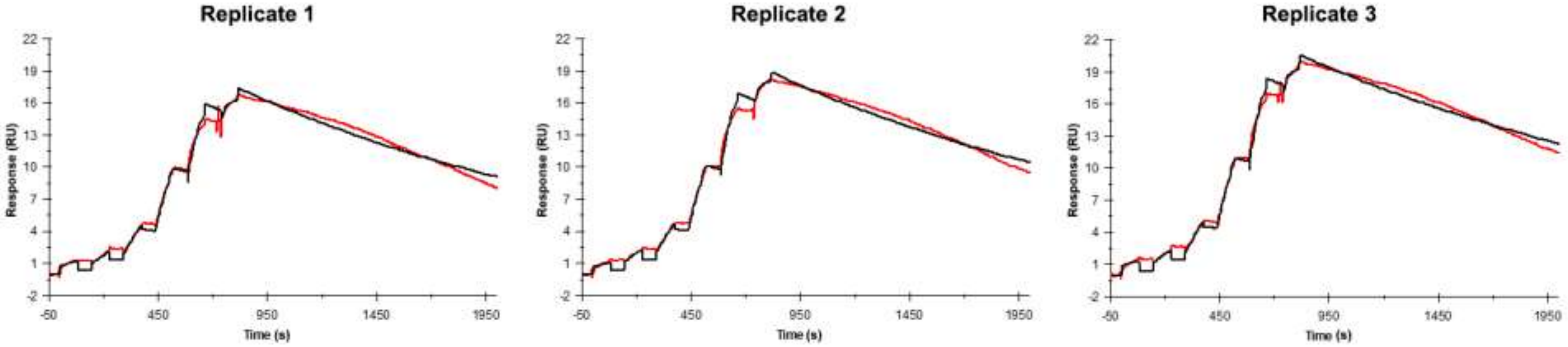

Figure S34. Sensorgrams obtained for each replicate of peptide **21** / $Phd_{STm}^{55\text{-}73}$ (analyte) binding to Doc_{STm} (ligand). Six concentrations of the analyte were flown over the surface in a single cycle, ranging from 50 nM to 0.206 nM (3-fold dilutions). The association and dissociation times were 80 s and 1200 s, respectively.

Table S32. Summary of the fitted parameters obtained from each SPR measurement of peptide **21**.

Replicate	k_{on} (M^{-1} s^{-1})	k_{off} (s^{-1})	K_D (pM)	R_{MAX} (RU)	t_C (RU M^{-1} s^{-1})	Chi^2 (RU^2)	U-value
**1**	$1.344 \pm 0.004 \times 10^6$	$5.508 \pm 0.011 \times 10^{-4}$	410	17.6 ± 0.01	$9.656 \pm 17.000 \times 10^{12}$	0.292	2
**2**	$1.218 \pm 0.003 \times 10^6$	$5.013 \pm 0.010 \times 10^{-4}$	412	19.1 ± 0.01	$2.095 \pm 1.200 \times 10^{12}$	0.287	2
**3**	$1.195 \pm 0.003 \times 10^6$	$4.361 \pm 0.009 \times 10^{-4}$	365	20.7 ± 0.01	$3.899 \pm 5.500 \times 10^{12}$	0.298	2

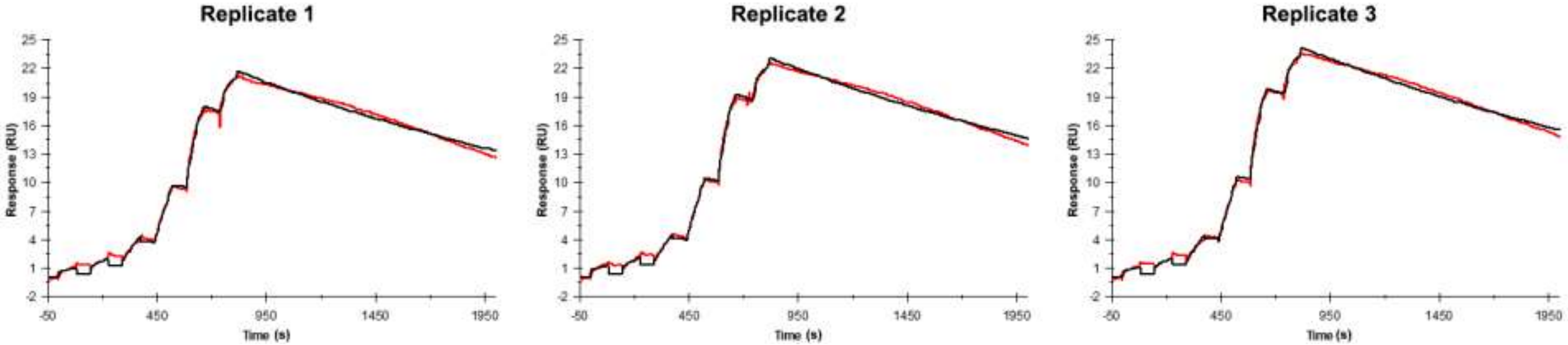

Figure S35. Sensorgrams obtained for each replicate of peptide **22** / Phd_{STm}^{55-71} (analyte) binding to Doc_{STm} (ligand). Six concentrations of the analyte were flown over the surface in a single cycle, ranging from 100 nM to 0.412 nM (3-fold dilutions). The association and dissociation times were 80 s and 800 s, respectively.

Table S33. Summary of the fitted parameters obtained from each SPR measurement of peptide **22**.

Replicate	k_{on} (M^{-1} s^{-1})	k_{off} (s^{-1})	K_D (pM)	R_{MAX} (RU)	t_C (RU M^{-1} s^{-1})	Chi^2 (RU^2)	U-value
**1**	$1.525 \pm 0.004 \times 10^6$	$5.213 \pm 0.016 \times 10^{-4}$	342	20.5 ± 0.01	$4.07 \pm 120.00 \times 10^{13}$	0.376	2
**2**	$1.466 \pm 0.004 \times 10^6$	$4.925 \pm 0.015 \times 10^{-4}$	336	21.8 ± 0.01	$1.131 \pm 2.100 \times 10^{13}$	0.381	2
**3**	$1.357 \pm 0.003 \times 10^6$	$4.946 \pm 0.015 \times 10^{-4}$	365	22.6 ± 0.01	$2.951 \pm 2.200 \times 10^{13}$	0.386	2

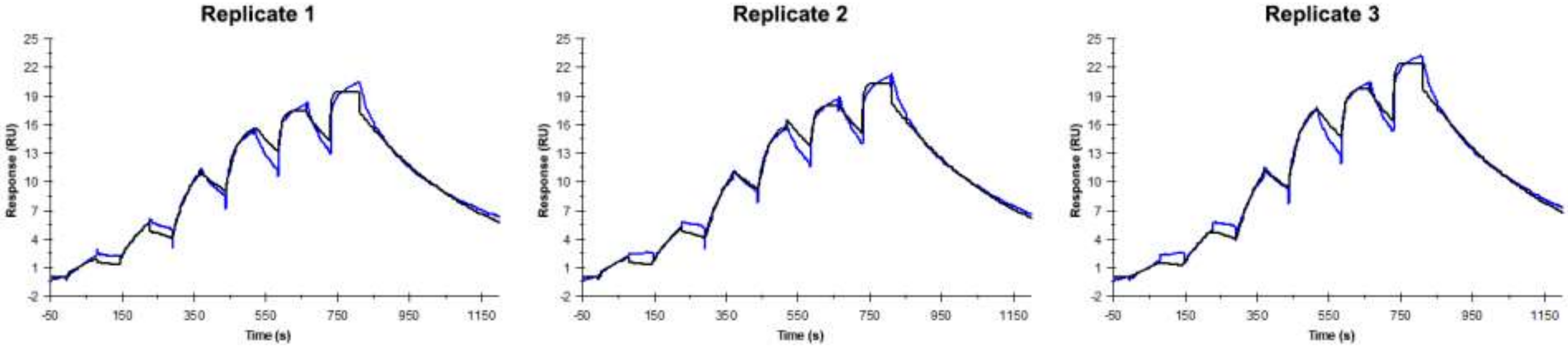

Figure S36. Sensorgrams obtained for each replicate of peptide **23** / $Phd_{STm}^{56\text{-}70}$ (analyte) binding to Doc_{STm} (ligand). Six concentrations of the analyte were flown over the surface in a single cycle, ranging from 200 nM to 0.824 nM (3-fold dilutions). The association and dissociation times were 80 s and 400 s, respectively.

Table S34. Summary of the fitted parameters obtained from each SPR measurement of peptide **23**.

Replicate	k_{on} (M^{-1} s^{-1})	k_{off} (s^{-1})	K_D (nM)	R_{MAX} (RU)	t_C (RU M^{-1} s^{-1})	Chi^2 (RU^2)	U-value
**1**	$1.534 \pm 0.006 \times 10^6$	$2.840 \pm 0.008 \times 10^{-3}$	1.85	17.6 ± 0.01	$2.425 \pm 2.000 \times 10^{12}$	0.461	2
**2**	$1.439 \pm 0.006 \times 10^6$	$2.785 \pm 0.008 \times 10^{-3}$	1.94	18.5 ± 0.01	$1.631 \pm 3.300 \times 10^{12}$	0.455	2
**3**	$1.265 \pm 0.005 \times 10^6$	$2.758 \pm 0.007 \times 10^{-3}$	2.18	20.2 ± 0.01	$4.966 \pm 42.000 \times 10^{11}$	0.457	2

6. Online data repository

Table S35. DOIs for the individual folders for access to data for each peptide or individual experiments online data repository (DOI: 10.14469/hpc/7924).

**Collection Name**	**DOI**
Peptide **1**	10.14469/hpc/7925
Peptide **2**	10.14469/hpc/7926
Peptide **3**	10.14469/hpc/7927
Peptide **4**	10.14469/hpc/7928
Peptide **5**	10.14469/hpc/7929
Peptide **6**	10.14469/hpc/7930
Peptide **7**	10.14469/hpc/7931
Peptide **8**	10.14469/hpc/7932
Peptide **9**	10.14469/hpc/7933
Peptide **10**	10.14469/hpc/7934
Peptide **11**	10.14469/hpc/7935
Peptide **12**	10.14469/hpc/7936
Peptide **13**	10.14469/hpc/7937
Peptide **14**	10.14469/hpc/7938
Peptide **15**	10.14469/hpc/7939
Peptide **16**	10.14469/hpc/7940
Peptide **17**	10.14469/hpc/7941
Peptide **18**	10.14469/hpc/7942
Peptide **19**	10.14469/hpc/7943
Peptide **20**	10.14469/hpc/7944
Peptide **21**	10.14469/hpc/7945
Peptide **22**	10.14469/hpc/7946
Peptide **23**	10.14469/hpc/7947
Peptide **24**	10.14469/hpc/7948
Peptide **25**	10.14469/hpc/7949
Phd_{STm} Protein	10.14469/hpc/7955
Doc_{STm} Protein	10.14469/hpc/7956
Growth Rescue Experiments – Peptides	10.14469/hpc/7957
Growth Rescue Experiments - Proteins	10.14469/hpc/10272

7. References

(1) Rycroft, J. A.; Gollan, B.; Grabe, G. J.; Hall, A.; Cheverton, A. M.; Larrouy-Maumus, G.; Hare, S. A.; Helaine, S. Activity of Acetyltransferase Toxins Involved in Salmonella Persister Formation during Macrophage Infection. *Nat. Commun.* **2018**, *9* (1), 1993. https://doi.org/10.1038/s41467-018-04472-6.

(2) Kitagawa, M.; Ara, T.; Arifuzzaman, M.; Ioka-Nakamichi, T.; Inamoto, E.; Toyonaga, H.; Mori, H. Complete Set of ORF Clones of Escherichia Coli ASKA Library (a Complete Set of E. Coli K-12 ORF Archive): Unique Resources for Biological Research. *DNA Res. Int. J. Rapid Publ. Rep. Genes Genomes* **2005**, *12* (5), 291–299. https://doi.org/10.1093/dnares/dsi012.

(3) Garcia-Pino, A.; Christensen-Dalsgaard, M.; Wyns, L.; Yarmolinsky, M.; Magnuson, R. D.; Gerdes, K.; Loris, R. Doc of Prophage P1 Is Inhibited by Its Antitoxin Partner Phd through Fold Complementation. *J. Biol. Chem.* **2008**, *283* (45), 30821–30827.

(4) Corzo, J. Time, the Forgotten Dimension of Ligand Binding Teaching. *Biochem. Mol. Biol. Educ. Bimon. Publ. Int. Union Biochem. Mol. Biol.* **2006**, *34* (6), 413–416. https://doi.org/10.1002/bmb.2006.494034062678.

(5) Garcia-Pino, A.; Balasubramanian, S.; Wyns, L.; Gazit, E.; Greve, H. de; Magnuson, R. D.; Charlier, D.; van Nuland, N. A. J.; Loris, R. Allostery and Intrinsic Disorder Mediate Transcription Regulation by Conditional Cooperativity. *Cell* **2010**, *142* (1), 101–111.

(6) De Gieter, S.; Konijnenberg, A.; Talavera, A.; Butterer, A.; Haesaerts, S.; De Greve, H.; Sobott, F.; Loris, R.; Garcia-Pino, A. The Intrinsically Disordered Domain of the Antitoxin Phd Chaperones the Toxin Doc against Irreversible Inactivation and Misfolding. *J. Biol. Chem.* **2014**, *289* (49), 34013–34023. https://doi.org/10.1074/jbc.M114.572396.
